# Meta-Analysis of Rates and Risk Factors for Local Recurrence in Surgically Resected Patients With NSCLC and Differences Between Asian and Non-Asian Populations

**DOI:** 10.1016/j.jtocrr.2023.100515

**Published:** 2023-04-06

**Authors:** John M. Varlotto, Cristina Bosetti, Dwight Bronson, Claudia Santucci, Maria Vitttoria Chiaruttini, Marco Scardapane, Minesh Mehta, David Harpole, Raymond Osarogiagbon, Gerald Hodgkinson

**Affiliations:** a Department of Oncology, Edwards Comprehensive Cancer Center/Marshall University, Huntington, West Virginia; b Instituto di Ricerche Farmacologiche Mario Negri Istituto di Ricovero e Cura a Carattere Scientifico (IRCCS), Milan, Italy; c Medtronic plc, Minneapolis, Minnesota; d Department of Radiation Oncology, Herbert Wertheim College of Medicine, Miami, Florida; e Department of Surgery, Duke University, Raleigh, North Carolina; f Department of Hematology and Oncology, Baptist Cancer Center, Memphis, Tennessee

**Keywords:** Locoregional recurrence, Meta-analysis, Non–small cell lung cancer, Recurrence rates, Risk factors, Surgical resection

## Abstract

**Introduction:**

Postoperative radiotherapy (PORT) reduces local failure in patients with NSCLC, without a clear overall survival benefit. It is unknown whether the subsets of patients benefit. Two recent large randomized controlled trials, PORT-C (People’s Republic of China) and Lung ART (Europe), reported widely different locoregional recurrence (LR) rates in the control arms, at 18.3% and 28.1% (46% of which were mediastinal recurrences), respectively. We performed a meta-analysis of patients with pathologic (p) N0 to N2 disease to evaluate the risk factors for LR and to explore possible differences in recurrence risk between Asian population (AP) and non-Asian population (NAP).

**Methods:**

We identified all original studies of curative NSCLC surgical resection which reported risk of LR between January 1, 2000, and January 10, 2021, excluding studies with less than 10 LR, patients with metastatic disease, or any neoadjuvant therapy. A total of 87 studies were identified with pN0 to N2 disease; of these, 56 were of high quality (HQ) on the basis of the Newcastle-Ottawa Scale. For each risk factor, we derived pooled relative risk (RR) and 5-year rate estimates using random-effects models.

**Results:**

Overall, the three significant highest pooled RRs (95% confidence intervals) for LR were pN2 versus pN0 (3.01, 1.39–6.55), lymphovascular invasion (1.92, 1.58–2.33), and advanced pT3–4 stage versus pT1 (1.86, 1.53–2.25). For HQ studies, the highest RRs for LR were lymphovascular invasion (1.94, 1.57–2.40), sublobar versus lobar resection (1.86, 1.46–2.36), and pN1 versus pN0 (1.84, 1.37–2.47), but pN2 versus pN0 was no longer significant (3.0, 0.57–15.61), on the basis of only two eligible studies. The RRs for LR were consistent for most factors in AP and NAP, although the RR for male versus female sex was higher in AP (1.44, 1.21–1.72) than in NAP (1.09, 0.99–1.19). Where reported, the pooled rate of LR at 5 years was lower in AP (12.0%) than in NAP (22.7%), despite similar overall 5-year recurrence rates (both LR and distal) in both populations: 38.0% in AP and 37.3% in NAP. Nevertheless, a lower 5-year mortality rate was noted in AP (24.3%) than in NAP (45.9%).

**Conclusions:**

There is little high-quality evidence to support the hypothesis that pN2 disease is a risk factor for LR, but LR seems to be lower in Asians. Prospective evaluation of LR factors and rates may be necessary before further prospective evaluation of PORT, because it may not depend on nodal status alone. Recurrence rates may differ in Asians. The impact of mutational status and modern treatment including targeted therapies and immune checkpoint inhibitors is inadequately studied.

## Introduction

Although histologic (tumor stage, tumor size, grade) and patient-related factors (age and sex) associated with overall survival (OS) after surgical resection of NSCLC are well established,^1^^,^^2^ patterns of recurrence, specifically the factors that drive local/locoregional recurrence (LR, usually defined as occurring within the ipsilateral lung and N1–N3 nodal areas), have not been equally well evaluated. In particular, it is not clear whether the factors related to LR and distal recurrence (DR) are the same as those for OS. Furthermore, treatment-related factors such as adjuvant chemotherapy, type of resection (i.e., lobectomy versus sublobar resection), and completeness of surgical resection including thoroughness of nodal evaluation may also play a role in recurrence patterns.^3^, ^4^, ^5^ In the modern era of lung cancer management, with an increasing role for targeted agents and immune checkpoint inhibitors, the impact of these and the presence of specific mutations on patterns and risks of LR are essentially unknown. Differences in the definition of LR by different investigators complicate the assessment.^6^

The Post-Operative Radiotherapy (PORT) meta-analysis^7^ noted that postoperative radiation was associated with a small survival benefit in patients with pathologic N (pN)2 NSCLC. Nevertheless, two relatively large studies assessed nodal stage and its relationship to LR in resected NSCLC and yielded conflicting results. Isaka et al.^8^ reported pN stage as the most significant LR risk factor, with lower 5-year freedom from LR with pN2 (85.0%) compared with pN0 (96.1%), which was even lower (53.5%) with involvement of both N2 and N1 nodes. Another investigation noted that patients with pN1 and pN2 disease had progressively higher HRs and significantly higher rates of distant recurrence and death as compared with those with pN0 disease, but nodal stage had no relation to LR risk.^9^

Two recent, prospective, randomized clinical trials of PORT in completely resected pN2 NSCLC evaluated disease-free survival (DFS) as the primary end point.^10^^,^^11^ In the European Lung ART trial, neither DFS nor OS was significantly different between the treatment arms, despite decreased mediastinal relapses from 28.0% to 14.3% in all patients and from 46% to 25% in patients with recurrence.^10^ The benefits of PORT were offset by increases in cardiopulmonary-related mortality (16% versus 2%) and deaths because of chemotherapy or radiation (3% versus 0%) with most having received the now outdated three-dimensional conformal techniques as compared with intensity modulated radiation therapy (IMRT) (89% versus 11%). In the Chinese PORT-C trial, neither OS nor DFS was significantly different between arms, but there was a significant difference in LR-free survival. The 3-year LR rate was reduced with radiotherapy from 18.3% to 9.5%.^11^ Importantly, there is no increase in grades 4 to 5 toxicity in this trial, which largely used IMRT (89.3% IMRT, 10.7% three-dimensional conformal techniques). Although LR-free survival was not explicitly defined in this protocol or manuscript, it is believed that this definition likely included the comprehensive areas as defined previously. The LR rates were very different between these two trials, despite similar selection criteria. Because of differing molecular profiles of Asian and non-Asian lung cancer, the absence of analysis for mutational patterns in either trial could partially explain the differences.

We performed this meta-analysis in patients undergoing surgical resection for NSCLC to ascertain the factors related to LR and whether LR rates differ between Asian populations (AP) and non-Asian populations (NAP). We could not evaluate the impact of driver mutations as these were not performed in most studies.

## Material and Methods

### Search Strategy

The present systematic review and meta-analysis was conducted according to the Preferred Reporting Items for Systematic Review and Meta-Analysis guidelines,^12^^,^^13^ after registering the protocol on the PROSPERO (International Prospective Register of Systematic Review; number CRD420203482). An updated literature review was performed to identify all original study publications that evaluated risk factors for LR after surgical resection of NSCLC published between January 1, 2000, and January 10, 2021, and indexed in PubMed/MEDLINE. The search strings used for the literature search are provided in Supplementary Table 1. Letters, commentaries, abstracts, editorials, and posters were not considered.

### Eligibility Criteria

In the present meta-analysis, we included original study publications that satisfied the following eligibility criteria: (1) provided data on patients with stages I to III NSCLC treated with surgical resection for curative intent; (2) were prospective or retrospective observational studies or trials; (3) considered the following major risk factors for LR: sex, age, smoking status, adjuvant chemotherapy, type of resection (lobectomy, pneumonectomy, segmentectomy, wedge resection), video-assisted thoracoscopic surgery, number of lymph node resected, lymphovascular invasion (LVI), visceral pleural invasion (VPI), pathologic stage, N stage, T stage, tumor grade, tumor histology (non-adenocarcinoma vs. adenocarcinoma; non-squamous cell carcinoma vs. squamous cell carcinoma), tumor location, and tumor size; (4) noted information on LR patterns, including rates of LR based on the factors of interest and an estimate of the relative risk (RR; such as the OR, or the hazard ratio [HR]), and corresponding 95% confidence interval (CI) or sufficient information was provided to compute them; and (5) were published as original articles in English. Studies were excluded if: (1) the patient number or LR number was less than 10; (2) studies included inoperable or metastatic patients; (3) any neoadjuvant chemotherapy or any radiation was given; or (4) information on risk factors listed previously was not provided.

Two reviewers independently screened the titles or abstracts of the identified records to exclude publications that did not meet the eligibility criteria. Subsequently, full-text articles were retrieved and assessed for inclusion in the systematic review. Any disagreement was resolved by consensus among the two reviewers or with the help of a third reviewer. It should be noted that one of 548 patients and two of 261 patients in the manuscripts by Kim et al.^14^ and Chen et al.,^15^ respectively, were noted to be stage 4 patients owing to tumors in separate ipsilateral lobes, which are now classified as T4 tumors.

### Study Quality

We evaluated the quality of the studies using the Newcastle-Ottawa scale (NOSc)^16^ with each study being attributed a score from 0 (lowest quality) to 9 (highest quality). Studies with scores between 0 and 3 were classified as low quality, 4 to 6 moderate quality, and greater than or equal to 7 high quality (HQ).

### Data Extraction

For each identified study, we collected the following information: first author, publication year, study design, country, study period, total number of patients, study population, TNM edition, resection type, mean follow-up duration, end point (LR, DR, overall recurrence [OvRec] or OS), recurrence definition, diagnostic methods for recurrence, and numbers of LR or DR recurrence. For each risk factor investigated, we extracted data on HRs, ORs or survival rates, and corresponding 95% CIs (or information to derive them) for LR and, for comparative purposes, DR, OvRec, and OS. Whenever available, we retrieved estimates adjusted for multiple potential confounding factors. When the HRs, ORs, or corresponding 95% CIs were not reported, an estimate of the RR was computed from raw tabular data, survival rates, and corresponding *p* values.^17^ We also retrieved 5-year rates of LR, DR, OvRec, and OS. Two reviewers independently extracted and crosschecked the data, and disagreements were resolved by consensus or by discussion with a third reviewer. For studies with multiple publications, we abstracted data only from the most comprehensive publication.

### Statistical Analysis

For each risk factor and outcome of interest, we derived pooled RR estimates for exposed versus not exposed (or for different categories of exposures) using random effects models, to take into account the heterogeneity of risk estimates.^13^^,^^18^ Pooled RRs were provided for LR, DR, OvRec, and OS for all studies combined and separately for studies in the AP and NAP. As a sensitivity analysis, we also computed pooled RRs from HQ studies only. We also calculated pooled 5-year survival rates for LR, DR, OvRec, and OS where available among AP and NAP.

We assessed heterogeneity between studies using Cochran’s chi-square test (defining a significant heterogeneity as a *p* < 0.10) and quantified the inconsistencies using the I^2^ statistic, which expresses the percentage of total observed variability owing to heterogeneity.^13^ For the main outcome (LR) and for each risk factor, we produced forest plots to find the study-specific estimates for cohorts exposed versus not exposed and the pooled RR estimates. For risk factors with more than 10 studies, publication bias was evaluated by visual inspection of the funnel plot, and funnel plot asymmetry was quantified using the Egger’s and Begg’s tests.^19^^,^^20^

All statistical analyses were performed using the software R (version 3.6.1).

## Results

Overall, 1632 studies were screened on the basis of the abstract and title and 100 articles were considered for inclusion in the analysis (Supplementary Fig. 1). We excluded 13 studies with missing data regarding the LR end point (13 studies; Supplementary Table 2). Of the 87 studies included, the main characteristics are found in Supplementary Table 3. Median follow-up ranged between 20 and 88.9 months. Overall, 46 studies were considered on early stage (pT1–T2 N0 tumors), 12 advanced stage (patients with only node-positive disease), and 29 any stage (all surgical stages I–III). Supplementary Table 4 includes details on methods of recurrence detection, recurrence definition, and percentages of LR/DR. Crude LR ranged from 1.8% (early stage subgroup) to 30.2% (advanced-stage subgroup).

Supplementary Figure 2 reveals the funnel plots for publication bias for the different risk factors for LR, and Supplementary Table 5 reports the Begg’s and Egger’s test results. The Begg’s test noted statistically significant publication bias for tobacco smoking, whereas the Egger’s test noted bias for sex, age (continuous variable), and tumor size.

The pooled RRs for LR are outlined in Table 1. Significant risk factors for LR include male sex (RR = 1.23, 95% CI: 1.10–1.36), segmentectomy or wedge resection versus lobectomy (1.71, 1.34–2.17), sublobar resection not otherwise specified (NOS) versus lobectomy (1.84, 1.53–2.21), presence of LVI (1.92, 1.58–2.33), presence of VPI (1.73, 1.40–2.14), nonadenocarcinoma versus adenocarcinoma (1.33, 1.08–1.64), nonsquamous cell versus squamous cell (0.82, 0.71–0.9), tumor size (large versus small; 1.57, 1.29–1.90), tumor size as a continuous variable (1-cm increase; 1.11, 1.03–1.19), pathologic stages II versus I, III versus IV, and Ib to II versus Ia (1.54, 1.35–1.74; 1.50, 1.27–1.77; and 1.42, 1.18–1.71, respectively), T2 versus T1 (1.43, 1.25–1.64), T3 to T4 versus T1 (1.86, 1.53–2.25), N1 versus N0 (1.85, 1.45–2.35), N2 versus N0 (3.01, 1.39–6.55), and combined N1 to N2 versus N0 1.63 (1.30–2.04). Forest plots for each individual risk factor are provided in Supplementary Figure 3. According to the NOSc of study quality, 32 studies (36.8%) were rated as moderate quality (NOSc 4–6) and 57 studies (63.2%) were rated as HQ (NOSc 7–9) (Supplementary Fig. 4). There were no low-quality (NOSc 1–3) studies (Supplementary Table 6).

**Table 1:** Pooled RRs and Corresponding 95% CI of Selected Risk Factors for Locoregional Recurrence After Surgical Resection of NSCLC

Locoregional Recurrence Risk Factor	N. Studies	N. Patients	Pooled RR [95% CI]	Heterogeneity	
				$I^{2}$ (%)	*p* Value
Sex					
Male vs. female	27	11720/10088	1.23 [1.10–1.36]	23	0.126
Age					
High vs. low^a^	14	7252/5662	1.11 [0.95–1.31]	46	0.020
Continuous (1-y increase)	11	5964	1.00 [0.99–1.01]	68	<0.001
Tobacco smoking					
Ever vs. never	11	2082/1382	1.04 [0.83–1.31]	0	0.654
Adjuvant chemotherapy					
Yes vs. no	23	5531/11895	1.00 [0.80–1.25]	64	<0.001
Type of resection					
Pneumonectomy vs. lobectomy	11	3084/22042	0.81 [0.61–1.07]	37	0.092
Segmentectomy or wedge vs. lobectomy	24	6262/26978	1.71 [1.34–2.17]	60	<0.001
Sublobar NOS vs. lobar resection	7	1305/7175	1.84 [1.53–2.21]	0	0.849
Lobectomy					
VATS vs. open	13	1960/3400	1.06 [0.75–1.48]	60	0.002
Lymph node resected					
Number	5	1850	1.00 [0.98–1.02]	0	0.727
Lymphovascular invasion					
Yes vs. no	32	5976/14340	1.92 [1.58–2.33]	63	<0.001
(Visceral) pleural invasion					
Yes vs. no	24	4333/11612	1.73 [1.40–2.14]	76	<0.001
Tumor grade					
Moderate/poor vs. well	7	3947/680	1.16 [0.94–1.43]	0	0.471
Poor vs. well/moderate	6	386/604	1.18 [0.83–1.69]	49	0.080
Tumor histology					
Nonadenocarcinoma vs. adenocarcinoma	16	15605/16028	1.33 [1.08–1.64]	67	<0.001
Nonsquamous cell vs. squamous cell	21	22251/12516	0.82 [0.71–0.96]	52	0.002
Tumor location					
Other location vs. right upper lobe	5	1772/1020	1.00 [0.77–1.30]	50	0.073
Left lobe vs. right lobe	6	1049/1815	1.09 [0.86–1.39]	24	0.243
Tumor size					
Large vs. small^b^	17	14079/6105	1.57 [1.29–1.90]	62	<0.001
Continuous (1-cm increase)	6	3100	1.11 [1.03–1.19]	56	0.036
Pathologic stage					
II vs. I	5	1847/7124	1.54 [1.35–1.74]	0	0.661
III–IV vs. I	4	1411/6977	1.50 [1.27–1.77]	0	0.836
Ib–II vs. Ia	6	8742/4827	1.42 [1.18–1.71]	0	0.945
III–IV^c^ vs. Ia	3	2676/4248	1.88 [0.97–3.64]	28	0.253
T stage					
T2 vs. T1	9	1747/2446	1.43 [1.25–1.64]	1	0.432
T3–T4 vs. T1	5	148/499	1.86 [1.53–2.25]	0	0.421
N stage					
N1 vs. N0	9	974/5178	1.85 [1.45–2.35]	47	0.049
N2 vs. N0	3	268/1964	3.01 [1.39–6.55]	88	<0.001
N1–N2 vs. N0	12	1654/6300	1.63 [1.30–2.04]	61	0.001

CI, confidence interval; N., number; NOS, not otherwise specified; RR, relative risk; VATS, video-assisted thoracoscopic surgery.

a Publications reporting age categories below 65–75 years or above 65–75 years.

b Publications reporting tumor size categories below 1.5–4 cm or more than 1.5–4 cm.

c It should be noted that one of 548 patients and two of 261 patients in the manuscripts by Kim et al.^14^and Chen et al.^15^were noted to be stage 4 patients which were tumors in separate ipsilateral lobe which are now classified as T4 tumors.

Table 2 contains LR recurrence risk factors for the 56 studies that were considered to be HQ by the NOSc. Factors that were significantly associated with LR were male sex (1.26, 1.12–1.43), segmentectomy or wedge versus lobectomy (1.42, 1.23–1.63), sublobar NOS versus lobectomy resection (1.86, 1.46–2.36), LVI (1.94, 1.57–2.40), VPI (1.73, 1.32–2.25), nonadenocarcinoma versus adenocarcinoma (1.26, 1.00–1.61), nonsquamous cell versus squamous cell (0.81, 0.70–0.93), tumor size (large versus small; 1.56, 1.22–1.99), tumor size as a continuous variable (1-cm increase; 1.11, 1.03–1.19), pathologic stages II versus I, III versus IV, and Ib to II versus Ia (1.54, 1.25–1.89, 1.49, 1.04–2.15, and 1.40, 0.78–2.49, respectively), T2 versus T1 (1.43, 1.17–1.75), N1 versus N0 (1.84, 1.37–2.47), and combined N2 to N1 versus N0 (1.56, 1.20–2.03).

**Table 2:** Pooled RRs and Corresponding 95% CIs of Selected Risk Factors for Locoregional Recurrence After Surgical Resection of NSCLC, Considering Only High-Quality Studies

Locoregional Recurrence Risk Factor	N. Studies	N. Patients	Pooled RR (95% CI)	Heterogeneity	
				$I^{2}$ (%)	*p* Value
Sex					
Male vs. female	24	10133/8938	1.26 (1.12–1.43)	29	0.091
Age					
High vs. low^a^	11	6980/5402	1.11 (0.96–1.27)	36	0.092
Continuous (1-y increase)	10	3664	1.01 (0.99–1.03)	71	<0.001
Tobacco smoking					
Ever vs. never	10	2027/1322	1.05 (0.83–1.34)	0	0.587
Adjuvant chemotherapy					
Yes vs. no	17	4369/8836	1.021(0.77–1.33)	58	<0.001
Type of resection					
Pneumonectomy vs. lobectomy	9	2854/21,724	0.80 (0.57–1.12)	43	0.082
Segmentectomy or wedge vs. lobectomy	17	4503/23,279	1.42 (1.23–1.63)	8	0.355
Sublobar NOS vs. lobar resection	5	908/5157	1.86 (1.46–2.36)	0	0.626
Lobectomy					
VATS vs. open	11	1720/3143	1.13 (0.79–1.62)	63	0.001
Lymph node resected					
Number	4	1850	1.00 (0.98–1.02)	0	0.727
Lymphovascular invasion					
Yes vs. no	28	5364/12,072	1.94 (1.57–2.40)	64	<0.001
(Visceral) pleural invasion					
Yes vs. no	19	3561/9125	1.73 (1.32–2.25)	78	<0.001
Tumor grade					
Moderate/poor vs. well	6	3856/656	1.16 (0.93–1.46)	8	0.366
Poor vs. well/moderate	6	386/604	1.18 (0.83–1.69)	49	0.080
Tumor histology					
Non adenocarcinoma vs. adenocarcinoma	13	14,317/14,757	1.26 (1.00–1.61)	72	<0.001
Non squamous cell vs. squamous cell	19	22,169/12,418	0.81 (0.70–0.93)	49	0.004
Tumor location					
Other location vs. right upper lobe	5	1772/1020	1.00 (0.77–1.30)	50	0.073
Left lobe vs. right lobe	5	1721/963	1.08 (0.79–1.46)	36	0.166
Tumor size					
Large vs. small^b^	12	13,747/5669	1.56 (1.22–1.99)	68	<0.001
Continuous (1-cm increase)	6	3100	1.11 (1.03–1.19)	56	0.036
Pathologic stage					
II vs. I	4	1790/6680	1.54 (1.25–1.89)	0	0.583
III–IV vs. I	3	1361/6541	1.49 (1.04–2.15)	0	0.735
Ib–II vs. Ia	3	8203/4112	1.40 (0.78–2.49)	0	0.613
III–IV^c^ vs. Ia	2	2621/4000	1.77 (0.76–4.11)	60	0.114
T stage					
T2 vs. T1	6	1422/1795	1.43 (1.17–1.75)	0	0.438
T3–T4 vs. T1	3	110/446	1.17 (0.67–2.06)	0	0.609
N stage					
N1 vs. N0	8	974/5178	1.84 (1.37–2.47)	51	0.039
N2 vs. N0	2	268/1964	3.00 (0.57–15.61)	94	<0.001
N1–N2 vs. N0	11	1654/6300	1.56 (1.20–2.03)	58	0.004

CI, confidence interval; N., number; NOS, not otherwise specified; RR, relative risk; VATS, video-assisted thoracoscopic surgery.

a Publications reporting age categories below 65 to 75 years or above 65 to 75 years.

b Publications reporting tumor size categories below 1.5–4 cm or over 1.5–4 cm.

c It should be noted that one of 548 patients and two of 261 patients in the manuscripts by Kim et al.^14^and Chen et al.^15^were noted to be stage 4 patients which were tumors in separate ipsilateral lobe which are now classified as T4 tumors.

**Figure 1:**
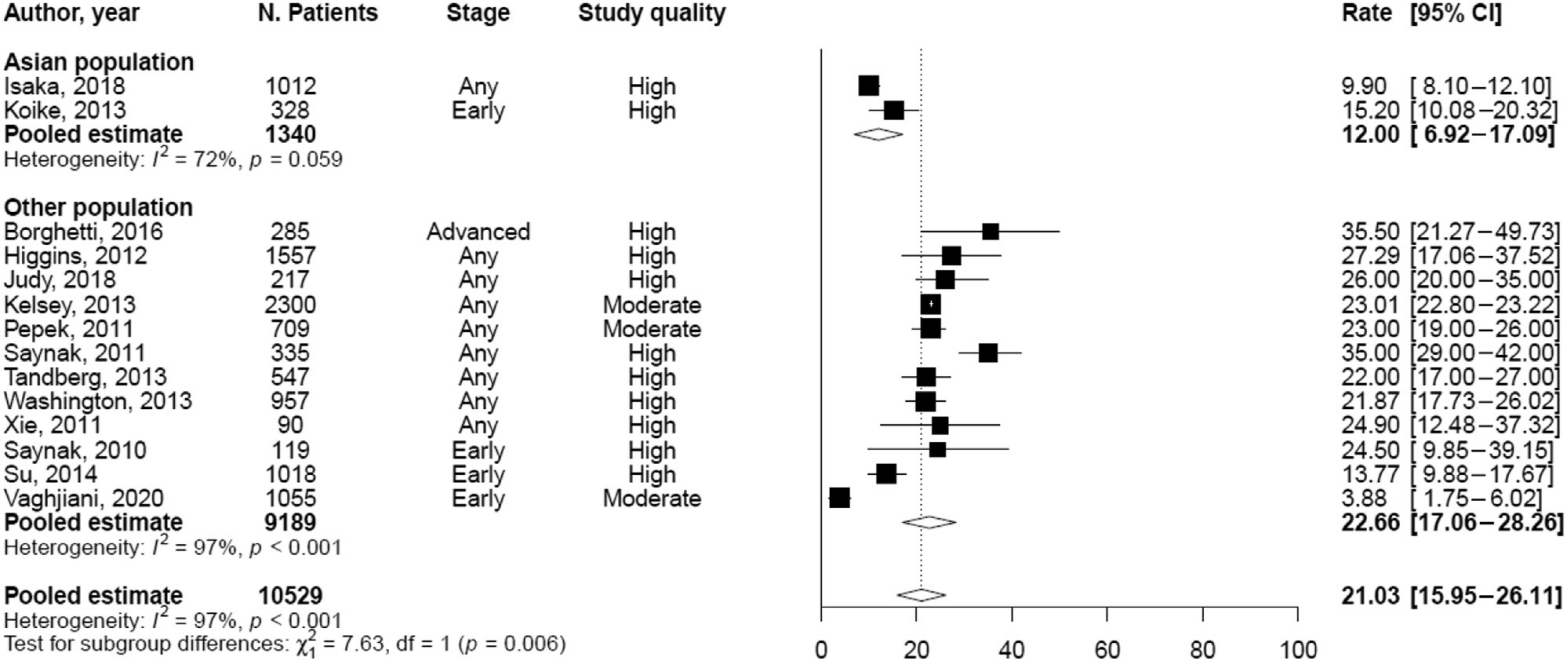
Forest plot of studies with reported 5-year local/locoregional recurrence rates in Asian and non-Asian populations after surgical resection of NSCLC. CI, confidence interval; N., number.

The pooled rate of LR at 5 years was lower in the AP (12.0%, 6.9–17.1) versus NAP (22.7%, 17.06–28.3; Fig. 1), although this was based on only two AP studies with reported 5-year LR rates. The differences between the AP and NAP populations in terms of risk factors for LR can be found in Table 3. Asian males were much more likely to have LR (1.4% RR, 1.2–1.72) versus non-Asian males (1.1% RR, 0.99–1.19, *p* = 0.003). In addition, age as a continuous variable was found to be of borderline significance for LR in the AP and significantly different as compared with the NAP. N2 versus N0 was significant only in the AP and significantly different than the NAP, but these findings are based only on one and two studies in the AP and NAP, respectively.

**Table 3:** Pooled RRs and Corresponding 95% CIs of Selected Risk Factors for Locoregional Recurrence After Surgical Resection of NSCLC Among AP and NAP

Locoregional Recurrence Risk Factor	AP		NAP		*p* Value^a^
	N. Studies	Pooled RR (95% CI)	N. Studies	Pooled RR (95% CI)	
Sex					
Male vs. female	16	1.44 (1.21–1.72)	11	1.09 (0.99–1.19)	0.003
Age					
High vs. low^b^	9	1.16 (0.91–1.48)	5	1.08 (0.76–1.55)	0.676
Continuous (1-y increase)	4	1.03 (1.00–1.06)	7	0.99 (0.98–1.01)	0.030
Tobacco smoking					
Ever vs. never	10	1.12 (0.87–1.45)	1	0.70 (0.42–1.19)	0.101
Adjuvant chemotherapy					
Yes vs. no	11	1.02 (0.66–1.56)	12	0.97 (0.73–1.28)	0.842
Type of resection					
Pneumonectomy vs. lobectomy	4	1.10 (0.62–1.94)	7	0.72 (0.50–1.04)	0.094
Segmentectomy or wedge vs. lobectomy	5	1.71 (0.54–5.34)	19	1.65 (1.31–2.09)	0.947
Sublobar NOS vs. lobar resection	1	2.24 (0.42–11.99)	6	1.83 (1.47–2.29)	0.816
Lobectomy					
VATS vs. open	5	0.86 (0.33–2.24)	8	1.14 (0.83–1.58)	0.468
Lymph node resected					
Number	1	0.98 (0.95–1.02)	4	1.00 (0.97–1.03)	0.243
Lymphovascular invasion					
Yes vs. no	16	2.26 (1.52–3.37)	16	1.64(1.38–1.95)	0.123
(Visceral) pleural invasion					
Yes vs. no	13	1.92 (1.15–3.20)	11	1.56 (1.37–1.77)	0.383
Tumor grade					
Moderate/poor vs. well	2	1.53 (0.66–3.59)	5	1.13 (0.90–1.42)	0.500
Poor vs. well/moderate	3	0.80 (0.35–1.83)	3	1.45 (1.01–2.09)	0.194
Tumor histology					
Non adenocarcinoma vs. adenocarcinoma	10	1.43 (1.10–1.86)	6	1.26 (0.91–1.75)	0.492
Non squamous cell vs. squamous cell	7	0.96 (0.74–1.25)	14	0.77 (0.64–0.93)	0.115
Tumor location					
Other location vs. right upper lobe	2	0.86 (0.55–1.35)	3	1.06 (0.75–1.50)	0.466
Left lobe vs. right lobe	5	1.08 (0.79–1.46)	1	1.16 (0.77–1.75)	0.774
Tumor size					
Large vs. small^c^	11	1.76 (1.30–2.39)	6	1.37 (1.29–1.90)	0.147
Continuous (1-cm increase)	—	—	6	1.11 (1.03–1.19)	—
Pathologic stage					
II vs. I	3	1.86 (1.04–3.31)	2	1.52 (1.34–1.73)	0.512
III–IV vs. I	2	2.20 (0.66–7.34)	2	1.49 (1.26–1.76)	0.528
Ib–II vs. Ia	3	1.19 (0.70–2.02)	3	1.45 (1.19–1.77)	0.486
III–IV^d^ vs. Ia	1	3.38 (0.36–31.56)	2	1.77 (0.76–4.11)	0.595
T stage					
T2 vs. T1	1	1.27 (0.55–2.94)	8	1.44 (1.24–1.68)	0.768
T3–T4 vs. T1	1	1.06 (0.24–4.74)	4	1.85 (1.49–2.31)	0.470
N stage					
N1 vs. N0	2	2.01 (1.25–3.25)	7	1.84 (1.37–2.46)	0.751
N2 vs. N0	1	6.85 (4.17–11.26)	2	2.03 (1.39–4.53)	0.012
N1–N2 vs. N0	4	1.65 (0.93–2.94)	8	1.64 (1.31–2.05)	0.976

AP, Asian population; CI, confidence interval; N., number; NAP, non-Asian population; NOS, not otherwise specified; RR, relative risk; VATS, video-assisted thoracoscopic surgery.

a *p* value for heterogeneity across AP and NAP.

b Publications reporting age categories below 65 to 75 years or above 65 to 75 years.

c Publications reporting tumor size categories less than 1.5 to 4 cm or more than 1.5 to 4 cm.

d It should be noted that one of 548 patients and two of 261 patients in the manuscripts by Kim et al.^14^and Chen et al.^15^ were noted to be stage 4 patients, in which tumors in separate ipsilateral lobe are now classified as T4 tumors.

**Figure 2:**
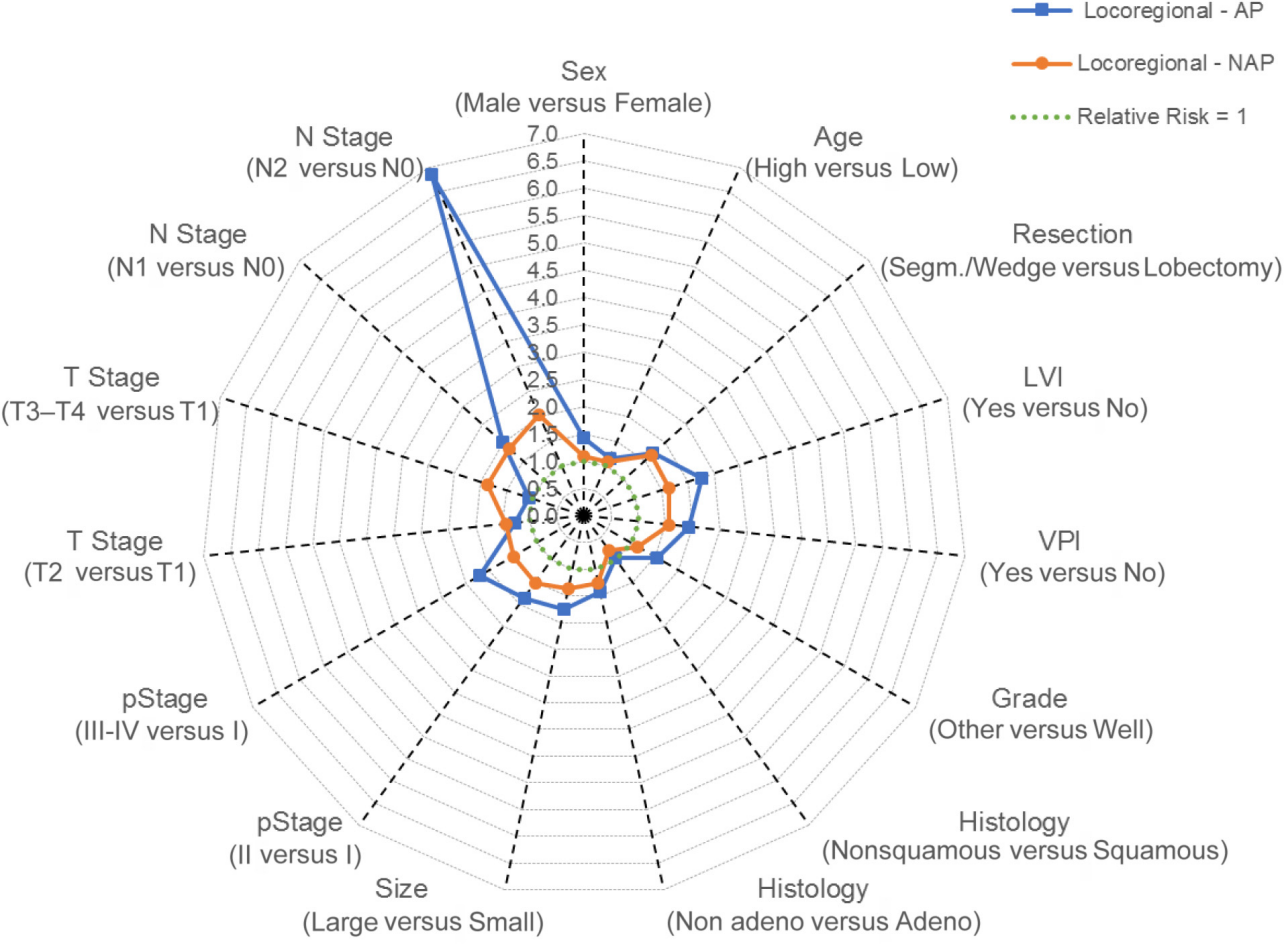
Radar plot of risk factors and their relative risk of local recurrence in AP and NAP. Adeno, adenocarcinoma; AP, Asian population; LVI, lymphovascular invasion; NAP, non-Asian population; pStage, pathologic stage; VPI, visceral pleural invasion.

Figure 2 illustrates the risk factors and their associated RR for LR on a radar plot.

The pooled rate of DR at 5 years was 22.4% (16.02–30.72) in the NAP, whereas none of the AP publications reported data on pooled DR (data not found). Supplementary Table 7 reveals the risk factor difference between the AP and NAP for DR. Ever smoking was only of borderline significance for DR in the NAP and was significantly different than the AP, but there was only one study available in the NAP for comparison. LVI was significantly associated with DR in both populations, but the RR was significantly higher in the AP than in the NAP (3.41, 2.39–4.87 versus, 1.47, 1.20–1.79, *p* < 0.001). Stage had mostly remained significant for DR in both populations, but in stages Ib to II versus Ia, the RR was higher in the NAP than in the AP (2.53, 1.74–3.67, versus 1.64, 1.31–2.05, *p* = 0.049).

The 5-year OvRec was similar in both populations: 38.0% (25.2–50.9) in AP and 37.3% (32.4–42.2) in NAP (data not found). The difference between both populations for OvRec factors is displayed in Supplementary Table 8. Age as a continuous variable in relation to OvRec was significantly different in AP and NAP (1.02, 1.00–1.04, versus 1.00, 0.98–1.01, *p* = 0.024). Ever smoking was only significant for OvRec in the AP and significantly different than the NAP (1.30, 1.04–1.62, versus 0.73, 0.40–1.32, *p* < 0.001). LVI was significant in both populations for OvRec, but significantly more so in the AP (2.02, 1.64–2.51, versus 1.39, 1.24–1.56, *p* = 0.002). Moderate/poor tumor grade was significantly associated with DR in both populations, but it was significantly greater in the AP than the NAP (4.72, 2.28–9.77, versus 1.65, 1.21–2.25, *p* = 0.008). All cases of node involvement were significantly associated with OvRec in both populations, but more so in the AP than NAP for all categories, respectively, as follows: N1 versus N0 2.05 (1.58–2.67) versus 1.54 (1.49–1.60, *p* = 0.034), N2 versus N0 2.58 (2.24–3.63) versus 1.74 (1.66–1.84, *p* < 0.001), and N1 to N2 2.71 (2.37–3.10) versus 1.86 (1.44–2.40, *p* = 0.010).

A lower 5-year mortality rate was noted in AP (24.3%, 15.6–33.0) than in NAP (45.9%, 41.2–50.5). The OS was significantly affected by LVI and moderate/poor differentiation, but the effect was significantly greater in the AP than the NAP (LVI: 1.82, 1.34–1.64, versus 1.34, 1.24–1.45, *p* = 0.014; moderate/poor differentiation: 3.98, 2.13–7.44, versus 1.46, 1.21–1.76, *p* = 0.003) (Supplementary Table 9). Nonadenocarcinoma versus adenocarcinoma was significant for OS in both populations, but the extent of the effect was significantly greater in the AP than in the NAP (1.69, 1.13–2.54, versus 1.06, 0.91–1.23, *p* = 0.033). Similarly, the effects of N1 and N2 on OS were significant in both populations, but more so in the AP than in the NAP (N1 versus N0 2.31, 1.67–3.19, versus 1.48, 1.43–1.54, *p* = 0.008; N2 versus N0 2.87, 2.12–3.87, versus 1.86, 1.78–1.96, *p* = 0.006).

## Discussion

We decided to perform this meta-analysis to clarify the risk factors for LR because of differences in the LR noted in both control arms of the recently published Lung ART and PORT-C trials in European and Chinese populations, respectively.^10^^,^^11^ In part, the motivation for this stemmed from the recognition that in spite of the fact that postoperative thoracic radiotherapy improves local control, an OS benefit remains elusive, in significant part owing to cardiopulmonary toxicities. Furthermore, the recognition that *EGFR* mutations occur far more often in nonsmoking Asian females and the natural history of this entity is unique, we surmised that these and other differences could drive differences in local relapse and the underlying risk factors. Although it is difficult to compare different trials, it seems that LR was higher in the control arm of the Lung ART trial, which noted that 46% of recurrences were in the mediastinum.^10^ The crude rate of mediastinal recurrence in all patients was 28.1% in this trial. We feel that this rate may be higher if actuarial or Kaplan-Meier rates were calculated. Despite not providing the precise definition of LR in the manuscript or in the actual protocol, the 3-year local recurrence rate in the control arm of the PORT-C trial was 18.3%.^11^ Because we would assume that the definition of LR in this trial would at least include the mediastinum, ipsilateral hilum, and ipsilateral lung, we believe that the difference between these two trials would be magnified if recurrences were just restricted to the mediastinum. Nevertheless, it should be noted that the nature of NSCLC is different in AP than NAP, largely because of differences in *EGFR* mutation (EGFRmut) frequency, which were found to be present in 47% (range 20%–76%) of adenocarcinomas in the AP as compared with 15%^4^^,^^6^, ^7^, ^8^, ^9^, ^10^, ^11^, ^12^, ^13^, ^14^, ^15^, ^16^, ^17^, ^18^, ^19^, ^20^, ^21^, ^22^, ^23^, ^24^, ^25^, ^26^, ^27^, ^28^, ^29^, ^30^, ^31^, ^32^, ^33^, ^34^, ^35^, ^36^, ^37^, ^38^, ^39^, ^40^ and 22%^3^, ^4^, ^5^, ^6^, ^7^, ^8^, ^9^, ^10^, ^11^, ^12^, ^13^, ^14^, ^15^, ^16^, ^17^, ^18^, ^19^, ^20^, ^21^, ^22^, ^23^, ^24^, ^25^, ^26^, ^27^, ^28^, ^29^, ^30^, ^31^, ^32^, ^33^, ^34^, ^35^, ^36^, ^37^, ^38^, ^39^, ^40^, ^41^ of the European and North American populations, respectively.^21^ Not only does this difference between the trials suggest that there may be differences in LR between the AP and NAP, but it raises the question of whether N2 is the best risk factor to be used for predicting the risk of LR. It is likely that both trials were based on the PORT meta-analysis, which suggested a benefit from PORT in patients with resected N2 NSCLC.^7^ Nevertheless, if one looks closely at this PORT meta-analysis, the HR for OS reveals that PORT is increasingly detrimental for lower stages and lower nodal stages. The studies contained in the PORT meta-analysis included patients who were treated between 1965 and 1995. Because almost all radiation therapies during this time period were two-dimensional radiation, another way of interpreting the PORT-meta-analysis is that the competing risks of radiation-related mortality using antiquated techniques became increasingly less as the stage and nodal stage increased because these patients were more likely to die from lung cancer. Therefore, because stage I and stage N0 patients were likely to live longer from NSCLC, they were more at risk for iatrogenic deaths.

The main risk factors for LR in our meta-analysis were N2, LVI, and advanced T stage (T3–T4). When including only HQ studies by the NOSc, the highest RRs for LR were LVI, sublobar resection, and N1, but N2 was no longer significant, with the caveat that this conclusion is based only on two studies. The present meta-analysis thus reveals the limited information in the clinical literature pertaining to nodal involvement and its relationship to recurrence patterns. Only three and nine studies of 87 eligible studies had information pertaining to N2 and N1 nodal disease, respectively, which fell to two and eight studies when considering HQ studies. Although the RR for N2 disease remained high for LR using HQ studies, it should be noted that when combining N2 with N1 disease as compared with N0 nodal status, RRs for LR were lower in all combined studies (moderate quality and HQ) and in the HQ study population as compared with the RRs for N1 nodal status versus N0 (all studies: N1 versus N0 1.85, 1.45–2.35, and N1 to N2 versus N0 1.63, 1.30–2.04; HQ studies: N1 versus N0 1.84, 1.37–2.47 and N1–N2 versus N0 1.56, 1.20–2.03). We speculate based on these data that perhaps with a larger number of studies, N2 nodal status may not be as important for LR as N1 nodal status. One previous study hypothesized that N1 rather than N2 nodal involvement may be more likely to result in LR because N1 disease is less likely to fail distally.^9^ Interestingly and paradoxically to the LR rates in the Lung ART and PORT-C trials, there was no relationship between nodal status and LR in HQ studies from the United States,^9^ whereas there was a relationship in HQ studies from AP,^8^ despite higher and lower rates of LR in the Lung ART and PORT-C control arms. Noteworthy is the significant effect of LVI on LR, despite there being known inter-reader variability in the diagnosis of this pathologic finding between facility type/location and surrounding population size.^22^

Although the RR for LRs in patients with pN2 disease in the AP (pN2 versus pN0, RR = 6.85) is much higher than that in the NAP (pN2 versus pN0, RR = 2.03), this is a RR and not absolute risk for LR. This effect is largely because of the low rate of LR in the AP patients with resected N0 disease. In fact, the rate of LRs at 5 years in patients with pN0 disease was higher in NAP patients (14.9%)^9^ than in AP patients (3.9%),^8^ in the HQ N0Sc studies used in our meta-analysis. Likewise, in our analysis of all patients, the pooled rate of LR at 5 years was lower in the AP (12.0%, 6.9%–17.1%) than the NAP (22.7%, 17.06%–28.3%) considering only HQ studies.

It is interesting to note that adjuvant chemotherapy had no effect on LR in the total population nor in the HQ study subset. Nevertheless, the influence of systemic therapy on recurrence patterns may change now that adjuvant chemo/immunotherapy in programmed death-ligand 1 (PD-L1)–positive NSCLC,^23^ adjuvant osimertinib in EGFRmut NSCLC,^24^ and neoadjuvant chemo/immunotherapy^25^ have received Food and Drug Administration approval based on their DFS benefits. The IMpower 010 randomized trial primary end point of increasing DFS was achieved using adjuvant atezolizumab versus placebo after adjuvant chemotherapy in stages II to IIIA, at PD-L1 greater than 1%. Nevertheless, in subgroup analysis, the DFS benefit was largely limited to the PD-L1 greater than 50% tumors and did not seem to benefit node-negative disease.^23^ Although disease relapse was higher in the placebo arm in the entire population (stage IB [tumors ≥ 4 cm] to IIIA tumors regardless of PD-L1 expression), there was no difference in the patterns of relapse between the placebo and the adjuvant atezolizumab arms with LR-only relapse occurring at 37.8% and 36.9% in the atezolizumab and placebo arms, respectively.^26^ In EGFR-mutant NSCLC, 3 years of adjuvant osimertinib plus or minus chemotherapy versus placebo had a profound effect on DFS (HR = 0.17) with low comparative LR rates, at 11% versus 46%. Nevertheless, whether the effects of osimertinib on DFS will diminish after the medication is stopped, as noted in the adjuvant experience with first-generation tyrosine kinase inhibitors^27^ and whether it is more cost-effective or safer to consider local therapy in those at risk for LR to decrease the duration of osimertinib or prevent relapse after the medication cessation are not known. Moreover, at the European Society for Medical Oncology 2022, with prolonged follow-up, it was noted that recurrences did indeed increase after the 3-year treatment period with osimertinib.^28^ It should be noted that a large retrospective series revealed that there is no real difference between OvRec based on whether or not the patients had an EGFRmut or EGFR wild-type tumor and that LR rates were similar among tumors with EGFR mutated (16.5%) and EGFR wild-type (16.8%) tumors in a preadjuvant osimertinib, but recent patient cohort.^29^ Neoadjuvant chemo/immunotherapy has resulted in impressive complete response rates of 18% to 63%, and a DFS benefit as compared with neoadjuvant platinum doublet chemotherapy alone in CheckMate 816, but whether the long-term effect of this approach will be associated with improved OS or reduced LR is currently unknown.^30^

Considering only HQ studies, it is not surprising that sublobar resection, whether a known wedge resection/segmentectomy or a sublobar resection NOS, was associated with a statistically significant risk for LR. Both published prospective, randomized trials^4^^,^^31^ evaluating lobectomy versus sublobar resection have revealed a significantly higher LR in the sublobar arm despite including patients with relatively low risk tumors with the Japan Clinical Oncology Group 0802 study including only tumors less than or equal to 2 cm with a consolidation/tumor ratio greater than 0.5 and the Lung Cancer Study Group only including tumor less than or equal to 3 cm. Nevertheless, unlike the Lung Cancer Study Group, the Japan Clinical Oncology Group 0802 met its primary end point and found an improved OS (94.3% versus 91.1%), largely because of a lower percentage of deaths stemming from other malignancies (including second lung cancers), respiratory diseases, and cerebrovascular accidents. One can speculate that the greater preservation of pulmonary tissue in the segmentectomy arm allowed for more aggressive treatment of subsequent second lung cancers/lung cancer recurrences and decreased the risk of patients succumbing to respiratory dysfunction more likely to occur after a lobectomy. The Cancer and Leukemia Group B 140503 randomized patients to lobectomy or sublobar resection for small (≤2 cm) peripheral NSCLC, but it closed after only accruing 701 of 1258 patients.^32^ It should be noted that none of the trials of sublobar resection found an improvement in pulmonary function^4^^,^^31^ or perioperative mortality/morbidity.^31^^,^^33^ Cancer and Leukemia Group B 140503 was recently published. This multi-institutional North American trial randomized patients to lobectomy or sublobar resection for T1aN0M0 (≤2 cm) peripheral (outer third of the lung) NSCLCs and reached its primary end point and secondary end points of noninferiority of sublobar resection in both DFS and OS, respectively. There was no substantial difference between the surgical groups with respect to LR or DR. Importantly, all patients had to have pathologically confirmed NSCLC and node-negative disease at level 10 and up to two mediastinal stations before randomization.^34^

The pooled 5-year rate of LR was lower in the AP versus NAP, although this is based on only two AP studies reporting 5-year LR rates. The RRs for LR were consistent for most factors in AP and NAP, although the RR for male versus female sex was higher in AP than in NAP. Risk factors for LR noted in both populations are LVI, VPI, nonadenocarcinoma, and tumor size with all risk factors associated with a greater RR on LR in the AP. Although the staging variables had general trends toward an increased risk of LR, each factor is associated with a scanty number of articles in both populations except the node-positive category (N1–N2 versus N0) which reveals that the effect in the AP is not significant (1.65, 0.93–2.94). Because factors such as LVI (2.26, 1.52–3.37), VPI (1.92, 1.15–3.20), and tumor size (1.76, 1.30–2.39) were associated with a higher and significant RR for LR in the AP, this may indicate why the PORT-C trial, which randomized to PORT or not without stratification factors, represents an underpowered positive trial for the beneficial effects of PORT. The effect of nodal involvement in the NAP is not only significant (1.64, 1.31–2.05), but, with the exception of T3 to T4 tumors, the RR is much higher than large tumor size (1.37, 1.29–1.90), poor differentiation (1.45, 1.01–2.09), or pleural invasion (1.56, 1.37–1.77), and possibly explains why the mediastinal failure rate of 28.1% was much higher in the Lung ART trial.

Although similar 5-year OvRec rates were noted in both the AP and NAP, survival was lower in the NAP. Of note, most factors that were significantly associated with both DR and OvRec in both populations such as tumor size, LVI, VPI, and poor differentiation are considered high-risk factors warranting adjuvant systemic therapy in node-negative resected NSCLC^35^ whereas most of these factors are associated with LR in both populations but are not considered risk factors for the recommendation of local therapy such as radiation. Variables associated with OS in our meta-analysis have been found in many studies previously,^36^, ^37^, ^38^, ^39^ but LVI, tumor grade, nonadenocarcinoma histology, and N1/N2 stage had a significantly higher RR on OS in the AP. Despite similar OvRec rates, the AP had a prolonged survival likely due to the high incidence of EGFRmut adenocarcinoma and because of the efficacy of first-generation^40^ and third-generation agents^41^ for this mutation during the years of the studies included in this investigation.

One can question the validity of the present meta-analysis because it includes all eligible surgical series irrespective of the surgical quality. For example, one recent investigation assessed eight sequentially more stringent definitions of pathologic node evaluation and revealed that the 5-year survival was sequentially improved for N0 and N1 tumors. In addition, this study noted that the more stringent definitions of mediastinal node examination were associated with a larger separation of survival curves between N1 and N2 tumors.^42^ Nevertheless, the Alliance for Clinical Trials in Oncology Z00300 trial randomized patients to mediastinal node sampling versus complete dissection in N0 or N1 (nonhilar) tumors and noted no difference in OS (primary end point) or secondary end points (LR, regional recurrence, or DR).^43^ Extent of complete resection has been found to be a risk factor for recurrence, but studies have used varying definitions and required differing levels of adherence for patient inclusion.^5^^,^^10^^,^^44^, ^45^, ^46^, ^47^

It should be noted that this meta-analysis, although thorough and comprehensive, is limited by the methods of detection/surveillance and retrospective design of many included studies. The definition of LR differed between studies and there was no way ex post facto for controlling for the differences. This definition even differs between large surgical groups.^48^^,^^49^ To detect how the different definitions of LR could have biased our results, we grouped the studies into four categories by definition of LR (most inclusive, less inclusive, least inclusion, and unknown; Supplementary Table 4). We correlated the weighted percentage of LR and DR by the LR definition and by the previously mentioned staging subgroups (first paragraph of results section)—early stage (pT1–T2 N0 tumors), advanced stage (patients with only node-positive disease), and any stage (all surgical stages I–III). As can be found in Supplementary Tables 10 to 13 and Supplementary Figures 5 to 8, LR and DR correlated with staging subgroups, but not LR definition. So the different definitions of LR have minimal impact on our study results. We also acknowledge that we separated the NAP and AP populations by reporting country and that there may have been some patients of Asian origin in the U.S. population, but one recent Surveillance, Epidemiology, and End Results analysis revealed that only 5.58% of surgically resected patients with lung cancer were of Asian descent.^50^ Furthermore, none of the included investigators used molecular tissue diagnostics to determine somatic driver mutations, such as EGFRmut, ALK fusion oncogenes, c-ROS oncogene 1, BRAF 600E, KRAS G12C, MET exon 14 skipping mutations, RET fusion mutations, and HER-2 exon 20 mutations. The long-term impact of these mutations and their evolving therapies on recurrence patterns after surgery will need to be elucidated. In addition, the included investigation did not use circulating tumor DNA (ctDNA) for the detection of postsurgical recurrences. Small postsurgical series have noted that ctDNA was not only able to detect minimal residual disease months before radiographic failures but also able to find whether chemotherapy was effectively treating the molecularly detected disease.^51^, ^52^, ^53^ Nevertheless, one investigation noted that only 18% of patients were found to have a detectable mutation by means of ctDNA before surgery.^53^ Perhaps the promising technology of ctDNA will be used in conjunction with our existing histopathologic variables to help improve our ability to detect postsurgical recurrences and their associated failure patterns.

A major impetus for performing this research study was to inform lung cancer researchers that before initiation of the PORT-C^11^ and Lung ART^10^ trials, there was insufficient evidence for revealing pN2 nodal disease is a risk factor for LR, and thus the basis for these trials was flawed. In addition, our meta-analysis has found that there is still scant proof that pN2 disease is related to LR. We feel that we have found that there is insufficient available information concerning pN2 as a risk factor for LR. Furthermore, by revealing LR RRs are lower for pN1 to N2 versus pN0 than for pN1 versus pN0, we strongly suggest that pN1 disease may be a higher risk factor for LR than pN2 disease. Another major aim was to understand why LR seemed to be much lower in the AP in the PORT-C trial than the NAP in the Lung ART trial despite the restrictive definition of LR in the Lung ART trial. Although both trials are similar because only pN2 disease and complete resection (margin negative) were needed for eligibility, there were some fundamental differences that could have affected the recurrence rates. The Lung ART included more patients with gross nodal involvement (clinical N [cN]2) which we would assume is related to LR. In the Lung ART trial, 58% to 59% of patients had cN2 disease, which was well-balanced between study arms, but there was a lower percentage of patients in the PORT-C trial with cN2 disease, and cN2 was higher in the radiation arm (43.5% versus 35.6%). In the observation arm of the PORT-C trial, 5.6% of the patients received PORT. Furthermore, the PORT-C trial required that all patients receive four cycles of platinum doublet chemotherapy as eligibility criteria, whereas the Lung ART did not. Despite not requiring chemotherapy, 96% of patients in the Lung ART trial received either preoperative, postoperative chemotherapy, or both. Nevertheless, because post hoc analyses of two randomized trials^3^^,^^54^ have suggested that chemotherapy has an effect on LR, and chemotherapy was not protocol controlled in the Lung ART trial, this less controlled use of systemic therapy may have played a difference in the LR rates. Because most of the data in this meta-analysis were obtained long after the proven benefit of chemotherapy with the publication of the IALT trial in 2004,^55^ we feel that the lack of effect of chemotherapy on LR in this analysis could have been due to patient selection bias for chemotherapy in the included studies.

In conclusion, we revealed that the AP may be at a lower risk of local recurrence compared with NAP and that N2 nodal disease may not be the best and only determinant for risk of locoregional failure in surgically resected NSCLC. The lower risk of LR in the AP may have led to an underpowered trial, the phase 3 PORT-C trial, to determine the impact of radiation in the N2 population. Now that we have found that PORT can be given with no therapy-related grade 4 or 5 toxicity^11^ by means of IMRT, the determination of the population at risk for LR will be quite important. We feel that our meta-analysis summarizes the patterns of recurrence in the surgical populations in the premolecular diagnostic era.

## CRediT Authorship Contribution Statement

**John M. Varlotto:** Conceptualization, Investigation, Methodology, Writing (original draft, reviewing, editing).

**Cristina Bosetti:** Data curation, Formal analysis, Investigation, Methodology, Writing (original draft, reviewing, editing).

**Dwight Bronson:** Conceptualization, Funding Acquisition, Investigation, Methodology, Writing (original draft, reviewing, editing).

**Claudia Santucci:** Data curation, Formal analysis, Investigation, Methodology, Writing (original draft, reviewing, editing).

**Maria Vitttoria Chiaruttini:** Data curation, Formal analysis, Investigation, Methodology, Writing (original draft, reviewing, editing).

**Marco Scardapane:** Investigation, Methodology, Writing (original draft, reviewing, editing).

**Minesh Mehta:** Investigation, Methodology, Writing (original draft, reviewing, editing).

**David Harpole:** Investigation, Methodology, Writing (original draft, reviewing, editing).

**Raymond Osarogiagbon:** Investigation, Methodology, Writing (original draft, reviewing, editing).

**Gerald Hodgkinson:** Conceptualization, Funding Acquisition, Investigation, Methodology, Writing (original draft, reviewing, editing).
