# Supplementary material for: Meta-Analysis of Rates and Risk Factors for Local Recurrence in Surgically Resected Patients With NSCLC and Differences Between Asian and Non-Asian Populations
Source: JTO Clin Res Rep. 2023 Apr 6;4(10):100515. doi: 10.1016/j.jtocrr.2023.100515 (PMC10518711; doi:10.1016/j.jtocrr.2023.100515)

**SUPPLEMENTARY MATERIAL**

**Supplementary Table 1.** Strings used for the literature search in Pubmed/MEDLINE.

| **N. records** | **Search terms** | **N. records** |
| --- | --- | --- |
| #1 | ("non small cell"[Title/Abstract] OR nonsmallcell[Title/Abstract] OR "nonsmall cell"[Title/Abstract] OR "non smallcell"[Title/Abstract] OR NSCLC[Title/Abstract]) AND (lung[Title/Abstract]) AND (cancer[Title/Abstract] OR carcinoma[Title/Abstract] OR Adenocarcinoma[Title/Abstract] OR "Adeno carcinoma"[Title/Abstract] OR "Squamous cell carcinoma"[Title/Abstract] OR "Squamous Carcinoma"[Title/Abstract] OR Adenosquamous[Title/Abstract] OR Tumor*[Title/Abstract] OR Tumor*[Title/Abstract]) | 76,833 |
| #2 | **(Lobectom*[Title/Abstract] OR Segmentectom*[Title/Abstract] OR Penumectom*[Title/Abstract] OR Resect*[Title/Abstract] OR Surg*[Title/Abstract] OR Operat*[Title/Abstract] OR Post Operat*[Title/Abstract] OR Postoperat*[Title/Abstract] OR Post-operat*[Title/Abstract] OR Excis*[Title/Abstract])** | 3,274,219 |
| #3 | ((Local*[Title/Abstract] OR Locoregion*[Title/Abstract] OR “Loco region*” [Title/Abstract] OR Distant[Title/Abstract] OR overall[Title/Abstract]) AND (Recur*[Title/Abstract] OR Fail*[Title/Abstract] OR Relaps*[Title/Abstract] OR progression*[Title/Abstract])) | 221,577 |
| #4 | Risk*[Title/Abstract] OR Factor*[Title/Abstract] OR Prognos*[Title/Abstract] OR Predict*[Title/Abstract] OR Rate*[Title/Abstract] | 8,651,384 |
| #5 | stage[Title/Abstract] OR Grade[Title/Abstract] OR histol*[Title/Abstract] OR “Tumor Size*”[Title/Abstract] OR “Cell Type*”[Title/Abstract] OR Invas*[Title/Abstract] OR resect*[Title/Abstract] OR surg*[Title/Abstract] OR location*[Title/Abstract] OR chemot*[Title/Abstract] OR PET[Title/Abstract] OR classific*[Title/Abstract] OR Lymph*[Title/Abstract] OR LVI[Title/Abstract] OR Diabetes[Title/Abstract] OR Body mass index[Title/Abstract] OR CEA[Title/Abstract] OR Carcinoembryon*[Title/Abstract] OR “Carcino embryon*”[Title/Abstract] | 6,415,277 |
| #1-#5 |  | 2053 |
| #1-#5 | Limit from 2000 | 1755 |
| #1-#5 | Limit to English | 1633 |

**Supplementary Table 2**. Studies excluded from the meta-analysis and reason for exclusion.

| **Study reference** | **Reason for exclusion** |
| --- | --- |
| Cheng, 2012 [1] | Only forced expiratory volume |
| Li, 2015 [2] | Only blood type |
| Maurizi, 2015 [3] | Only margin distance |
| Mazza, 2015 [4] | Only pleural lavage cytology |
| Mohiuddin, 2014 [5] | Only margin distance |
| Okada, 2006 [6] | Only selective vs complete dissection |
| Shimada, 2010 [7] | Only intratumoral vs extratumoral vascular invasion |
| Takahashi, 2015 [8] | Only neutrophil-to-lymphocyte ratio |
| Takeda, 2005 [9] | Only mediastinal lymph node dissection vs mediastinal lymph node sampling |
| Varlotto, 2010 [10] | Same cohort as in [11] |
| Xia, 2016 [12] | Only lymph node ratio |
| Corsini, 2021 [13] | Only major pathological response |
| Schreiner, 2020 [14] | Only major pathological response |

**Supplementary Table 3.** Main characteristics of the studies on risk factors for locoregional recurrence after surgical resection of non-small cell lung cancer (NSCLC) included in the meta-analysis.

| **Study reference** | **Country** | **Study period** | **N. patients** | **Population type, TNM edition** | **Population classification^a^** | **Resection type** | **Follow-up (months)** |
| --- | --- | --- | --- | --- | --- | --- | --- |
| Okada, 1999 [15] | Japan | 1984-1998 | 120 | pStage T1-T4, N0-N2; TNM V | Any stage | Sleeve lobectomy or  pneumonectomy | 69 (9-174) |
| Mineo, 2001 [16] | Italy | 1988-1994 | 66 | Stage IB; TNM V | Early stage | Pneumonectomy, lobectomy | 60 for all patients |
| Maruyama, 2004 [17] | Japan | 1992-1998 | 143 | Peripheral NSCLC; TNM V | Any stage | Lobectomy, bilobectomy, pneumonectomy, or segmentectomy | - |
| Lardinois, 2005 [18] | Switzerland | 1995-1996 | 92 | Stage I-IIIA, N0-1; TNM - | Any stage | Lobectomy, pneumonectomy | 88.9 ± 6.6 |
| El-Sherif, 2006 [19] | USA | 1990-2003 | 784 | Stage I; TNM VI | Early stage | Sublobar resection, lobectomy | 31 (only for patients remaining alive) |
| Fujimoto, 2006 [20] | USA | 1997-2002 | 180 | T1-4 N1 M0; TNM V | Advanced stage | Lobectomy, sleeve lobectomy, bilobectomy, or pneumonectomy | 33 (0.1-92.4) |
| Shiraishi, 2006 [21] | Japan | 1994-2005 | 160 | Stage IA; TNM - | Early stage | Lobectomy | 43.6 ± 27.5 (VATS)/48.1 ± 32.3 (Open) |
| Schuchert, 2007 [22] | USA | 2002-2006 | 428 | Stage IA-IB; TNM VI | Early stage | Segmentectomy or lobectomy | 18.1 (Segmentectomy)/28.5 (Lobectomy) |
| Sienel, 2007 [23] | Germany | 1987-2002 | 199 | pT1pN0cM0; TNM V | Early stage | Segmentectomy/lobectomy, systematic lymph node dissection | 54 (3-192) |
| Tsuchiya, 2007 [24] | Japan | 1978-2005 | 322 | Stage IA; TNM V | Early stage | Lobectomy, pneumonectomy Wedge/Segmentectomy | - |
| Shiraishi, 2008 [25] | Japan | 1994-2005 | 75 | Stage I, N1-N2; TNM - | Advanced stage | Lobectomy, systemic lymph node dissection | 40 ± 25.7 (VATS)/34.3 ± 29.7 (Open) |
| Sienel, 2008 [26] | Belgium | 1987-2003 | 87 | Stage IA; TNM VI | Early stage | Segmentectomy, wedge resection | 45 (3-192) |
| Voltolini, 2008 [27] | Italy | 1994-2000 | 179 | Peripheral c-Ia; TNM VI | Early stage | Lobectomy, bilobectomy, sleeve lobectomy, pneumonectomy | 48 (2-108) |
| Bando, 2009 [28] | Japan | 1993-2002 | 91 | T1N0M0; TNM V | Early stage | Segmentectomy | 72 |
| De Giacomo, 2009 [29] | Italy | 1999-2007 | 152 | Stage I; TNM V | Early stage | Lobectomy/Sublobar resection | 59 |
| Schuchert, 2009 [30] | USA | 2002-2007 | 225 | Stage IA,IB; TNM VI | Early stage | Segmentectomy | 16.2 (VATS)/28.2 (Open) |
| Shapiro, 2009 [31] | USA | 2002-2008 | 144 | Stage I; TNM VI | Early stage | Segmentectomy, lobectomy | 22 ± 16.6 (Segmentectomy)/21 ± 12.6 (Lobectomy) |
| Varlotto, 2009 [11] | USA | 2000-2005 | 373 | Stage I-III; TNM - | Any stage | Any | 33 (4-98) |
| Kim, 2010 [32]^b^ | Korea | 2003-2008 | 548 | Stage I-IV; TNM - | Any stage | VATS lobectomy, thoracotomy | 20 |
| Okami, 2010 [33] | Japan | 1990-2007 | 764 | Stage IA; TNM VI | Early stage | Lobectomy, sublobar resection | - |
| Saynak, 2010 [34] | USA | 1996-2006 | 119 | Stage IA; TNM - | Early stage | Lobectomy, bilobectomy, pneumonectomy | 47 (1-121) |
| Shoji, 2010 [35] | Japan | 1991-2003 | 217 | Stage IA; TNM VII | Early stage | Lobectomy, limited resection | 59 (0-170) |
| Darling, 2011 [36]^c^ | USA | 1999-2004 | 1023 | N0 or non-hilar N1, T1 or T2; TNM V | Early stage | Any | 78 |
| Koo, 2011 [37] | Korea | 2002-2004 | 310 | Stage I-II; TNM VII | Any stage | Any | 60 (1.3-90.4) |
| Park, 2011 [38] | Korea | 2003-2007 | 529 | Stage I; TNM VI | Early stage | Lobectomy | 24.6 ± 12.1 (VATS)/30.9 ± 13.4 (Open) |
| Pepek, 2011 [39] | USA | 1995-2005 | 709 | N0-N2; TNM VI-VII | Any stage | Lobectomy, pneumonectomy | 32 (1-174) |
| Saynak, 2011 [40] | USA | 1996-2006 | 335 | T1–4 N0–1; TNM VII | Any stage | Lobectomy, pneumonectomy | 31 (1-50) |
| Schuchert, 2011 [41] | USA | 2002-2007 | 524 | Stage I; TNM VII | Early stage | Segmentectomy, lobectomy | 35.6 (0.2-85.2) |
| Varlotto, 2011 [42] | USA | 2000-2006 | 60 | N1; TNM - | Advanced stage | Wedge resection, segmentectomy, lobectomy/bilobectomy, pneumonectomy | 30 (3-96) |
| Wolf, 2011 [43] | USA | 2000-2005 | 468 | Small tumor (<=2cm); TNM VI | Early stage | Lobectomy or sublobar | - |
| Xie, 2011 [44] | USA | 2000-2007 | 90 | Stage T1–3N0–1; TNM VII | Any stage | Lobectomy, pneumonectomy | 54.3 (1-99) |
| Carr, 2012 [45] | USA | 2002-2009 | 429 | Stage IA; TNM VII | Early stage | Segmentectomy, lobectomy | 45.3 |
| Higgins, 2012 [46] | USA | 1995-2008 | 1559 | pT1-3N0-2; TNM VII | Any stage | Any | 34 |
| Higgins, 2012 [47] | USA | 1995-2008 | 198 | T1-3N1M0; TNM VII | Advanced stage | VATS or Open lobectomy | 24 |
| Hung, 2012 [48] | Taiwan | 1980-2000 | 756 | Stage I; TNM VI-VII | Early stage | Complete resection with lymph nodes dissection | 67.7 |
| Hung, 2012 [49] | Taiwan | 1990-2006 | 355 | Node-negative NSCLC; TNM VII | Early stage | Complete resection,mediastinal lymph nodes dissection/sampling | 54.2 |
| Stefani, 2012 [50] | Italy | 1996-2010 | 206 | Small tumor (<= 2 cm) T1N0; TNM - | Early stage | Wedge resection, lobectomy | 49 (3.1-179) |
| Varlotto, 2012 [51] | USA | 2000-2006 | 189 | Stages I–IIIA; TNM - | Any stage | Any | 33 (3-98) |
| Choi, 2013 [52] | Korea | 1995-2012 | 242 | Stage I; TNM VI | Early stage | Lobectomy, lymph nodes dissection | 44.3 |
| Fan, 2013 [53] | China | 2007-2008 | 199 | T1-3N1M0; TNM VII | Advanced stage | Any | 53.8 (1.4-81.8) |
| Kelsey, 2013 [54] | UK, USA | 1995-2008 | 1411/889 | Stage I-III A; TNM VII | Any stage | Any | - |
| Koike, 2013 [55] | Japan | 1998-2009 | 328 | Stage IA; TNM VII | Early stage | Any | 58 (2-162) |
| Lopez Guerra, 2013 [56] | USA | 1998-2009 | 1402 | Stage I–III, N0–N1; TNM VI | Any stage | Complete resection | 42 (1-156) |
| Tandberg, 2013 [57] | UK | 1995-2008 | 547 | pN0-1; TNM VII | Any stage | Any | 29 (1-170) |
| Varlotto, 2013 [58] | USA | 2000-2006 | 411 | Stage I; TNM - | Early stage | Sublobar resection, lobectomy | 34 (3-98) |
| Washington, 2013 [59] | USA | 1995-2005 | 957 | Stage I-IIIA; TNM VII | Any stage | Any | 30 (1-149) |
| Yanagawa, 2013 [60] | Japan | 1995-2010 | 433 | Stage I; TNM VII | Early stage | Lobectomy, segmentectomy | 67.5 (0.5-214.9) |
| Chen, 2014 [61]^d^ | Taiwan | 2002-2006 | 261 | Stage I; TNM VII | Early stage | NA | - |
| Ichinose, 2014 [62] | Japan | 1998-2009 | 112 | N1-N2 patients; TNM VII | Advanced stage | Lobectomy, lymph nodes resection | 58.8 |
| Landreneau, 2014 [63] | USA | NA | 1192 | Stage I; TNM VII | Early stage | Segmentectomy, lobectomy | 63 |
| Peters, 2014 [64] | Europe/China | 2003-2009 | 2449 | Stage I-III; TNM VII | Any stage | Radical resection | 59.4 (47.5-76.5) |
| Rotolo, 2014 [65]^c^ | France | 1995-2001 | 1687 | NSCLC; TNM - | Any stage | Segmentectomy, lobectomy, pneumectomy | - |
| Su, 2014 [66]^c^ | USA | 1999-2004 | 1018 | T1 or T2, N0 or non-hilar N1, M0 NSCLC; TNM - | Early stage | Any | 80.4 |
| Lee, 2015 [67] | Korea | 2006-2011 | 249 | Stage I -II; TNM VII | Early stage | Any | 36.5 (IQR 28.0–52.8) |
| Ogawa, 2015 [68] | Japan | 2000-2009 | 53 | Stage I-III; TNM VII | Any stage | Thoracotomy | 33.6 |
| Varlotto, 2015 [69] | USA | 1995-2008 | 1568 | Stage I-IIIA; TNM VII | Any stage |  | 67.1 |
| Altorki, 2016 [70] | USA | 2000-2014 | 289 | cT1N0 (pT1-3,N0-2); TNM VII | Any stage | Segmentectomy, wedge resection | 32 (Wedge resection)/36 (Segmentectomy) |
| Billè, 2016 [71] | New York, USA | 2000-2012 | 446 | Stage I; TNM - | Early stage | Wedge resection | 44.6 |
| Borghetti, 2016 [72] | Italy | 2001-2011 | 285 | pN1; TNM VII | Advanced stage | NA | 39 (1-166) |
| Dziedzic, 2016 [73] | Poland | 2009-2014 | 14578 | NSCLC; TNM VII | Any stage | Complete surgical resection | 30.1 |
| Kim, 2016 [74] | Korea | 2005-2013 | 115 | Stage IB; TNM VII | Early stage | Any | 53 |
| Kodama, 2016 [75] | Japan | 1997-2010 | 312 | T1aN0M0; TNM VII | Early stage | Segmentectomy, lobectomy | 76.8 (Lobectomy)/82.1 (Segmentectomy) |
| Nishio, 2016 [76] | Japan | 1995-2009 | 190 | T1aN0M0 with C/T >0.5; TNM VII | Early stage | Segmentectomy or lobectomy | 107 (77-136) (Segmentectomy)/84 (70-105) (Lobectomy) |
| Wong, 2016 [77] | USA | 2006-2007 | 9001 | Stage I-III; TNM - | Any stage | Any | 60 for all patients |
| Stiles, 2017 [78] | New York, USA | 2000-2014 | 196 | Stage IA; TNM - | Early stage | Wedge resection | - |
| Tian, 2017 [79] | China | 2005-2011 | 521 | PN0; TNM VII | Early stage | Pneumonectomy and lymph nodes dissection | 41.4 |
| Aprile, 2018 [80] | Italy | 2006-2013 | 159 | Stage (T1-2, N0) NSCLC left upper lobe; TNM VII | Early stage | Lobectomy, trisegmentectomy, lingulectomy | 76 (28-122) |
| Brandt, 2018 [81] | USA | 2000-2016 | 893 | pT1-3N0M0, adenocarcinoma; TNM VIII | Early stage | R0 lobectomy | 35.0 (0.1-202) |
| Isaka, 2018 [82] | Japan | 2002-2012 | 1012 | Stage I–III; TNM VII | Any stage | Pneumonectomy and lymph nodes dissection | 59 (6-139) |
| Judy, 2018 [83] | USA | 2007-2015 | 217 | pStage T1-4, N0-1; TNM VII | Any stage | Lobectomy, bilobectomy, pneumonectomy | 36 (1-120) |
| Sung, 2018 [84] | Korea | 2000-2012 | 381 | T1–2N0; TNM VII | Early stage | Pneumonectomy, lobectomy | 53.9 |
| Zhong, 2018 [85] | Japan, China | 2000-2011 | 354 | T1aN0M0 (Stage IA, adenocarcinoma); TNM - | Early stage | Segmentectomy, wedge resection | 78 |
| Matsuura, 2019 [86] | Japan | 2006-2014 | 144 | cN0; TNM VIII | Early stage | Wedge resection | - |
| Schuchert, 2019 [87] | USA | NA | 1132 | c Stage I; TNM VIII | Early stage | Segmentectomy and Lobectomy | 34.2 (Segmentectomy)/38.8 (Lobectomy) |
| Kneuertz, 2020 [88] | USA | 2012-2017 | 514 | Stage I-IIIA; TNM VIII | Any stage | Lobectomy | 44.8 |
| Ma, 2020 [89] | China | 2013-2017 | 80 | pTlaN0M0; TNM VIII | Early stage | Segmentectomy, lobectomy | - |
| Mun, 2020 [90] | Japan | 2008-2016 | 54 | cT1-3N0M0/pN1-2; TNM - | Advanced stage | VATS lobectomy, lymph nodes dissection | 51.6 |
| Nakagawa, 2020 [91] | Japan | 2005-2016 | 377 | cN0-1 and pN2; TNM VIII | Advanced stage | Lobectomy and systematic LN dissection | 49 |
| Shimizu, 2020 [92] | Japan | 2004-2016 | 688 | NSCLC; TNM VIII | Any stage | Complete surgical resection and systematic lymphovascular dissection (lobectomy, bilobectomy, pneumonectomy) | 57.3 (4-194) |
| Vaghjiani, 2020 [93] | USA | 2000-2014 | 1055 | Stage IA; TNM - | Early stage | Lobectomy and lymph nodes dissection | - |
| Yoshida, 2020 [94] | Japan | 2000-2012 | 249 | pN1; TNM VII | Advanced stage | Lobectomy, bilobectomy, pneumonectomy | 66 (2.4-208) |
| Sato, 2021 [95] | Japan | 2010-2017 | 1775 | cN0-pStage I-III; TNM VIII | Any stage | Lobectomy, sublobar resection, pneumonectomy, VATS | 31.2 (0.5-94.4) |
| Hu, 2021 [96] | China | 2012-2016 | 169 | pStage IV-M1a; TNM VIII | Any stage | Lobectomy, pneumonectomy | 24 (11-48) (open-close group)/  39.5 (17.2-61.2) (resection group) |
| Schiavon, 2021 [97] | Italy | 2004-2016 | 313 | NSCLC; TNM VIII | Advanced stage | Lobectomy, bilobectomy, pneumonectomy, sleeve resection, wedge resection | 26.9 (13-75) (lobectomy)/  32.4 (11-81) (pneumonectomy) |
| Jiang, 2020 [98] | China | 2012-1018 | 107 | pStage IIIA-N2; TNM VII | Any stage | Lobectomy with systematic lymph node dissection | 31.2 |
| Sugita, 2021 [99] | Japan | 2004-2016 | 454 | Completely resected adenocarcinoma; TNM VIII | Early stage | Lower lobectomy or superior segmentectomy | - |
| Dolan, 2021 [100] | USA | 2000-2018 | 196 | pStage I; TNM VIII | Advanced stage | Lobectomy, bilobectomy, pneumonectomy | 45.6 (20.4-81.6) |

IQR: interquartile range; VATS: Video-assisted thoracoscopic surgery.

^a^Early stage: all studies that only include patients with pT1-T2 N0 tumors; advanced stage: studies that include other pathological stages; any stage: studies that include all pathological stages. ^b^One of 548 patient was stage 4 and was assumed to be due to tumours in separate ipsilateral lobes, which are now classified as T4 tumours. ^c^Trial. ^d^Two of 261 patients were stage 4 patients and were assumed to be due to tumours in separate ipsilateral lobes, which are now classified as T4 tumours.

**Supplementary Table 4.** Recurrence information of the studies on risk factors for locoregional recurrence after surgical resection of non-small cell lung cancer included in the meta-analysis.

| **Study reference** | **Recurrence diagnostic** | **Definition of recurrence (Definition group)** | **N. locoregional recurrence (%)** | **N. Distal recurrence (%)** |
| --- | --- | --- | --- | --- |
| Okada, 1999 [15] | NA | Local: ipsilateral hemithorax. (Most inclusive) | 11 (9.2) | - |
| Mineo, 2001 [16] | Imaging, histology | NA | 15 (22.7) | 24 (36.4) |
| Maruyama, 2004 [17] | NA | NA | 16 (11.2) | 34 (23.8) |
| Lardinois, 2005 [18] | X-ray, laboratory findings, CT, biopsy, autopsy | NA | 25 (27.2) | 31 (33.7) |
| El-Sherif, 2006 [19] | CT | Locoregional: same lobe of the lung, or interlobar and hilar lymph nodes. Distal: all other metastases. (Least inclusive) | 75 (9.6) | 147 (18.8) |
| Fujimoto, 2006 [20] | NA | Locoregional: cell type was the same as the original and was located within the ipsilateral hemithorax, mediastinum, or supraclavicular lymph node chain. Distal: all other sites of recurrence. (Most inclusive) | 25 (13.9) | 27 (15) |
| Shiraishi, 2006 [21] | NA | Locoregional: mediastinal or hilar lymph node, pleural space (pleural dissemination), or surgical margin, and simultaneous recurrences. Distal: organ including a separate lung lobe other than the target lobe. (Less inclusive) | 12 (7.5) | 14 (8.8) |
| Schuchert, 2007 [22] | CT | Locoregional: same lobe, the hilum, or the mediastinal lymph nodes. Distal: another lobe, or elsewhere outside the hemithorax. (Less inclusive) | 26 (6.1) | 47 (11) |
| Sienel, 2007 [23] | x-rays and CT | Local: within the same lung or in the ipsilateral mediastinum. (Most inclusive) | 16 (8) | 25 (12.6) |
| Tsuchiya, 2007 [24] | NA | NA | 28 (8.7) | 17 (5.3) |
|  |  |  |  |  |
| Shiraishi, 2008 [25] | NA | Locoregional: mediastinal or hilar lymph node, pleural space (pleural dissemination), or surgical margin. Distal: organ including a separate lung lobe other than the target lobe. (Less inclusive) | - | - |
| Sienel, 2008 [26] | TAC, Scintigraphy | Local: same lung or in the ipsilateral mediastinum. (Most inclusive) | 23 (26.4) | 13 (14.9) |
| Voltolini, 2008 [27] | Physical examination, tumor markers, chest X-ray, Bronchoscopy and CT | Local: disease within the pleural cavity. Distal: all other sites of recurrence. (Most inclusive) | 21 (11.7) | 34 (19) |
| Bando, 2009 [28] | Physical and biochemical examinations, tumor markers, and chest X-ray. CT of the chest, upper abdomen, and brain, bone scintigraphy | Locoregional: ipsilateral hemithorax (lung, mediastinum and pleura). Distal: Tumors that recurred simultaneously both in the ipsilateral hemithorax and distant sites. (Most inclusive) | 11 (12.1) | 8 (8.8) |
| De Giacomo, 2009 [29] | CT | Local: hemithorax. Distal: metastatic disease without local recurrence within the ipsilateral hemithorax. (Most inclusive) | 17 (11.2) | 31 (20.9) |
| Schuchert, 2009 [30] | CT | Locoregional: same lobe, the hilum, or the mediastinal lymph nodes. Distal: another lobe or elsewhere outside the hemithorax. (Less inclusive) | 18 (8) | 28 (12.4) |
| Shapiro, 2009 [31] | NA | NA | 11 (7.6) | 22 (15.3) |
| Varlotto, 2009 [11] | CT | Local: ipsilateral lung, bronchial stump or staple line, and lymph node regions (subcarinal, periesophageal, ipsilateral or contralateral mediastinum, supraclavicular, or hilar lymph nodes). Distal: liver, contralateral lung, adrenal glands, brain, bone, or other locations. (Most inclusive) | 61 (16.4) | 34 (9.1) |
| Kim, 2010 [32] | CT | Locoregional: ipsilateral pleura, ipsilateral lung, mediastinal lymph nodes, hilar lymph nodes, and stump. (Most inclusive) | 15 (2.7) | 34 (6.2) |
| Okami, 2010 [33] | Daily clinical practice | Locoregional: same lobe, the mediastinal lymph nodes, or the hilum. Distal: all other types of recurrence. (Less inclusive) | 26 (3.4) | 90 (11.8) |
| Saynak, 2010 [34] | CT | Locoregional: ipsilateral lung, stump, chest wall, mediastinum, or supraclavicular lymph nodes. Simultaneuos recurrences considered as local. (Most inclusive) | 23 (19.3) | - |
| Shoji, 2010 [35] | CT, MRI, bone scintigram, and fluorodeoxyglucose-PET | Local: hilar or mediastinal lymph nodes, pleural cavity, bronchial stump, or staple line. Distal: brain, lung, adrenal glands, bone, and other locations. (Most inclusive) | 12 (5.5) | 39 (18) |
| Darling, 2011 [36] | NA | NA | 128 (12.5) | 225 (22) |
| Koo, 2011 [37] | Clinical assessment, radiographic report, and supplemental data from bronchoscopy, biopsy, and PET | Local: ipsilateral lung, bronchial stump or staple line, and regional lymph node (subcarinal, periesophageal, ipsilateral or contralateral mediastinum, supraclavicular, or hilar lymph nodes). Distal: contralateral lung, liver, adrenal glands, brain, bone, or other locations. (Most inclusive) | 27 (8.7) | 29 (9.4) |
| Park, 2011 [38] | NA | NA | 11 (2.1) | 22 (4.2) |
| Pepek, 2011 [39] | Imaging studies and as bronchoscopy, mediastinoscopy, or CT-guided biopsies | Locoregional: surgical margin, ipsilateral hilum, and/or mediastinum. Distal: contralateral hilum, supraclavicular fossae, ipsilateral lung parenchyma, or elsewhere. (Less inclusive) | 100 (14.1) | - |
| Saynak, 2011 [40] | Thorax CT,upper abdomen CT or sonogram | Locoregional: ipsilateral lung, stump, chest wall, mediastinum, or supraclavicular lymph nodes. (Most inclusive) | 40 (11.9) | 32 (9.6) |
| Schuchert, 2011 [41] | CT | Locoregional: same lobe, hilum, or mediastinal lymph nodes. Distal: another lobe, or elsewhere outside the hemithorax. (Less inclusive) | 32 (6.1) | 80 (15.3) |
| Varlotto, 2011 [42] | CT | Local: ipsilateral lung and the N1–N3 nodal basins. Distal: all other recurrences. (Most inclusive) | 18 (30) | 14 (23.3) |
| Wolf, 2011 [43] | NA | Local: same lobe, hilum, or mediastinum with or without the presence of distal metastases. (Less inclusive) | 32 (6.8) | 34 (7.3) |
| Xie, 2011 [44] | CT or PET | Locoregional: site of surgical resection (e.g., ipsilateral lung, surgical stump, hilum, chest wall, mediastinum). Distal: beyond either the surgical bed or regional lymph nodes. (Most inclusive) | 22 (24.4) | 15 (16.7) |
| Carr, 2012 [45] | CT | Locoregional: same lobe, hilum, or mediastinal lymph nodes. Distal: another lobe or elsewhere outside the hemithorax. (Less inclusive) | 25 (5.8) | 51 (11.9) |
| Higgins, 2012 [46] | Imaging, CT-guided transthoracic biopsies, bronchoscopy, endobronchial ultrasound, and mediastinoscopy | Local: surgical resection margin or regional lymph nodes (ipsilateral hilum and/or mediastinum). (Less inclusive) | - | - |
| Higgins, 2012 [47] | (CT)-guided transthoracic biopsies, bronchoscopy, endobronchial ultrasound, and mediastinoscopy | Local: surgical resection margin, ipsilateral hilum, and/or mediastinum. Distal: all other sites of failure, including the supraclavicular fossa and contralateral hilum. (Less inclusive) | 38 (19.2) | - |
| Hung, 2012 [48] | NA | Local: contiguous anatomical sites, including the ipsilateral hemithorax and mediastinum after surgical resection. Distal: contralateral lung or outside the hemithorax and mediastinum. (Most inclusive) | 51 (6.7) | 126 (16.7) |
| Hung, 2012 [49] | NA | Local: contiguous anatomic site, including the ipsilateral hemithorax and mediastinum, after surgical resection. Distal: contralateral lung or outside the hemithorax and mediastinum. (Most inclusive) | 20 (5.6) | 59 (16.6) |
| Stefani, 2012 [50] | NA | Locoregional: same lobe or lung, hilum or mediastinal lymph nodes. Distal: recurrence in the controlateral lung or elsewhere outside the thorax. (Most inclusive) | 28 (13.6) | 19 (9.2) |
| Varlotto, 2012 [51] | PET-CT | Local: ipsilateral lungs and the N1–N3 nodal basins. Distal: all other recurrences. (Most inclusive) | - | - |
| Choi, 2013 [52] | Physical exam, diagnostic imaging | Local: contiguous anatomic sites, including the surgical margin, ipsilateral hemithorax, and mediastinum after surgical resection. Distal: contralateral lung or outside the hemithorax and mediastinum. (Most inclusive) | 26 (10.7) | 51 (21.1) |
| Fan, 2013 [53] | Medical, imaging, cytological and pathological reports, CT or PET-CT | Local: bronchial stump or anastomosis. Regional: mediastinum, hilum or supraclavicular fossa. Distal: other sites of recurrence, including contralateral lung and metastatic lymph nodes in the neck or axilla. (Less inclusive) | 41 (20.6) | 79 (39.7) |
| Kelsey, 2013 [54] | NA | Local: surgical resection margin, ipsilateral hilum, and/or mediastinum. Distal: all other sites of recurrence, including the supraclavicular fossa and contralateral hilum. (Most inclusive) | Primary  199 (14.1)  Validation  146 (16.4) | - |
| Koike, 2013 [55] | CT or PET/CT | Locoregional: same lobe, or in the ipsilateral thoracic cavity, and hilum or mediastinal lymph nodes. Newly developed lung lesions only in the same lobe unless the lesions showed different histologic subtypes from a primary lung carcinoma. (Less inclusive) | 51 (15.5) | - |
| Lopez Guerra, 2013 [56] | Biopsy, CT, PET, MRI, bone scan | Local: surgical resection margin. Regional: mediastinal, hilar, and supraclavicular fossa recurrence. Distal: all other sites of recurrence, and recurrence at a different lobe of the lung (same histology), with N2-N3 involvement or <2 years interval after the surgery. (Less inclusive) | 98 (7) | - |
| Tandberg, 2013 [57] | PET/CT | Local: surgical resection margin, ipsilateral hilum, and/or mediastinum. Distal: all other sites of recurrence. (Less inclusive) | 78 (14.3) | - |
| Varlotto, 2013 [58] | CT or PET/CT scan | Local: ipsilateral lungs and the N1-N3 nodal basins. Distal: all other recurrences. (Most inclusive) | 84 (20.4) | 47 (11.4) |
| Washington, 2013 [59] | Radiological imaging | Locoregional: surgical resection margin (bronchial stump or wedge resection line), ipsilateral hilum, and/or mediastinum. Distal: all other sites of failure, including the supraclavicular fossa, contralateral hilum, and ipsilateral lung parenchyma. (Less inclusive) | 208 (21.7) | 281 (29.4) |
| Yanagawa, 2013 [60] | CT | Local: same diseased hemithorax, mediastinum and pleuritis carcinomatosa. Distal: all other sites of recurrence. (Most inclusive) | 36 (8.3) | 32 (7.4) |
| Chen, 2014 [61] | Chest CT,PET CT or MRI | Locoregional: contiguous anatomical sites, including the ipsilateral hemithorax and ipsilateral mediastinal or hilar lymph nodes, or both after surgical resection. Distal: contralateral lung or outside the hemithorax and mediastinum. (Most inclusive) | 17 (6.5) | 20 (7.7) |
| Ichinose, 2014 [62] | Chest radiography, CT | Locoregional: hilar or mediastinal lymph nodes, surgical margin, or the ipsilateral pleural space, and simultaneous recurrences. Distal: distant organs, including in a separate lung. (Most inclusive) | 16 (14.3) | 22 (19.6) |
| Landreneau, 2014 [63] | CT | Locoregional: same lobe, the hilum, or the mediastinal lymph nodes. Distal: another lobe, the pleural space, or elsewhere outside the hemithorax. (Less inclusive) | 33 (2.8) | 82 (6.9) |
| Peters, 2014 [64] | NA | NA | 248 (10.1) | 424 (17.3) |
| Rotolo, 2014 [65] | NA | NA | 401 (23.8) | 689 (40.8) |
| Su, 2014 [66] | CT or PET/CT | Local: adjacent lung parenchyma, bronchial stump, or the hilum adjacent to the bronchial stump. Regional: hilum (separate from bronchial stump), mediastinum, chest wall, or ipsilateral pleura. Distal: separate lobe of ipsilateral lung, contralateral thorax, supraclavicular lymph nodes, or distant organ. (Less inclusive) | 98 (9.6) | 167 (16.4) |
| Lee, 2015 [67] | Clinical assess, radiographic reports, and/or data from a biopsies | Locoregional: ipsilateral lobe of the lung, bronchial stump, or a regional lymph node. Distal: liver, contralateral lung, adrenal glands, brain bone, or other location, and simultaneous recurrences. (Least inclusive) | 24 (9.6) | 33 (13.3) |
| Ogawa, 2015 [68] | Chest radiography, CT and haematological and biochemical analyses including tumor markers | Local: same lung, ipsilateral lymph nodes or pulmonary hilum. Distal: any otherrecurrence. (Most inclusive) | 16 (30.2) | 23 (43.4) |
| Varlotto, 2015 [69] | Imaging studies, bronchoscopy or PET | Locoregional: surgical resection margin, ipsilateral hilum, and/or mediastinum. Distal: all other sites of recurrence, including the supraclavicular fossa and contralateral hilum. (Less inclusive) | 220 (14) | - |
| Altorki, 2016 [70] | Chest and upper abdomen CT, histopathologic proof | Local: similar histology within the same lobe.  Regional: ipsilateral hilar or mediastinal lymph nodes. Distal: another lobe within 2 years from surgery, the pleural space, or elsewhere outside the hemithorax. (Less inclusive) | 29 (10) | 27 (9.3) |
| Billè, 2016 [71] | PET/MRI | Local: same lobe of the resection. Regional: involving the mediastinal or hilar lymph nodes or a different ipsilateral lobe from the location of the wedge resection. Distal: including distant metastasis to other organs and diffuse pleural disease. (Most inclusive) | 124 (27.8) | 28 (6.3) |
| Borghetti, 2016 [72] | Medical records, reports from GP, radiographical exams | Local: bronchial stump, ipsilateral hilum and mediastinum. Distal: all other sites of failure, including supraclavicular fossa and contralateral hilum. (Less inclusive) | 68 (23.9) | 116 (40.7) |
| Dziedzic, 2016 [73] | PET-CT | Local: ipsilateral hemithorax, mediastinum, or supraclavicular lymph nodes. Distal: all other sites. (Most inclusive) | 578 (4) | 2238 (15.4) |
| Kim, 2016 [74] | CT | Local: resection margins, such as bronchial stumps or stapler lines. Regional: hilar or mediastinal lymph nodes, pleural cavity, or ipsilateral lung. Distal: the contralateral lung, brain, liver, adrenal glands, bone, and other locations. (Most inclusive) | 15 (13) | 20 (17.4) |
| Kodama, 2016 [75] | CT | Locoregional: same lobe (i.e., resection margin), ipsilateral hilum, mediastinum, or pleural space as the first relapse site. (Most inclusive) | 18 (5.8) | - |
| Nishio, 2016 [76] | Chest CT | Local: same lobe (i.e., resection margin and intrapulmonary metastases). Regional: ipsilateral hilum or mediastinum. (Most inclusive) | 27 (14.2) | - |
| Wong, 2016 [77] | Physician examination | Locoregional: ipsilateral lung and/or regional lymph nodes. Distal: outside the ipsilateral chest. (Most inclusive) | 1110 (12.3) | 1933 (21.5) |
| Stiles, 2017 [78] | Chest and upper abdomen CT | Local: tumor with similar histology within the same lobe. Regional: tumor in the ipsilateral hilar and/or mediastinal LNs. Distal: tumor in another lobe within 2 years from surgery, pleural space or else- where outside the hemi-thorax. (Less inclusive) | 16 (8.2) | 15 (7.7) |
| Tian, 2017 [79] | NA | Regional: surgical margin or bronchus staple line or involvement of the ipsilateral mediastinal and/or ipsilateral hilar lymph nodes. Distal: all locations outside of locoregional recurrence and included supraclavicular nodes, contralateral mediastinal or hilar nodes, another lobe in the ipsilateral lung, or any tissue or organ outside of the locoregional recurrence zone, and simultaneous recurrences. (Less inclusive) | 12 (2.3) | 43 (8.3) |
| Aprile, 2018 [80] | Physical examinations, hematologic, and biochemical analysis including tumormarkers and contrast-enhanced chest CT and superior abdomen ultrasonography | Locoregional: same lung, the hilar, or the omolateral mediastinal lymph nodes. Distal: pleural carcinosis or as the evidence of tumor in the contralateral hemithorax or in any other distal site. (Most inclusive) | 14 (8.8) | 25 (15.7) |
| Brandt, 2018 [81] | CT/PET | Locoregional: surgical margin or bronchus staple line or involvement of the ipsilateral mediastinal and/or ipsilateral hilar lymph nodes. Distal: all locations outside of locoregional recurrence and included supraclavicular nodes, contralateral mediastinal or hilar nodes, another lobe in the ipsilateral lung, or any tissue or organ outside of the locoregional recurrence zone, and simultaneous recurrences. (Less inclusive) | 16 (1.8) | 99 (11.1) |
| Isaka, 2018 [82] | CT | Local: surgical resection margin, ipsilateral hilum, and ipsilateral or contralateral mediastinum. Distal: supraclavicular fossa, contralateral hilum, and ipsilateral lobe of the lung. (Less inclusive) | 95 (9.4) | 224 (22.1) |
| Judy, 2018 [83] | CT | Locoregional: ipsilateral lung, bronchial stump, mediastinum, chest wall, or supraclavicular region. (Most inclusive) | 42 (19.4) | 6 (2.8) |
| Sung, 2018 [84] | CT | Local: initial site of the primary tumor. Nodal recurrence: mediastinal and/or hilar nodal recurrence. Distal: recurrence involving a different pulmonary lobe or another organ. (Less inclusive) | 49 (12.9) | 69 (18.1) |
| Zhong, 2018 [85] | Radiography, histology | NA | 15 (4.2) | 17 (4.8) |
| Matsuura, 2019 [86] | CT, MRI-PET | Local: staple line, hilar or mediastinal lymph nodes, or pleural cavity. (Most inclusive) | 29 (20.1) | 7 (4.9) |
| Schuchert, 2019 [87] | CT | Locoregional: same lobe, the hilum or the mediastinal lymph nodes. Distal: another lobe, the pleural space, or elsewhere outside the hemithorax. (Less inclusive) | 65 (5.7) | 155 (13.7) |
| Kneuertz, 2020 [88] | Non-contrast enhanced CT | Locoregional: ipsilateral resection bed and/or interlobar, hilar, or mediastinal lymph nodes. Distal: all other metastases. (Less inclusive) | 37 (7.2) | 77 (15) |
| Ma, 2020 [89] | CT | NA | 13 (16.3) | - |
| Mun, 2020 [90] | CT, PET-CT | NA | 13 (24.1) | 15 (27.8) |
| Nakagawa, 2020 [91] | CT | Local: surgical resection margin. Regional: ipsilateral or contralateral hilum, ipsilateral or contralateral mediastinum or ipsilateral or contralateral supraclavicular fossa. (Less inclusive) | 56 (14.9) | 116 (30.8) |
| Shimizu, 2020 [92] | High-resolution CT, PET | Local: contiguous anatomical sites, including the ipsilateral hemithorax and mediastinum after resection. (Most inclusive) | 89 (12.9) | 104 (15.1) |
| Vaghjiani, 2020 [93] | CT, PET | Local: staple line or the lung parenchyma within the treated lobe. Regional lymph nodes: recurrence was defined as recurrence within the ipsilateral hilar or mediastinal lymph nodes. Regional lung: ipsilateral lobes other than the resected lobe. (Most inclusive) | 62 (5.9) | 40 (3.8) |
| Yoshida, 2020 [94] | CT, PET-CT, Biopsy | Local, regional and distal defined according to the American College of Chest Physicians guidelines. (Most inclusive) | 16 (6.4) | 93 (37.3) |
| Sato, 2021 [95] | CT, chest radiography, blood examination, brain MRI, bone scintigraphy, PET, histological examinations | NA | 57 (3.2) | 126 (7.1) |
| Hu, 2021 [96] | CT, MRI, PET | Local: primary lesion enlargement or lesion recurrence at the resection site. Regional progression defined as increasing pleural effusion, pleural nodules, lung lesions, or ipsilateral lymph node recurrence, enlargement. Distal: new lesions in the contralateral lung or any other organ (brain, bone, etc.). (Most inclusive) |  |  |
| Schiavon, 2021 [97] | NA | NA | 25 (8) | 113 (36.1) |
| Jiang, 2020 [98] | Physical examination, serum tumor markers, chest, brain or abdomen CT, ultrasonography, MRI | Locoregional: tumor recurrence at stumps and/or regional lymph nodes, including hilar, mediastinal, and supraclavicular lymph nodes. Intrapulmonary metastasis in the affected lung was not considered.  Distal: non-regional tumor recurrence, including metastases to cervical lymph nodes, the contralateral lung, pleura, brain, bone, liver, or adrenal glands. (Less inclusive) | 13 (12.1) | 23 (21.5) |
| Sugita, 2021 [99] | CT chest, upper abdomen scans or brain MRI | Local: tumor recurrence in contiguous anatomical sites, including the ipsilateral hemithorax and mediastinum, after resection. Distal: tumor recurrence in the contralateral lung or outside the hemithorax and mediastinum after resection. If both local and distant recurrence was noted within 3 months at the time of initial recurrence, these cases were classified into the distal metastasis group. (Most inclusive) | 54 (11.9) | 95 (20.9) |
| Dolan, 2021 [100] | Physical examinations and imaging | Locoregional: cancer recurrence within the ipsilateral lobe, ipsilateral hilar or mediastinal lymph nodes, or death. Distal: other sites of recurrence. (Less inclusive) | 12 (6.1) | 26 (13.3) |

CT: computed tomography; MRI: Magnetic Resonance Imaging; NA: not available; PET: positron emission tomography.

Grouping of locoregional recurrence definitions: Most inclusive – Ipsilateral hemithorax and mediastinal lymph nodes, with or without supraclavicular nodes, with or without pleural space; Less inclusive – Same lobe and hilar and mediastinal nodes, with or without supraclavicular nodes, with or without pleural space; Least inclusive – Same lobe and hilar and interlobar nodes (no mediastinal nodes); No definition (NA)

**Supplementary Table 5. P**ublication bias for studies on selected risk factors for locoregional recurrence after surgical resection of non-small cell lung cancer.

| **Risk factor** | **Begg's test**  **p-value** | **Egger's test**  **p-value** |
| --- | --- | --- |
| Sex | 0.112 | 0.009 |
| Age |  |  |
| Categorical | 0.869 | 0.182 |
| Continuous (years) | 0.222 | 0.022 |
| Tobacco smoking | 0.040 | 0.064 |
| Adjuvant chemotherapy | 0.884 | 0.918 |
| Surgical procedure |  |  |
| Pneumonectomy vs lobectomy | 0.784 | 0.864 |
| Segmentectomy/wedge vs lobectomy | 0.950 | 0.354 |
| Sublobar NOS vs lobar resection | -^a^ | -^a^ |
| Lobectomy (VATS vs open) | 0.656 | 0.361 |
| Lymph node resected | **-^a^** | -^a^ |
| Lymphovascular invasion | 0.229 | 0.058 |
| (Visceral) pleural invasion | 0.321 | 0.140 |
| Pathological stage |  |  |
| II vs I | -^a^ | -^a^ |
| III-IV vs I | -^a^ | -^a^ |
| Ib-II vs Ia | -^a^ | -^a^ |
| III-IV vs Ia | -^a^ | -^a^ |
| Tumor T stage |  |  |
| T2 vs T1 | -^a^ | -^a^ |
| T3-T4 vs T1 | -^a^ | -^a^ |
| Tumor N stage |  |  |
| N1 vs N0 | -^a^ | -^a^ |
| N2 vs N0 | -^a^ | -^a^ |
| N1-N2 vs N0 | 0.702 | 0.935 |
| Tumor grade |  |  |
| Moderate/poor vs well | -^a^ | -^a^ |
| Poor vs well/ moderate | -^a^ | -^a^ |
| Tumor histology |  |  |
| Non adenocarcinoma vs adenocarcinoma | 0.240 | 0.202 |
| Non squamous cell vs squamous cell | 0.346 | 0.096 |
| Tumor location |  |  |
| Other location vs right upper lobe | -^a^ | -^a^ |
| Left lobe vs right lobe | -^a^ | -^a^ |
| Tumor size |  |  |
| Categorical (large vs small) | 0.382 | 0.023 |
| Continuous (cm) | -^a^ | -^a^ |

VATS: Video-assisted thoracoscopic surgery.

^a^ Not computed, less than 10 studies.

**Supplementary Table 6.** Assessment of study quality according to the Newcastle-Ottawa scale.

| **Study reference** | **Selection** | | | | **Comparability** | **Outcome** | | | **Total score** |
| --- | --- | --- | --- | --- | --- | --- | --- | --- | --- |
|  | **A** | **B** | **C** | **D** | **E** | **F** | **G** | **H** |  |
| Okada, 1999 [15] | 1 | 1 | 1 | 1 | 0 | 0 | 1 | 1 | 6 |
| Mineo, 2001 [16] | 1 | 1 | 1 | 1 | 0 | 0 | 1 | 1 | 6 |
| Maruyama, 2004 [17] | 1 | 1 | 1 | 1 | 0 | 0 | 0 | 1 | 5 |
| Lardinois, 2005 [18] | 1 | 1 | 1 | 1 | 0 | 0 | 1 | 1 | 6 |
| El-Sherif, 2006 [19] | 1 | 1 | 1 | 1 | 0 | 1 | 0 | 1 | 6 |
| Fujimoto, 2006 [20] | 1 | 1 | 1 | 1 | 0 | 0 | 0 | 1 | 5 |
| Shiraishi, 2006 [21] | 1 | 1 | 1 | 1 | 2 | 0 | 1 | 1 | 8 |
| Schuchert, 2007 [22] | 1 | 1 | 1 | 1 | 0 | 1 | 0 | 1 | 6 |
| Sienel, 2007 [23] | 1 | 1 | 1 | 1 | 0 | 1 | 1 | 1 | 7 |
| Tsuchiya, 2007 [24] | 1 | 1 | 1 | 1 | 0 | 0 | 0 | 1 | 5 |
| Shiraishi, 2008 [25] | 1 | 1 | 1 | 1 | 1 | 0 | 1 | 1 | 7 |
| Sienel, 2008 [26] | 1 | 1 | 1 | 1 | 0 | 1 | 1 | 1 | 7 |
| Voltolini, 2008 [27] | 1 | 1 | 1 | 1 | 0 | 1 | 1 | 1 | 7 |
| Bando, 2009 [28] | 0 | 1 | 1 | 1 | 0 | 1 | 1 | 1 | 6 |
| De Giacomo, 2009 [29] | 1 | 1 | 1 | 1 | 0 | 1 | 1 | 1 | 7 |
| Schuchert, 2009 [30] | 1 | 1 | 1 | 1 | 0 | 1 | 0 | 1 | 6 |
| Shapiro, 2009 [31] | 1 | 1 | 1 | 1 | 1 | 0 | 0 | 1 | 6 |
| Varlotto, 2009 [11] | 1 | 1 | 1 | 1 | 2 | 1 | 0 | 1 | 8 |
| Kim, 2010 [32] | 1 | 1 | 1 | 1 | 0 | 1 | 0 | 1 | 6 |
| Okami, 2010 [33] | 1 | 1 | 1 | 1 | 0 | 1 | 0 | 1 | 6 |
| Saynak, 2010 [34] | 1 | 1 | 1 | 1 | 2 | 1 | 1 | 1 | 9 |
| Shoji, 2010 [35] | 1 | 1 | 1 | 1 | 0 | 1 | 1 | 1 | 7 |
| Darling, 2011 [36] | 1 | 1 | 1 | 1 | 0 | 0 | 1 | 1 | 6 |
| Koo, 2011 [37] | 1 | 1 | 1 | 1 | 0 | 1 | 1 | 1 | 7 |
| Park, 2011 [38] | 1 | 1 | 1 | 1 | 0 | 0 | 0 | 1 | 5 |
| Pepek, 2011 [39] | 1 | 1 | 1 | 1 | 0 | 1 | 0 | 1 | 6 |
| Saynak, 2011 [40] | 1 | 1 | 1 | 1 | 2 | 1 | 0 | 1 | 8 |
| Schuchert, 2011 [41] | 1 | 1 | 1 | 1 | 0 | 1 | 1 | 1 | 7 |
| Varlotto, 2011 [42] | 1 | 1 | 1 | 1 | 2 | 1 | 0 | 1 | 8 |
| Wolf, 2011 [43] | 1 | 1 | 1 | 1 | 0 | 0 | 0 | 1 | 5 |
| Xie, 2011 [44] | 1 | 1 | 1 | 1 | 1 | 1 | 1 | 1 | 8 |
| Carr, 2012 [45] | 1 | 1 | 1 | 1 | 0 | 1 | 1 | 1 | 7 |
| Higgins, 2012 [46] | 1 | 1 | 1 | 1 | 2 | 1 | 0 | 1 | 8 |
| Higgins, 2012 [47] | 1 | 1 | 1 | 1 | 2 | 1 | 0 | 1 | 8 |
| Hung, 2012 [48] | 1 | 1 | 1 | 1 | 1 | 0 | 1 | 1 | 7 |
| Hung, 2012 [49] | 1 | 1 | 1 | 1 | 2 | 0 | 1 | 1 | 8 |
| Stefani, 2012 [50] | 1 | 1 | 1 | 1 | 0 | 0 | 1 | 1 | 6 |
| Varlotto, 2012 [51] | 1 | 1 | 1 | 1 | 2 | 1 | 0 | 1 | 8 |
| Choi, 2013 [52] | 1 | 1 | 1 | 1 | 0 | 1 | 1 | 1 | 7 |
| Fan, 2013 [53] | 1 | 1 | 1 | 1 | 1 | 1 | 1 | 1 | 8 |
| Kelsey, 2013 [54] | 1 | 1 | 1 | 1 | 1 | 0 | 0 | 1 | 6 |
| Koike, 2013 [55] | 1 | 1 | 1 | 1 | 2 | 1 | 1 | 1 | 9 |
| Lopez Guerra, 2013 [56] | 1 | 1 | 1 | 1 | 2 | 1 | 1 | 1 | 9 |
| Tandberg, 2013 [57] | 1 | 1 | 1 | 1 | 2 | 1 | 0 | 1 | 8 |
| Varlotto, 2013 [58] | 1 | 1 | 1 | 1 | 1 | 1 | 0 | 1 | 7 |
| Washington, 2013 [59] | 1 | 1 | 1 | 1 | 2 | 1 | 0 | 1 | 8 |
| Yanagawa, 2013 [60] | 1 | 1 | 1 | 1 | 0 | 1 | 1 | 1 | 7 |
| Chen, 2014 [61] | 1 | 1 | 1 | 1 | 1 | 1 | 0 | 1 | 7 |
| Ichinose, 2014 [62] | 1 | 1 | 1 | 1 | 0 | 1 | 1 | 1 | 7 |
| Landreneau, 2014 [63] | 1 | 1 | 1 | 1 | 0 | 1 | 1 | 1 | 7 |
| Peters, 2014 [64] | 1 | 1 | 1 | 1 | 0 | 0 | 1 | 1 | 6 |
| Rotolo, 2014 [65] | 1 | 1 | 1 | 1 | 0 | 0 | 0 | 1 | 5 |
| Su, 2014 [66] | 1 | 1 | 1 | 1 | 2 | 1 | 1 | 1 | 9 |
| Lee, 2015 [67] | 1 | 1 | 1 | 1 | 1 | 1 | 1 | 1 | 8 |
| Ogawa, 2015 [68] | 1 | 1 | 1 | 1 | 0 | 1 | 0 | 1 | 6 |
| Varlotto, 2015 [69] | 1 | 1 | 1 | 1 | 1 | 1 | 1 | 1 | 8 |
| Altorki, 2016 [70] | 1 | 1 | 1 | 1 | 0 | 1 | 1 | 0 | 6 |
| Billè, 2016 [71] | 1 | 1 | 1 | 1 | 0 | 1 | 1 | 1 | 7 |
| Borghetti, 2016 [72] | 1 | 1 | 1 | 1 | 2 | 1 | 1 | 1 | 9 |
| Dziedzic, 2016 [73] | 1 | 1 | 1 | 1 | 2 | 1 | 0 | 1 | 8 |
| Kim, 2016 [74] | 0 | 1 | 1 | 1 | 0 | 1 | 1 | 1 | 6 |
| Kodama, 2016 [75] | 1 | 1 | 1 | 1 | 1 | 1 | 1 | 1 | 8 |
| Nishio, 2016 [76] | 1 | 1 | 1 | 1 | 2 | 1 | 1 | 1 | 9 |
| Wong, 2016 [77] | 1 | 1 | 1 | 1 | 0 | 1 | 1 | 1 | 7 |
| Stiles, 2017 [78] | 1 | 1 | 1 | 1 | 0 | 1 | 0 | 1 | 6 |
| Tian, 2017 [79] | 1 | 1 | 1 | 1 | 0 | 0 | 1 | 1 | 6 |
| Aprile, 2018 [80] | 1 | 1 | 1 | 1 | 0 | 1 | 1 | 1 | 7 |
| Brandt, 2018 [81] | 1 | 1 | 1 | 1 | 0 | 1 | 0 | 1 | 6 |
| Isaka, 2018 [82] | 1 | 1 | 1 | 1 | 2 | 1 | 1 | 1 | 9 |
| Judy, 2018 [83] | 1 | 1 | 1 | 1 | 0 | 1 | 1 | 1 | 7 |
| Sung, 2018 [84] | 1 | 1 | 1 | 1 | 0 | 1 | 1 | 1 | 7 |
| Zhong, 2018 [85] | 1 | 1 | 1 | 1 | 0 | 0 | 1 | 1 | 6 |
| Matsuura, 2019 [86] | 0 | 1 | 1 | 1 | 1 | 1 | 0 | 1 | 6 |
| Schuchert, 2019 [87] | 1 | 1 | 1 | 1 | 2 | 1 | 0 | 1 | 8 |
| Kneuertz, 2020 [88] | 1 | 1 | 1 | 1 | 0 | 1 | 1 | 1 | 7 |
| Ma, 2020 [89] | 1 | 1 | 1 | 1 | 2 | 0 | 0 | 1 | 7 |
| Mun, 2020 [90] | 1 | 1 | 1 | 1 | 0 | 0 | 1 | 1 | 6 |
| Nakagawa, 2020 [91] | 1 | 1 | 1 | 1 | 1 | 1 | 1 | 1 | 8 |
| Shimizu, 2020 [92] | 1 | 1 | 1 | 1 | 0 | 1 | 1 | 1 | 7 |
| Vaghjiani, 2020 [93] | 1 | 1 | 1 | 1 | 0 | 1 | 0 | 1 | 6 |
| Yoshida, 2020 [94] | 1 | 1 | 1 | 1 | 0 | 1 | 1 | 1 | 7 |
| Sato, 2021 [95] | 1 | 1 | 1 | 1 | 2 | 0 | 0 | 1 | 7 |
| Hu, 2021 [96] | 1 | 1 | 1 | 1 | 2 | 1 | 0 | 1 | 8 |
| Schiavon, 2021 [97] | 1 | 1 | 1 | 1 | 0 | 0 | 0 | 1 | 5 |
| Jiang, 2020 [98] | 1 | 1 | 1 | 1 | 2 | 1 | 0 | 1 | 8 |
| Sugita, 2021 [99] | 1 | 1 | 1 | 1 | 1 | 1 | 0 | 1 | 7 |
| Dolan, 2021 [100] | 1 | 1 | 1 | 1 | 1 | 1 | 1 | 1 | 8 |

A: representativeness of the exposed cohort; B: selection of the non-exposed cohort; C: ascertainment of exposure; D: demonstration that outcome of interest was not present at start of study; E: comparability of cohorts on the basis of the design or analysis; F: assessment of outcome; G: was follow-up long enough for outcomes to occur; H: adequacy of follow up of cohorts.

**Supplementary Table 7.** Pooled relative risks (RR) and corresponding 95% confidence interval (95% CI) of selected risk factors for distal recurrence after surgical resection of non-small cell lung cancer among Asian populations (AP) and non Asian populations (NAP).

| **Distal recurrence risk factor** | **AP** | | **NAP** | | **p-value^a^** |
| --- | --- | --- | --- | --- | --- |
|  | **N. studies** | **Pooled RR (95% CI)** | **N. studies** | **Pooled RR (95% CI)** |  |
| **Sex** |  |  |  |  |  |
| Male vs female | 7 | 1.11 (0.81-1.52) | 5 | 1.26 (0.87-1.82) | 0.489 |
| **Age** |  |  |  |  |  |
| High vs low ^b^ | 3 | 0.90 (0.69-1.16) | 3 | 0.90 (0.72-1.13) | 0.980 |
| Continuous (1-year increase) | 1 | 1.01 (0.99-1.03) | 4 | 0.99 (0.96-1.02) | 0.095 |
| **Tobacco smoking** |  |  |  |  |  |
| Ever vs never | 6 | 1.19 (0.89-1.58) | 1 | 2.39 (1.36-4.19) | 0.029 |
| **Adjuvant chemotherapy** |  |  |  |  |  |
| Yes vs no | 9 | 1.09 (0.70-1-71) | 6 | 0.95 (0.71-1.26) | 0.531 |
| **Type of resection** |  |  |  |  |  |
| Pneumonectomy vs lobectomy | 1 | 1.44 (0.80-2.60) | 6 | 1.78 (0.78-4.06) | 0.630 |
| Segmentectomy/  wedge vs lobectomy | 2 | 1.10 (0.69-1.75) | 16 | 0.91 (0.81-1.03) | 0.445 |
| Sublobar NOS vs lobar resection | - | - | - | - | - |
| **Lobectomy** |  |  |  |  |  |
| VATS vs open | 2 | 0.82 (0.54-1.26) | 3 | 0.81 (0.64-1.04) | 0.965 |
| **Lymph node resected** |  |  |  |  |  |
| Number | 1 | 1.00 (0.98-1.03) | - | - | - |
| **Lymphovascular invasion** |  |  |  |  |  |
| Yes vs no | 12 | 3.41 (2.39-4.87) | 7 | 1.47 (1.20-1.79) | <0.001 |
| **(Visceral) pleural invasion** |  |  |  |  |  |
| Yes vs no | 8 | 1.91 (1.01-3.62) | 6 | 1.66 (1.13-2.44) | 0.714 |
| **Tumor grade** |  |  |  |  |  |
| Moderate/poor vs well | 3 | 4.72 (2.28-9.77) | 3 | 1.65 (1.21-2.25) | 0.009 |
| Poor vs well/moderate | 2 | 2.01 (1.15-3.51) | - | - | - |
| **Tumor histology** |  |  |  |  |  |
| Non adenocarcinoma vs adenocarcinoma | 7 | 0.98 (0.58-1.64) | 3 | 0.77 (0.71-0.83) | 0.372 |
| Non squamous cell vs squamous cell | 3 | 1.41 (0.96-2.08) | 5 | 1.34 (1.19-1.51) | 0.805 |
| **Tumor location** |  |  |  |  |  |
| Other location vs right upper lobe | 1 | 1.06 (0.73-1.54) | 2 | 1.32 (0.81-2.15) | 0.483 |
| Left lobe vs right lobe | 2 | 1.15 (0.85-1.56) | 1 | 1.47 (0.91-2.37) | 0.393 |
| **Tumor size** |  |  |  |  |  |
| Large vs small ^c^ | 4 | 2.05 (1.25-3.35) | 3 | 1.38 (1.22-1.56) | 0.125 |
| Continuous (1-cm increase) | - | - | 4 | 1.15 (1.10-1.21) | - |
| **Pathological stage** |  |  |  |  |  |
| II vs I | 3 | 2.66 (2.00-3.53) | 2 | 2.16 (1.48-3.16) | 0.395 |
| III-IV vs I | 2 | 2.17 (0.54-8.79) | 2 | 2.44 (2.21-2.69) | 0.870 |
| Ib-II vs Ia | 3 | 2.53 (1.74-3.67) | 2 | 1.64 (1.31-2.05) | 0.049 |
| III-IV vs Ia | 1 | 6.20 (2.12-18.14) | 2 | 2.01 (1.28-3.15) | 0.058 |
| **T stage** |  |  |  |  |  |
| T2 vs T1 | 1 | 1.91 (1.12-3.25) | 4 | 1.93 (1.10-3.38) | 0.983 |
| T3-T4 vs T1 | 1 | 2.66 (1.34-5.27) | 3 | 1.80 (1.39-2.33) | 0.294 |
| **N stage** |  |  |  |  |  |
| N1 vs N0 | 1 | 1.71 (1.16-2.53) | 4 | 1.67 (1.32-2.12) | 0.928 |
| N2 vs N0 | - | - | 2 | 1.92 (1.31-2.81) | - |
| N1-N2 vs N0 | 3 | 1.91 (1.22-3.00) | 5 | 1.79 (1.45-2.22) | 0.795 |

^a^ p-value for heterogeneity across AP and NAP. ^b^ Publications reporting age categories below 65-75 years or over 65-75 years. ^c^ Publications reporting tumor size categories below 1.5-4 cm or over 1.5-4 cm.

**Supplementary Table 8.** Pooled relative risks (RR) and corresponding 95% confidence interval (95% CI) of selected risk factors for overall recurrence after surgical resection of non-small cell lung cancer among Asian populations (AP) and non-Asian populations (NAP).

| **Overall recurrence risk factor** | **AP** | | **NAP** | | **p-value^a^** |
| --- | --- | --- | --- | --- | --- |
|  | **N. studies** | **Pooled RR (95% CI)** | **N. studies** | **Pooled RR (95% CI)** |  |
| **Sex** |  |  |  |  |  |
| Male vs female | 13 | 1.21 (0.98-1.49) | 8 | 1.25 (0.91-1.74) | 0.826 |
| **Age** |  |  |  |  |  |
| High vs low ^b^ | 7 | 1.23 (0.92-1.66) | 4 | 1.05 (0.92-1.21) | 0.213 |
| Continuous (1-year increase) | 5 | 1.02 (1.00-1.04) | 8 | 1.00 (0.98-1.01) | 0.024 |
| **Tobacco smoking** |  |  |  |  |  |
| Ever vs never | 8 | 1.30 (1.04-1.62) | 3 | 0.73 (0.40-1.32) | <0.001 |
| **Adjuvant chemotherapy** |  |  |  |  |  |
| Yes vs no | 6 | 1.15 (0.63-2.11) | 3 | 1.07 (0.29-3.90) | 0.845 |
| **Type of resection** |  |  |  |  |  |
| Pneumonectomy vs lobectomy | 2 | 1.21 (0.87-1.70) | 4 | 1.07 (0.90-1.28) | 0.512 |
| Segmentectomy/  wedge vs lobectomy | 6 | 0.96 (0.47-1.99) | 12 | 1.14 (1.03-1.26) | 0.658 |
| Sublobar NOS vs lobar resection | 2 | 1.25 (0.59-2.65) | 1 | 1.14 (0.78-1.66) | 0.827 |
| **Lobectomy** |  |  |  |  |  |
| VATS vs open | 1 | 0.81 (0.48-1.36) | 4 | 0.82 (0.68-0.99) | 0.958 |
| **Lymph node resected** |  |  |  |  |  |
| Number | 1 | 1 (0.98-1.02) | 4 | 1.02 (0.95-1.09) | 0.409 |
| **Lymphovascular invasion** |  |  |  |  |  |
| Yes vs no | 11 | 2.02 (1.64-2.51) | 5 | 1.39 (1.24-1.56) | 0.002 |
| **(Visceral) pleural invasion** |  |  |  |  |  |
| Yes vs no | 11 | 1.80 (1.11-2.90) | 5 | 1.41 (1.27-1.56) | 0.328 |
| **Tumor grade** |  |  |  |  |  |
| Moderate/poor vs well | 4 | 2.79 (1.81-4.29) | 3 | 1.47 (1.20-1.79) | 0.008 |
| Poor vs well/moderate | 1 | 2.62 (1.21-5.67) | 1 | 1.37 (1.03-1.82) | 0.122 |
| **Tumor histology** |  |  |  |  |  |
| Non adenocarcinoma vs adenocarcinoma | 8 | 1.03 (0.72-1.47) | 7 | 0.97 (0.94-1.00) | 0.754 |
| Non squamous cell vs squamous cell | 5 | 0.95 (0.79-1.15) | 3 | 1.34 (0.79-2.28) | 0.227 |
| **Tumor location** |  |  |  |  |  |
| Other location vs right upper lobe | 1 | 1.27 (0.90-1.79) | 2 | 1.00 (0.87-1.27) | 0.254 |
| Left lobe vs right lobe | 3 | 1 18 (0.84-1.66) | 1 | 1.28 (0.96-1.71) | 0.726 |
| **Tumor size** |  |  |  |  |  |
| Large vs small ^c^ | 7 | 1.60 (1.35-1.90) | 6 | 1.33 (1.18-1.49) | 0.080 |
| Continuous (1-cm increase) | 2 | 0.97 (0.53-1.80) | 3 | 1.04 (0.96-1.13) | 0.825 |
| **Pathological stage** |  |  |  |  |  |
| II vs I | 1 | 2.04 (1.31-3.18) | 1 | 1.66 (1.05-2.62) | 0.526 |
| III-IV vs I | - | - | 1 | 3.41 (1.96-5.94) | - |
| Ib-II vs Ia | 5 | 2.21 (1.66-2.93) | 2 | 1.77 (1.10-2.84) | 0.433 |
| III-IV vs Ia | - | - | 1 | 2.26 (2.17-2.36) | - |
| **T stage** |  |  |  |  |  |
| T2 vs T1 | - | - | 5 | 1.51 (1.20-1.90) | - |
| T3-T4 vs T1 | 1 | 1.74 (1.25-2.42) | 2 | 1.82 (1.45-2.28) | 0.830 |
| **N stage** |  |  |  |  |  |
| N1 vs N0 | 3 | 2.05 (1.58-2.67) | 3 | 1.54 (1.49-1.60) | 0.034 |
| N2 vs N0 | 2 | 2 58 (2.24-3.63) | 2 | 1.74 (1.66-1.84) | <0.001 |
| N1-N2 vs N0 | 6 | 2.71 (2.37-3.10) | 4 | 1.86 (1.44-2.40) | 0.010 |

^a^ p-value for heterogeneity across AP and NAP. ^b^ Publications reporting age categories below 65-75 years or over 65-75 years. ^c^ Publications reporting tumor size categories below 1.5-4 cm or over 1.5-4 cm.

**Supplementary Table 9.** Pooled relative risks (RR) and corresponding 95% confidence interval (95% CI) of selected risk factors for overall survival after surgical resection of non-small cell lung cancer among Asian populations (AP) and non Asian populations (NAP).

| **Overall survival risk factor** | **AP** | | **NAP** | | **p-value^a^** |
| --- | --- | --- | --- | --- | --- |
|  | **N. studies** | **Pooled RR (95% CI)** | **N. studies** | **Pooled RR (95% CI)** |  |
| **Sex** |  |  |  |  |  |
| Male vs female | 11 | 1.54 (1.21-1.92) | 5 | 1.41 (1.12; 1.78) | 0.484 |
| **Age** |  |  |  |  |  |
| High vs low ^b^ | 6 | 1.53 (0.98-2.39) | 5 | 1.30 (1.01-1.67) | 0.403 |
| Continuous (1-year increase) | 3 | 1.02 (0.92-1.12) | 8 | 1.02 (1.00-1.04) | 0.839 |
| **Tobacco smoking** |  |  |  |  |  |
| Ever vs never | 6 | 1.47 (1.11-1.96) | 1 | 0.98 (0.64-1.51) | 0.098 |
| **Adjuvant chemotherapy** |  |  |  |  |  |
| Yes vs no | 4 | 0.57 (0.25-1.33) | 6 | 0.46 (0.30-0.71) | 0.487 |
| **Type of resection** |  |  |  |  |  |
| Pneumonectomy vs lobectomy | 4 | 1.27 (1.06-1.53) | 4 | 1.36 (1.13-1.63) | 0.607 |
| Segmentectomy/  wedge vs lobectomy | 4 | 1.07 (0.58-2.01) | 12 | 1.27 (1.12-1.45) | 0.607 |
| Sublobar NOS vs lobar resection | 2 | 2.12 (1.06-4.24) | 3 | 1.48 (1.19-1.86) | 0.238 |
| **Lobectomy** |  |  |  |  |  |
| VATS vs open | 4 | 0.67 (0.47-0.96) | 3 | 0.73 (0.50-1.06) | 0.755 |
| **Lymph node resected** |  |  |  |  |  |
| Number | 1 | 0.99 (0.97-1.01) | 2 | 1.00 (0.90-1.11) | 0.785 |
| **Lymphovascular invasion** |  |  |  |  |  |
| Yes vs no | 8 | 1.82 (1.34-1.64) | 9 | 1.34 (1.24-1.45) | 0.014 |
| **(Visceral) pleural invasion** |  |  |  |  |  |
| Yes vs no | 7 | 1.29 (1.01-1.65) | 6 | 1.24 (1.11-1.39) | 0.801 |
| **Tumor grade** |  |  |  |  |  |
| Moderate/poor vs well | 3 | 3.98 (2.13-7.44) | 4 | 1.46 (1.21-1.76) | 0.003 |
| Poor vs well/moderate | - | - | - | - | - |
| **Tumor histology** |  |  |  |  |  |
| Non adenocarcinoma vs adenocarcinoma | 7 | 1.69 (1.13-2.54) | 5 | 1.06 (0.91-1.23) | 0.033 |
| Non squamous cell vs squamous cell | 4 | 0.92 (0.56-1.51) | 6 | 0.83 (0.73-0.98) | 0.712 |
| **Tumor location** |  |  |  |  |  |
| Other location vs right upper lobe | 1 | 1.24 (1.11-1.38) | 1 | 1.06 (0.89-1.26) | 0.128 |
| Left lobe vs right lobe | 2 | 1.04 (0.88-1.23) | 1 | 1.09 (0.93-1.27) | 0.685 |
| **Tumor size** |  |  |  |  |  |
| Large vs small ^c^ | 5 | 2.04 (1.31-3.20) | 5 | 1.55 (1.32-1.81) | 0.251 |
| Continuous (1-cm increase) | 2 | 1.37 (0.96-1.94) | 3 | 1.09 (0.98-1.21) | 0.227 |
| **Pathological stage** |  |  |  |  |  |
| II vs I | - | - | 1 | 1.37 (1.06-2.91) | - |
| III-IV vs I | - | - | 1 | 3.12 (1.73-5.62) | - |
| Ib-II vs Ia | 2 | 2.80 (0.80-9.81) | - | - | - |
| III-IV vs Ia | - | - | - | - | - |
| **T stage** |  |  |  |  |  |
| T2 vs T1 | - | - | 5 | 1.37 (1.09-1.73) | - |
| T3-T4 vs T1 | - | - | 2 | 2.17 (1.63-2.89) | - |
| **N stage** |  |  |  |  |  |
| N1 vs N0 | 2 | 2.31 (1.67-3.19) | 3 | 1.48 (1.43-1.54) | 0.008 |
| N2 vs N0 | 2 | 2.87 (2.12-3.87) | 2 | 1.86 (1.78-1.96) | 0.006 |
| N1-N2 vs N0 | 3 | 2.03 (1.19-3.47) | 4 | 1.68 (1.45-1.94) | 0.502 |

^a^ p-value for heterogeneity across AP and NAP. ^b^ Publications reporting age categories below 65-75 years or over 65-75 years. ^c^ Publications reporting tumor size categories below 1.5-4 cm or over 1.5-4 cm.

**Supplementary Table 10.** Locoregional and distal recurrence percentage by staging subgroup

| **Variable** | **Summary statistics** | **Early stage (N = 46)** | **Any stage (N = 30)** | **Advanced**  **stage (N = 12)** |
| --- | --- | --- | --- | --- |
| **Number of locoregional recurrences** | N | 46 (100.0%) | 27 (90.0%) | 11 (91.7%) |
|  | Mean ± SD | 33.9 ± 28.4 | 147.4 ± 233.1 | 29.8 ± 18.7 |
|  | Median (IQR) | 24.5 (16.0-36) | 61.0 (25.0-199) | 25.0 (16.0-41) |
|  | Min-Max | 11.0 - 128.0 | 11.0 - 1110.0 | 12.0 - 68.0 |
| **Number of patients** | N | 46 (100.0%) | 30 (100.0%) | 12 (100.0%) |
|  | Mean ± SD | 408.5 ± 309.5 | 1459.4 ± 2969.2 | 191.5 ± 103.4 |
|  | Median (IQR) | 325.0  (179.0-521) | 547.5  (189.0-1411) | 197.0  (93.5-267) |
|  | Min-Max | 66.0 - 1192.0 | 53.0 - 14578.0 | 54.0 - 377.0 |
| **% of locoregional recurrences** | N | 46 (100.0%) | 27 (90.0%) | 11 (91.7%) |
|  | Mean ± SD | 8.3 ± 5.4 | 9.5 ± 5.7 | 14.8 ± 6.7 |
|  | Median (IQR) | 6.7 (5.6-11) | 10.1 (4.0-12) | 14.9 (8.0-21) |
|  | Min-Max | 1.8 - 27.8 | 2.7 - 30.2 | 6.1 - 30.0 |
| **Number of distal recurrences** | N | 41 (89.1%) | 20 (66.7%) | 10 (83.3%) |
|  | Mean ± SD | 53.7 ± 49.3 | 319.2 ± 629.6 | 62.1 ± 45.1 |
|  | Median (IQR) | 34.0 (22.0-69) | 34.0 (28.0-253) | 53.0 (22.0-113) |
|  | Min-Max | 7.0 - 225.0 | 6.0 - 2238.0 | 14.0 - 116.0 |
| **Number of patients** | N | 46 (100.0%) | 28 (93.3%) | 12 (100.0%) |
|  | Mean ± SD | 408.5 ± 309.5 | 1481.5 ± 3075.1 | 191.5 ± 103.4 |
|  | Median (IQR) | 325.0  (179.0-521) | 530.5  (179.0-1481) | 197.0  (93.5-267) |
|  | Min-Max | 66.0 - 1192.0 | 53.0 - 14578.0 | 54.0 - 377.0 |
| **% of distal recurrences** | N | 41 (89.1%) | 20 (71.4%) | 10 (83.3%) |
|  | Mean ± SD | 12.4 ± 5.6 | 18.2 ± 7.0 | 30.7 ± 9.6 |
|  | Median (IQR) | 11.9 (7.3-17) | 15.4 (15.4-22) | 36.1 (23.3-37) |
|  | Min-Max | 3.8 - 36.4 | 2.8 - 43.4 | 13.3 - 40.7 |
| **Median follow-up (months)** | N | 29 (63.0%) | 24 (80.0%) | 10 (83.3%) |
|  | Mean ± SD | 54.2 ± 14.3 | 44.4 ± 17.2 | 45.1 ± 13.4 |
|  | Median (IQR) | 53.9 (44.6-63) | 35.0 (31.2-59) | 47.3 (33.0-54) |
|  | Min-Max | 31.0 - 80.4 | 20.0 - 88.9 | 24.0 - 66.0 |

**Supplementary Table 11.** Locoregional and distal recurrence percentage by recurrence definition

| **Variable** | **Summary statistics** | **Most Inclusive (N = 41)** | **Less Inclusive (N = 31)** | **Least Inclusive (N = 2)** | **NA (N = 14)** |
| --- | --- | --- | --- | --- | --- |
| **Number of locoregional recurrences** | N | 39 (95.1%) | 29 (93.5%) | 2 (100.0%) | 14 (100.0%) |
|  | Mean ± SD | 81.0 ± 193.9 | 55.3 ± 52.1 | 49.5 ± 36.1 | 71.9 ± 115.3 |
|  | Median (IQR) | 25.0 (16.0-54) | 37.0 (25.0-68) | 49.5 (24.0-75) | 20.5 (13.0-57) |
|  | Min-Max | 11.0 - 1110.0 | 12.0 - 220.0 | 24.0 - 75.0 | 11.0 - 401.0 |
| **Number of patients** | N | 41 (100.0%) | 31 (100.0%) | 2 (100.0%) | 14 (100.0%) |
|  | Mean ± SD | 881.8 ± 2592.8 | 601.7 ± 435.8 | 516.5 ± 378.3 | 645.1 ± 778.8 |
|  | Median (IQR) | 217.0  (152.0-433) | 468.0  (225.0-957) | 516.5  (249.0-784) | 317.5  (92.0-1023) |
|  | Min-Max | 53.0 - 14578.0 | 75.0 - 1568.0 | 249.0 - 784.0 | 54.0 - 2449.0 |
| **% of locoregional recurrences** | N | 39 (95.1%) | 29 (93.5%) | 2 (100.0%) | 14 (100.0%) |
|  | Mean ± SD | 8.8 ± 5.3 | 9.4 ± 5.7 | 9.6 ± 0.0 | 11.1 ± 7.4 |
|  | Median (IQR) | 6.7 (4.0-12) | 7.5 (5.7-14) | 9.6 (9.6-10) | 10.1 (3.2-13) |
|  | Min-Max | 2.7 - 30.2 | 1.8 - 23.9 | 9.6 - 9.6 | 2.1 - 27.2 |
| **Number of distal recurrences** | N | 33 (80.5%) | 23 (74.2%) | 2 (100.0%) | 13 (92.9%) |
|  | Mean ± SD | 161.3 ± 498.6 | 84.5 ± 68.3 | 90.0 ± 80.6 | 135.3 ± 204.2 |
|  | Median (IQR) | 31.0 (20.0-47) | 77.0 (28.0-116) | 90.0 (33.0-147) | 31.0 (22.0-126) |
|  | Min-Max | 6.0 - 2238.0 | 14.0 - 281.0 | 33.0 - 147.0 | 15.0 - 689.0 |
| **Number of patients** | N | 39 (95.1%) | 31 (100.0%) | 2 (100.0%) | 14 (100.0%) |
|  | Mean ± SD | 868.1 ± 2658.8 | 601.7 ± 435.8 | 516.5 ± 378.3 | 645.1 ± 778.8 |
|  | Median (IQR) | 217.0  (144.0-411) | 468.0  (225.0-957) | 516.5  (249.0-784) | 317.5  (92.0-1023) |
|  | Min-Max | 53.0 - 14578.0 | 75.0 - 1568.0 | 249.0 - 784.0 | 54.0 - 2449.0 |
| **% of distal recurrences** | N | 33 (84.6%) | 23 (74.2%) | 2 (100.0%) | 13 (92.9%) |
|  | Mean ± SD | 16.3 ± 5.2 | 15.9 ± 8.3 | 17.5 ± 2.4 | 19.6 ± 12.7 |
|  | Median (IQR) | 15.4 (15.4-22) | 13.7 (11.0-18) | 18.8 (18.8-19) | 17.3 (7.1-24) |
|  | Min-Max | 2.8 - 43.4 | 6.9 - 40.7 | 13.3 - 18.8 | 4.2 - 40.8 |
| **Median follow-up (months)** | N | 32 (78.0%) | 22 (71.0%) | 2 (100.0%) | 7 (50.0%) |
|  | Mean ± SD | 49.4 ± 14.7 | 45.1 ± 14.2 | 33.8 ± 3.9 | 63.9 ± 19.5 |
|  | Median (IQR) | 51.0 (33.8-60) | 43.4 (34.0-54) | 33.8 (31.0-37) | 60.0 (51.6-78) |
|  | Min-Max | 20.0 - 76.0 | 24.0 - 80.4 | 31.0 - 36.5 | 31.2 - 88.9 |

**Supplementary Table 12.** Logistic model for locoregional and distal recurrence percentage by staging subgroup

| **Variable** | **Category** | **OR (95% CI)** | **p-value** |
| --- | --- | --- | --- |
| **% of locoregional recurrence** | Any stage | 1.16 (1.09-1.24) | <0.001 |
|  | Advanced stage | 1.91 (1.68-2.18) | <0.001 |
|  | Early stage | 1 | - |
| **% of distal recurrence** | Any stage | 1.57 (1.49-1.65) | <0.001 |
|  | Advanced stage | 3.13 (2.82-3.47) | <0.001 |
|  | Early stage | 1 | - |

**Supplementary Table 13**. Logistic model for locoregional and distal recurrence percentage by recurrence definition group

| **Variable** | **Category** | **OR (95% CI)** | **p-value** |
| --- | --- | --- | --- |
| **% of locoregional recurrence** | NA | 1.30 (1.20-1.40) | <0.001 |
|  | Less Inclusive | 1.08 (1.01-1.15) | 0.024 |
|  | Least Inclusive | 1.10 (0.89-1.35) | 0.397 |
|  | Most Inclusive | 1 | - |
| **% of distal recurrence** | NA | 1.26 (1.19-1.34) | <0.001 |
|  | Less Inclusive | 0.97 (0.92-1.03) | 0.291 |
|  | Least Inclusive | 1.09 (0.92-1.28) | 0.314 |
|  | Most Inclusive | 1 | - |

**REFERENCES**

1. Cheng YD, Duan CJ, Dong S et al. Clinical controlled comparison between lobectomy and segmental resection for patients over 70 years of age with clinical stage I non-small cell lung cancer. Eur J Surg Oncol 2012; 38: 1149-1155.

2. Li N, Xu M, Li CF et al. Prognostic role of the ABO blood types in Chinese patients with curatively resected non-small cell lung cancer: a retrospective analysis of 1601 cases at a single cancer center. Chin J Cancer 2015; 34: 475-482.

3. Maurizi G, D'Andrilli A, Ciccone AM et al. Margin Distance Does Not Influence Recurrence and Survival After Wedge Resection for Lung Cancer. Ann Thorac Surg 2015; 100: 918-924; discussion 924-915.

4. Mazza F, Ferrari E, Maineri P et al. Pleural lavage cytology predicts recurrence and survival, even in early non-small cell lung cancer. Surg Today 2015; 45: 322-328.

5. Mohiuddin K, Haneuse S, Sofer T et al. Relationship between margin distance and local recurrence among patients undergoing wedge resection for small (≤2 cm) non-small cell lung cancer. J Thorac Cardiovasc Surg 2014; 147: 1169-1175; discussion 1175-1167.

6. Okada M, Sakamoto T, Yuki T et al. Selective mediastinal lymphadenectomy for clinico-surgical stage I non-small cell lung cancer. Ann Thorac Surg 2006; 81: 1028-1032.

7. Shimada Y, Ishii G, Hishida T et al. Extratumoral vascular invasion is a significant prognostic indicator and a predicting factor of distant metastasis in non-small cell lung cancer. J Thorac Oncol 2010; 5: 970-975.

8. Takahashi Y, Horio H, Hato T et al. Prognostic Significance of Preoperative Neutrophil-Lymphocyte Ratios in Patients with Stage I Non-small Cell Lung Cancer After Complete Resection. Ann Surg Oncol 2015; 22 Suppl 3: S1324-1331.

9. Takeda S, Maeda H, Koma M et al. Comparison of surgical results after pneumonectomy and sleeve lobectomy for non-small cell lung cancer: trends over time and 20-year institutional experience. Eur J Cardiothorac Surg 2006; 29: 276-280.

10. Varlotto JM, Recht A, Flickinger JC et al. Varying recurrence rates and risk factors associated with different definitions of local recurrence in patients with surgically resected, stage I nonsmall cell lung cancer. Cancer 2010; 116: 2390-2400.

11. Varlotto JM, Recht A, Flickinger JC et al. Factors associated with local and distant recurrence and survival in patients with resected nonsmall cell lung cancer. Cancer 2009; 115: 1059-1069.

12. Xia H, Sun Z, Deng L et al. Prognostic Significance of the Preoperative Lymphocyte to Monocyte Ratio in Patients With Stage I Non-Small Cell Lung Cancer Undergoing Complete Resection. Cancer Invest 2016; 34: 378-384.

13. Corsini EM, Weissferdt A, Pataer A et al. Pathological nodal disease defines survival outcomes in patients with lung cancer with tumour major pathological response following neoadjuvant chemotherapy. Eur J Cardiothorac Surg 2021; 59: 100-108.

14. Schreiner W, Dudek W, Rieker RJ et al. Major Pathologic Response after Induction Therapy Has a Long-Term Impact on Survival and Tumor Recurrence in Stage IIIA/B Locally Advanced NSCLC. Thorac Cardiovasc Surg 2020; 68: 639-645.

15. Okada M, Yamagishi H, Satake S et al. Survival related to lymph node involvement in lung cancer after sleeve lobectomy compared with pneumonectomy. J Thorac Cardiovasc Surg 2000; 119: 814-819.

16. Mineo TC, Ambrogi V, Corsaro V, Roselli M. Postoperative adjuvant therapy for stage IB non-small-cell lung cancer. Eur J Cardiothorac Surg 2001; 20: 378-384.

17. Maruyama R, Shoji F, Okamoto T et al. Prognostic value of visceral pleural invasion in resected non-small cell lung cancer diagnosed by using a jet stream of saline solution. J Thorac Cardiovasc Surg 2004; 127: 1587-1592.

18. Lardinois D, Suter H, Hakki H et al. Morbidity, survival, and site of recurrence after mediastinal lymph-node dissection versus systematic sampling after complete resection for non-small cell lung cancer. Ann Thorac Surg 2005; 80: 268-274; discussion 274-265.

19. El-Sherif A, Gooding WE, Santos R et al. Outcomes of sublobar resection versus lobectomy for stage I non-small cell lung cancer: a 13-year analysis. Ann Thorac Surg 2006; 82: 408-415; discussion 415-406.

20. Fujimoto T, Cassivi SD, Yang P et al. Completely resected N1 non-small cell lung cancer: factors affecting recurrence and long-term survival. J Thorac Cardiovasc Surg 2006; 132: 499-506.

21. Shiraishi T, Shirakusa T, Hiratsuka M et al. Video-assisted thoracoscopic surgery lobectomy for c-T1N0M0 primary lung cancer: its impact on locoregional control. Ann Thorac Surg 2006; 82: 1021-1026.

22. Schuchert MJ, Pettiford BL, Keeley S et al. Anatomic segmentectomy in the treatment of stage I non-small cell lung cancer. Ann Thorac Surg 2007; 84: 926-932; discussion 932-923.

23. Sienel W, Stremmel C, Kirschbaum A et al. Frequency of local recurrence following segmentectomy of stage IA non-small cell lung cancer is influenced by segment localisation and width of resection margins--implications for patient selection for segmentectomy. Eur J Cardiothorac Surg 2007; 31: 522-527; discussion 527-528.

24. Tsuchiya T, Akamine S, Muraoka M et al. Stage IA non-small cell lung cancer: vessel invasion is a poor prognostic factor and a new target of adjuvant chemotherapy. Lung Cancer 2007; 56: 341-348.

25. Shiraishi T, Hiratsuka M, Yoshinaga Y et al. Thoracoscopic lobectomy with systemic lymph node dissection for lymph node positive non-small cell lung cancer--is thoracoscopic lymph node dissection feasible? Thorac Cardiovasc Surg 2008; 56: 162-166.

26. Sienel W, Dango S, Kirschbaum A et al. Sublobar resections in stage IA non-small cell lung cancer: segmentectomies result in significantly better cancer-related survival than wedge resections. Eur J Cardiothorac Surg 2008; 33: 728-734.

27. Voltolini L, Rapicetta C, Luzzi L et al. Pattern of recurrence and survival of c-Ia NSCLC diagnosed by transpleural methods. J Cardiovasc Surg (Torino) 2008; 49: 697-702.

28. Bando T, Miyahara R, Sakai H et al. A follow-up report on a new method of segmental resection for small-sized early lung cancer. Lung Cancer 2009; 63: 58-62.

29. De Giacomo T, Di Stasio M, Diso D et al. Sub-lobar lung resection of peripheral T1N0M0 NSCLC does not affect local recurrence rate. Scand J Surg 2009; 98: 225-228.

30. Schuchert MJ, Pettiford BL, Pennathur A et al. Anatomic segmentectomy for stage I non-small-cell lung cancer: comparison of video-assisted thoracic surgery versus open approach. J Thorac Cardiovasc Surg 2009; 138: 1318-1325.e1311.

31. Shapiro M, Weiser TS, Wisnivesky JP et al. Thoracoscopic segmentectomy compares favorably with thoracoscopic lobectomy for patients with small stage I lung cancer. J Thorac Cardiovasc Surg 2009; 137: 1388-1393.

32. Kim K, Kim HK, Park JS et al. Video-assisted thoracic surgery lobectomy: single institutional experience with 704 cases. Ann Thorac Surg 2010; 89: S2118-2122.

33. Okami J, Ito Y, Higashiyama M et al. Sublobar resection provides an equivalent survival after lobectomy in elderly patients with early lung cancer. Ann Thorac Surg 2010; 90: 1651-1656.

34. Saynak M, Hubbs J, Nam J et al. Variability in defining T1N0 non-small cell lung cancer impacts locoregional failure and survival. Ann Thorac Surg 2010; 90: 1645-1649; discussion 1649-1650.

35. Shoji F, Haro A, Yoshida T et al. Prognostic significance of intratumoral blood vessel invasion in pathologic stage IA non-small cell lung cancer. Ann Thorac Surg 2010; 89: 864-869.

36. Darling GE, Allen MS, Decker PA et al. Randomized trial of mediastinal lymph node sampling versus complete lymphadenectomy during pulmonary resection in the patient with N0 or N1 (less than hilar) non-small cell carcinoma: results of the American College of Surgery Oncology Group Z0030 Trial. J Thorac Cardiovasc Surg 2011; 141: 662-670.

37. Koo HK, Jin SM, Lee CH et al. Factors associated with recurrence in patients with curatively resected stage I-II lung cancer. Lung Cancer 2011; 73: 222-229.

38. Park JS, Kim K, Choi MS et al. Video-Assisted Thoracic Surgery (VATS) Lobectomy for Pathologic Stage I Non-Small Cell Lung Cancer: A Comparative Study with Thoracotomy Lobectomy. Korean J Thorac Cardiovasc Surg 2011; 44: 32-38.

39. Pepek JM, Chino JP, Marks LB et al. How well does the new lung cancer staging system predict for local/regional recurrence after surgery?: A comparison of the TNM 6 and 7 systems. J Thorac Oncol 2011; 6: 757-761.

40. Saynak M, Veeramachaneni NK, Hubbs JL et al. Local failure after complete resection of N0-1 non-small cell lung cancer. Lung Cancer 2011; 71: 156-165.

41. Schuchert MJ, Schumacher L, Kilic A et al. Impact of angiolymphatic and pleural invasion on surgical outcomes for stage I non-small cell lung cancer. Ann Thorac Surg 2011; 91: 1059-1065; discussion 1065.

42. Varlotto JM, Medford-Davis LN, Recht A et al. Failure rates and patterns of recurrence in patients with resected N1 non-small-cell lung cancer. Int J Radiat Oncol Biol Phys 2011; 81: 353-359.

43. Wolf AS, Richards WG, Jaklitsch MT et al. Lobectomy versus sublobar resection for small (2 cm or less) non-small cell lung cancers. Ann Thorac Surg 2011; 92: 1819-1823; discussion 1824-1815.

44. Xie L, Saynak M, Veeramachaneni NK et al. Non-small cell lung cancer: prognostic importance of positive FDG PET findings in the mediastinum for patients with N0-N1 disease at pathologic analysis. Radiology 2011; 261: 226-234.

45. Carr SR, Schuchert MJ, Pennathur A et al. Impact of tumor size on outcomes after anatomic lung resection for stage 1A non-small cell lung cancer based on the current staging system. J Thorac Cardiovasc Surg 2012; 143: 390-397.

46. Higgins KA, Chino JP, Ready N et al. Lymphovascular invasion in non-small-cell lung cancer: implications for staging and adjuvant therapy. J Thorac Oncol 2012; 7: 1141-1147.

47. Higgins KA, Chino JP, Berry M et al. Local failure in resected N1 lung cancer: implications for adjuvant therapy. Int J Radiat Oncol Biol Phys 2012; 83: 727-733.

48. Hung JJ, Jeng WJ, Hsu WH et al. Predictors of death, local recurrence, and distant metastasis in completely resected pathological stage-I non-small-cell lung cancer. J Thorac Oncol 2012; 7: 1115-1123.

49. Hung JJ, Jeng WJ, Hsu WH et al. Prognostic significance of the extent of visceral pleural invasion in completely resected node-negative non-small cell lung cancer. Chest 2012; 142: 141-150.

50. Stefani A, Nesci J, Casali C, Morandi U. Wedge resection versus lobectomy for T1N0 non-small cell lung cancer. Minerva Chir 2012; 67: 489-498.

51. Varlotto J, Medford-Davis LN, Recht A et al. Confirmation of the role of diabetes in the local recurrence of surgically resected non-small cell lung cancer. Lung Cancer 2012; 75: 381-390.

52. Choi PJ, Jeong SS, Yoon SS. Prognosis of recurrence after complete resection in early-stage non-small cell lung cancer. Korean J Thorac Cardiovasc Surg 2013; 46: 449-456.

53. Fan C, Gao S, Hui Z et al. Risk factors for locoregional recurrence in patients with resected N1 non-small cell lung cancer: a retrospective study to identify patterns of failure and implications for adjuvant radiotherapy. Radiat Oncol 2013; 8: 286.

54. Kelsey CR, Higgins KA, Peterson BL et al. Local recurrence after surgery for non-small cell lung cancer: a recursive partitioning analysis of multi-institutional data. J Thorac Cardiovasc Surg 2013; 146: 768-773.e761.

55. Koike T, Koike T, Yoshiya K et al. Risk factor analysis of locoregional recurrence after sublobar resection in patients with clinical stage IA non-small cell lung cancer. J Thorac Cardiovasc Surg 2013; 146: 372-378.

56. Lopez Guerra JL, Gomez DR, Lin SH et al. Risk factors for local and regional recurrence in patients with resected N0-N1 non-small-cell lung cancer, with implications for patient selection for adjuvant radiation therapy. Ann Oncol 2013; 24: 67-74.

57. Tandberg DJ, Gee NG, Chino JP et al. Are discordant positron emission tomography and pathological assessments of the mediastinum in non-small cell lung cancer significant? J Thorac Cardiovasc Surg 2013; 146: 796-801.

58. Varlotto JM, Medford-Davis LN, Recht A et al. Identification of stage I non-small cell lung cancer patients at high risk for local recurrence following sublobar resection. Chest 2013; 143: 1365-1377.

59. Washington I, Chino JP, Marks LB et al. Diabetes mellitus: A significant co-morbidity in the setting of lung cancer? Thorac Cancer 2013; 4: 123-130.

60. Yanagawa N, Shiono S, Abiko M et al. Prognostic impact and initial recurrence site of lymphovascular and visceral pleural invasion in surgically resected stage I non-small-cell lung carcinoma. Eur J Cardiothorac Surg 2013; 44: e200-206.

61. Chen YY, Huang TW, Tsai WC et al. Risk factors of postoperative recurrences in patients with clinical stage I NSCLC. World J Surg Oncol 2014; 12: 10.

62. Ichinose J, Murakawa T, Hino H et al. Prognostic impact of the current Japanese nodal classification on outcomes in resected non-small cell lung cancer. Chest 2014; 146: 644-649.

63. Landreneau RJ, Normolle DP, Christie NA et al. Recurrence and survival outcomes after anatomic segmentectomy versus lobectomy for clinical stage I non-small-cell lung cancer: a propensity-matched analysis. J Clin Oncol 2014; 32: 2449-2455.

64. Peters S, Weder W, Dafni U et al. Lungscape: resected non-small-cell lung cancer outcome by clinical and pathological parameters. J Thorac Oncol 2014; 9: 1675-1684.

65. Rotolo F, Dunant A, Le Chevalier T et al. Adjuvant cisplatin-based chemotherapy in nonsmall-cell lung cancer: new insights into the effect on failure type via a multistate approach. Ann Oncol 2014; 25: 2162-2166.

66. Su S, Scott WJ, Allen MS et al. Patterns of survival and recurrence after surgical treatment of early stage non-small cell lung carcinoma in the ACOSOG Z0030 (ALLIANCE) trial. J Thorac Cardiovasc Surg 2014; 147: 747-752: Discussion 752-743.

67. Lee SH, Jo EJ, Eom JS et al. Predictors of Recurrence after Curative Resection in Patients with Early-Stage Non-Small Cell Lung Cancer. Tuberc Respir Dis (Seoul) 2015; 78: 341-348.

68. Ogawa H, Uchino K, Tanaka Y et al. Efficacy of adjuvant chemotherapy for lung adenocarcinoma patients with positive pleural lavage cytology findings. Interact Cardiovasc Thorac Surg 2015; 21: 34-39.

69. Varlotto JM, Yao AN, DeCamp MM et al. Nodal stage of surgically resected non-small cell lung cancer and its effect on recurrence patterns and overall survival. Int J Radiat Oncol Biol Phys 2015; 91: 765-773.

70. Altorki NK, Kamel MK, Narula N et al. Anatomical Segmentectomy and Wedge Resections Are Associated with Comparable Outcomes for Patients with Small cT1N0 Non-Small Cell Lung Cancer. J Thorac Oncol 2016; 11: 1984-1992.

71. Billè A, Ahmad U, Woo KM et al. Detection of Recurrence Patterns After Wedge Resection for Early Stage Lung Cancer: Rationale for Radiologic Follow-Up. Ann Thorac Surg 2016; 102: 1067-1073.

72. Borghetti P, Barbera F, Bonù ML et al. Resected pN1 non-small cell lung cancer: recurrence patterns and nodal risk factors may suggest selection criteria for post-operative radiotherapy. Radiol Med 2016; 121: 696-703.

73. Dziedzic DA, Rudzinski P, Langfort R, Orlowski T. Risk Factors for Local and Distant Recurrence After Surgical Treatment in Patients With Non-Small-Cell Lung Cancer. Clin Lung Cancer 2016; 17: e157-e167.

74. Kim IH, Lee IH, Lee JE et al. Prognostic Impact of Multiple Clinicopathologic Risk Factors and c-MET Overexpression in Patients Who Have Undergone Resection of Stage IB Non-Small-Cell Lung Cancer. Clin Lung Cancer 2016; 17: e31-e43.

75. Kodama K, Higashiyama M, Okami J et al. Oncologic Outcomes of Segmentectomy Versus Lobectomy for Clinical T1a N0 M0 Non-Small Cell Lung Cancer. Ann Thorac Surg 2016; 101: 504-511.

76. Nishio W, Yoshimura M, Maniwa Y et al. Re-Assessment of Intentional Extended Segmentectomy for Clinical T1aN0 Non-Small Cell Lung Cancer. Ann Thorac Surg 2016; 102: 1702-1710.

77. Wong ML, McMurry TL, Stukenborg GJ et al. Impact of age and comorbidity on treatment of non-small cell lung cancer recurrence following complete resection: A nationally representative cohort study. Lung Cancer 2016; 102: 108-117.

78. Stiles BM, Kamel MK, Nasar A et al. The importance of lymph node dissection accompanying wedge resection for clinical stage IA lung cancer. Eur J Cardiothorac Surg 2017; 51: 511-517.

79. Tian D, Pei Y, Zheng Q et al. Effect of visceral pleural invasion on the prognosis of patients with lymph node negative non-small cell lung cancer. Thorac Cancer 2017; 8: 97-105.

80. Aprile V, Bertoglio P, Dini P et al. Is left upper lobectomy always worthwhile for early stage lung cancer? A comparison between left upper lobectomy, trisegmentectomy, and lingulectomy. J Surg Oncol 2018; 117: 618-624.

81. Brandt WS, Bouabdallah I, Tan KS et al. Factors associated with distant recurrence following R0 lobectomy for pN0 lung adenocarcinoma. J Thorac Cardiovasc Surg 2018; 155: 1212-1224.e1213.

82. Isaka M, Kojima H, Takahashi S et al. Risk factors for local recurrence after lobectomy and lymph node dissection in patients with non-small cell lung cancer: Implications for adjuvant therapy. Lung Cancer 2018; 115: 28-33.

83. Judy GD, Kaidar-Person O, Deal A et al. The Persistent Problem of Local/Regional Failure After Surgical Intervention for Early-Stage Lung Cancer. Ann Thorac Surg 2018; 106: 382-389.

84. Sung SY, Kwak YK, Lee SW et al. Lymphovascular Invasion Increases the Risk of Nodal and Distant Recurrence in Node-Negative Stage I-IIA Non-Small-Cell Lung Cancer. Oncology 2018; 95: 156-162.

85. Zhong C, Sakurai H, Wei S et al. Sublobar resections for small-sized stage Ia lung adenocarcinoma: a Sino-Japanese multicenter study. J Thorac Dis 2018; 10: 991-998.

86. Matsuura N, Go T, Fujiwara A et al. Lymphatic invasion is a cause of local recurrence after wedge resection of primary lung cancer. Gen Thorac Cardiovasc Surg 2019; 67: 861-866.

87. Schuchert MJ, Normolle DP, Awais O et al. Factors influencing recurrence following anatomic lung resection for clinical stage I non-small cell lung cancer. Lung Cancer 2019; 128: 145-151.

88. Kneuertz PJ, D'Souza DM, Richardson M et al. Long-Term Oncologic Outcomes After Robotic Lobectomy for Early-stage Non-Small-cell Lung Cancer Versus Video-assisted Thoracoscopic and Open Thoracotomy Approach. Clin Lung Cancer 2020; 21: 214-224.e212.

89. Ma M, He F, Lv X et al. Feasibility and effectiveness of thoracoscopic pulmonary segmentectomy for non-small cell lung cancer. Medicine (Baltimore) 2020; 99: e18959.

90. Mun M, Nakao M, Matsuura Y et al. Oncological outcomes after lobe-specific mediastinal lymph node dissection via multiport video-assisted thoracoscopic surgery. Eur J Cardiothorac Surg 2020; 58: i92-i99.

91. Nakagawa K, Yoshida Y, Yotsukura M, Watanabe SI. Pattern of recurrence of pN2 non-small-cell lung cancer: should postoperative radiotherapy be reconsidered? Eur J Cardiothorac Surg 2020.

92. Shimizu R, Kinoshita T, Sasaki N et al. Clinicopathological Factors Related to Recurrence Patterns of Resected Non-Small Cell Lung Cancer. J Clin Med 2020; 9.

93. Vaghjiani RG, Takahashi Y, Eguchi T et al. Tumor Spread Through Air Spaces Is a Predictor of Occult Lymph Node Metastasis in Clinical Stage IA Lung Adenocarcinoma. J Thorac Oncol 2020; 15: 792-802.

94. Yoshida Y, Yotsukura M, Nakagawa K et al. Surgical Results in Pathological N1 Nonsmall Cell Lung Cancer. Thorac Cardiovasc Surg 2020.

95. Sato T, Shimada Y, Mimae T et al. The impact of pathological lymph node metastasis with lymphatic invasion on the survival of patients with clinically node-negative non-small cell lung cancer: A multicenter study. Lung Cancer 2021; 158: 9-14.

96. Hu J, Chen Y, Zhu X et al. Surgical choice of non-small cell lung cancer with unexpected pleural dissemination intraoperatively. BMC Cancer 2021; 21: 445.

97. Schiavon M, Comacchio GM, Mammana M et al. Lobectomy With Artery Reconstruction and Pneumonectomy for Non-Small Cell Lung Cancer: A Propensity Score Weighting Study. Ann Thorac Surg 2021.

98. Jiang G, Huang J, Cui T et al. A biomarker-based prediction model for risk of locoregional recurrence in pathologic stage IIIA-N2 non-small cell lung cancer. Int J Clin Exp Pathol 2020; 13: 3060-3082.

99. Sugita Y, Kinoshita T, Shima T et al. Lymphatic permeation and vascular invasion should not be integrated as lymphovascular invasion in lung adenocarcinoma. Gen Thorac Cardiovasc Surg 2021; 69: 1070-1078.

100. Dolan DP, White A, Mazzola E et al. Outcomes of superior segmentectomy versus lower lobectomy for superior segment Stage I non-small-cell lung cancer are equivalent: An analysis of 196 patients at a single, high volume institution. J Surg Oncol 2021; 123: 570-578.

**Supplementary Figure 1.** Flow-chart of study selection.

**Supplementary Figure 2.** Funnel plots of studies on the association between selected risk factors and local/locoregional recurrence after surgical resection of non-small cell lung cancer.

LVI: lymphovascular invasion; VPI: Visceral pleural invasion.


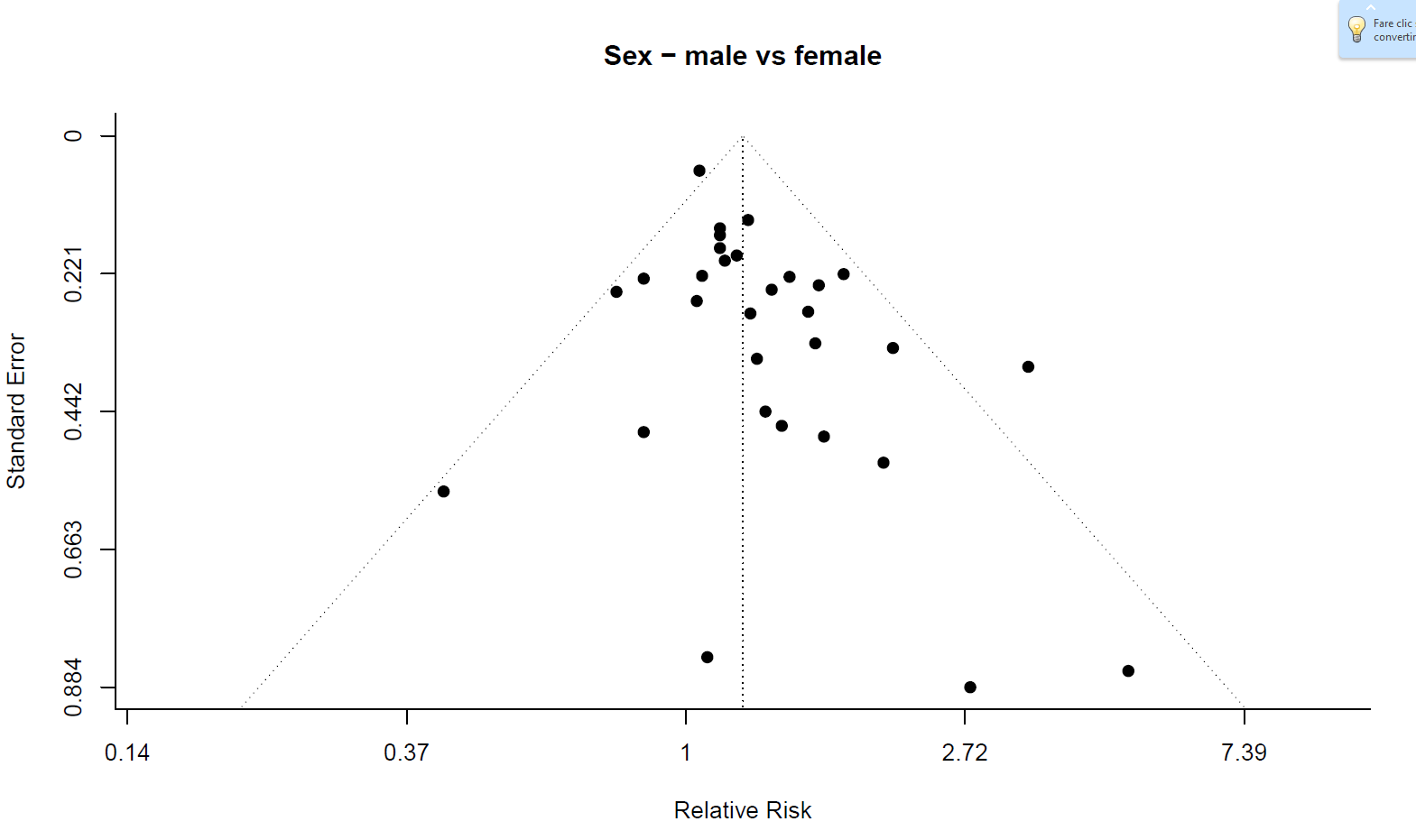


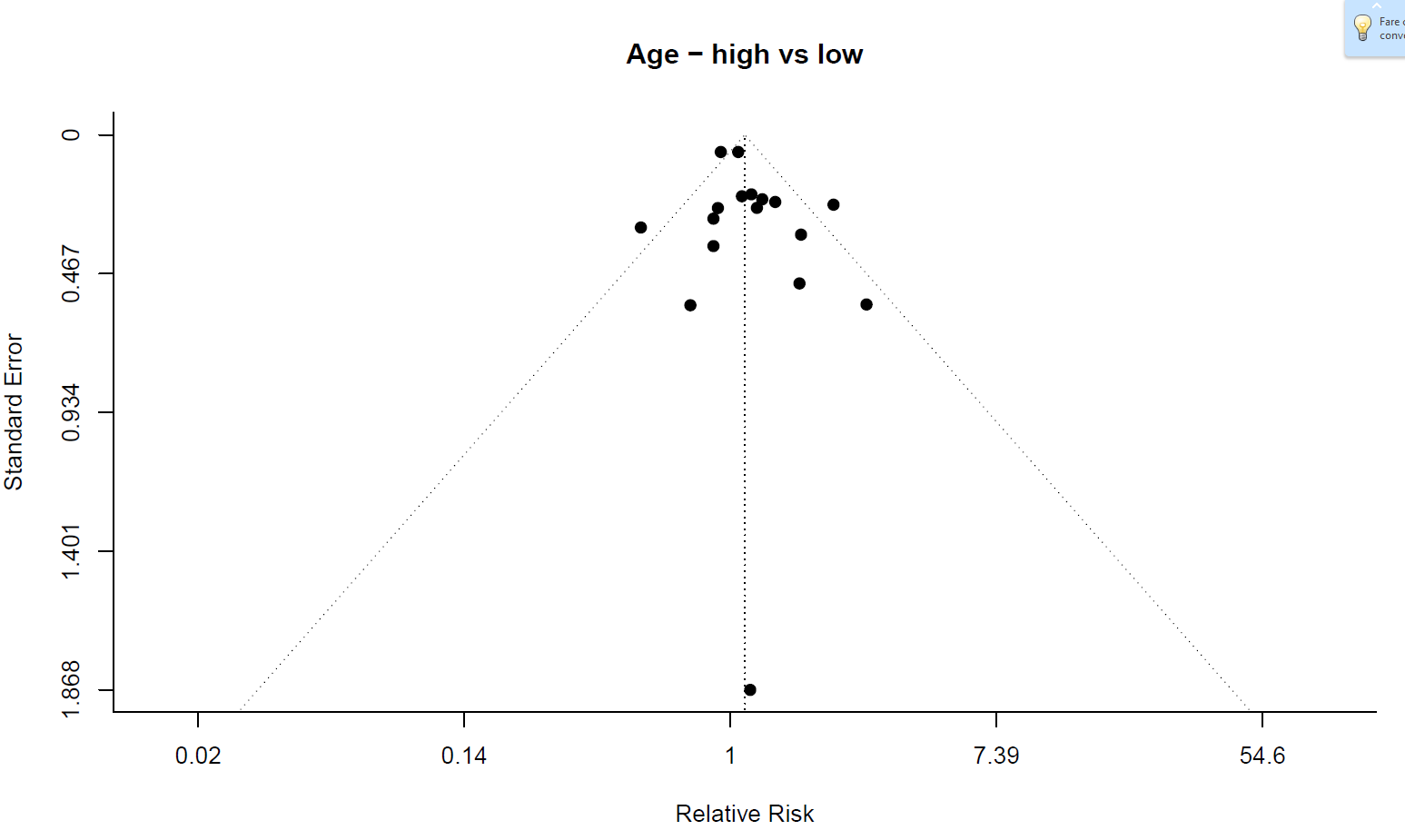


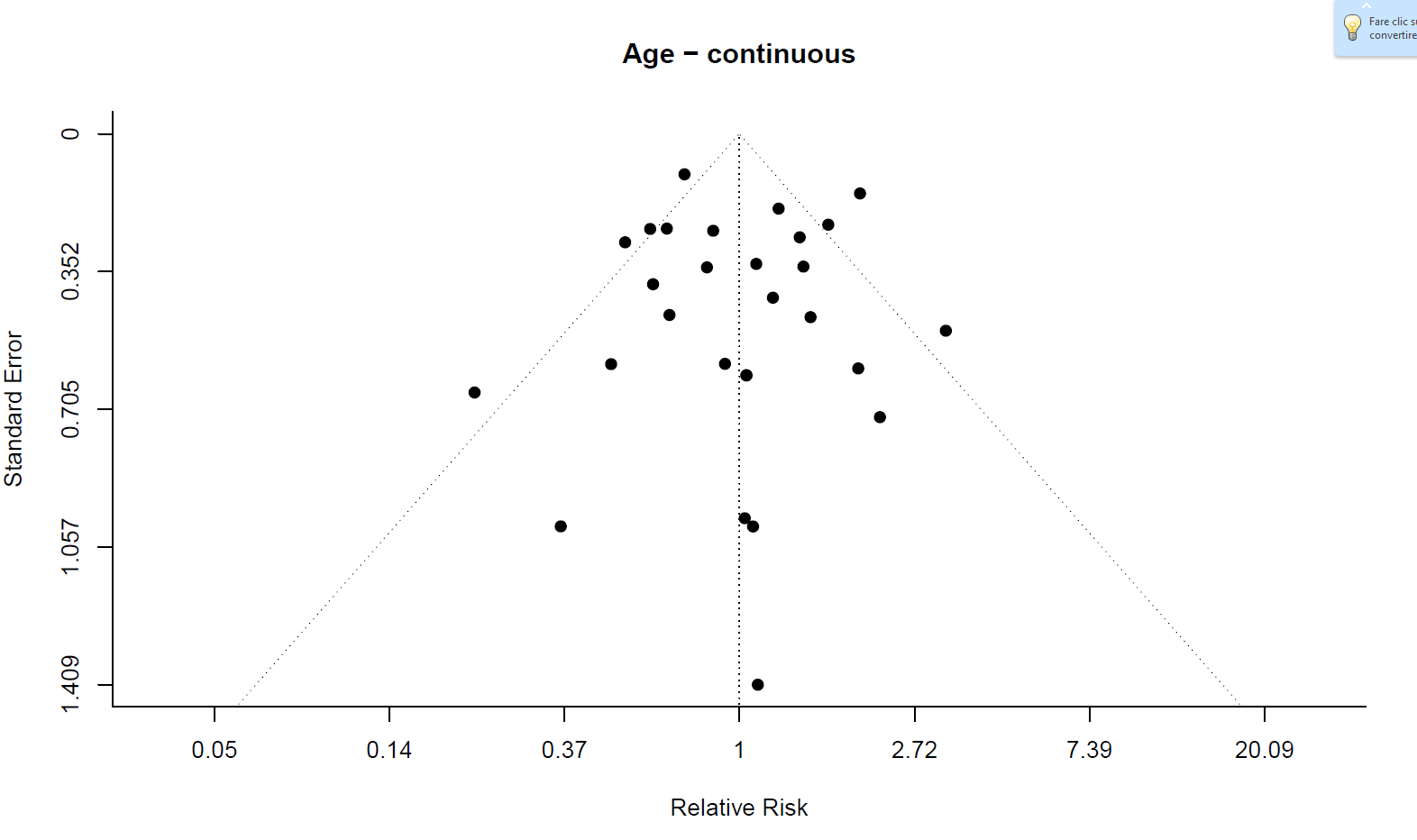


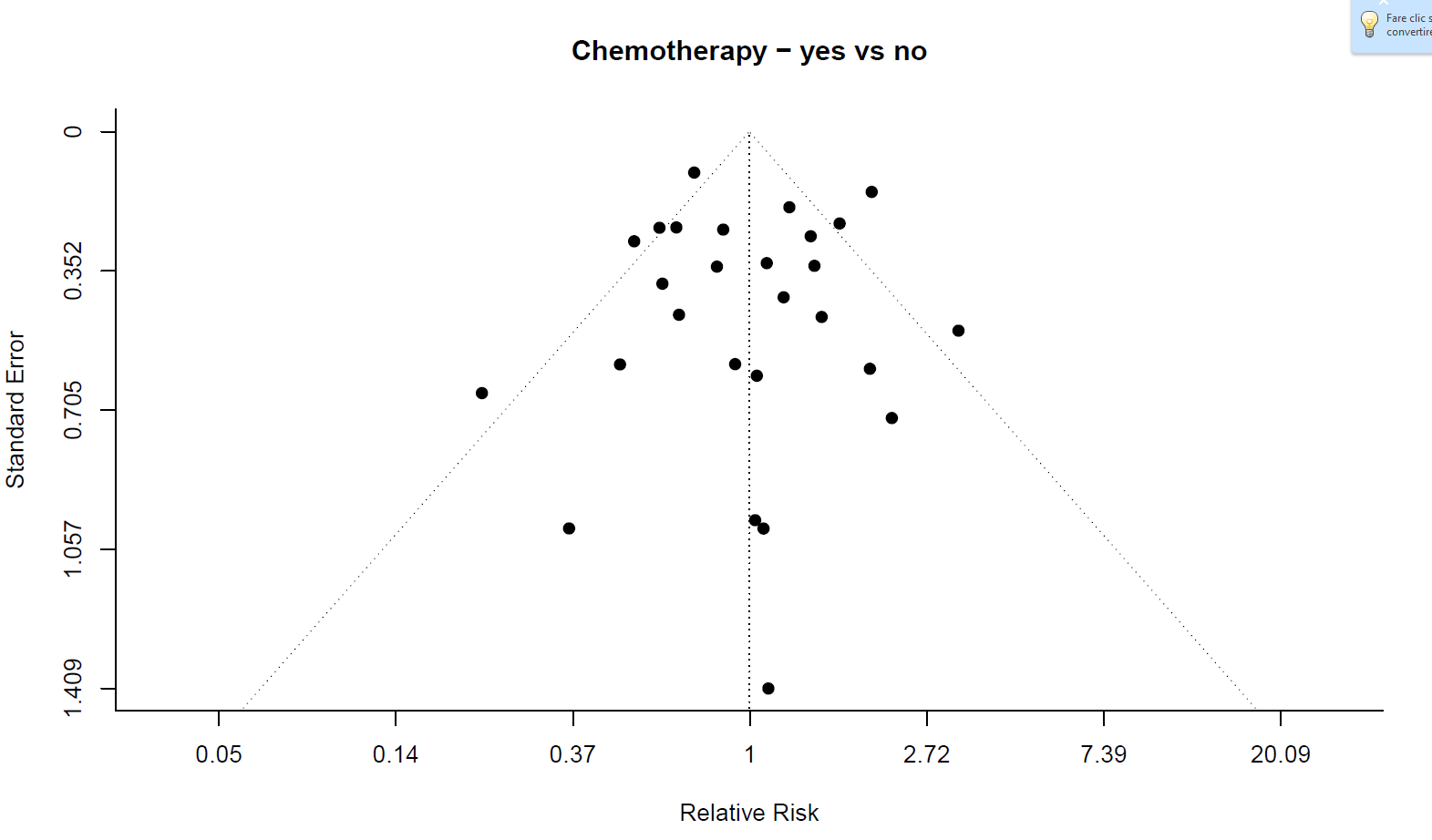


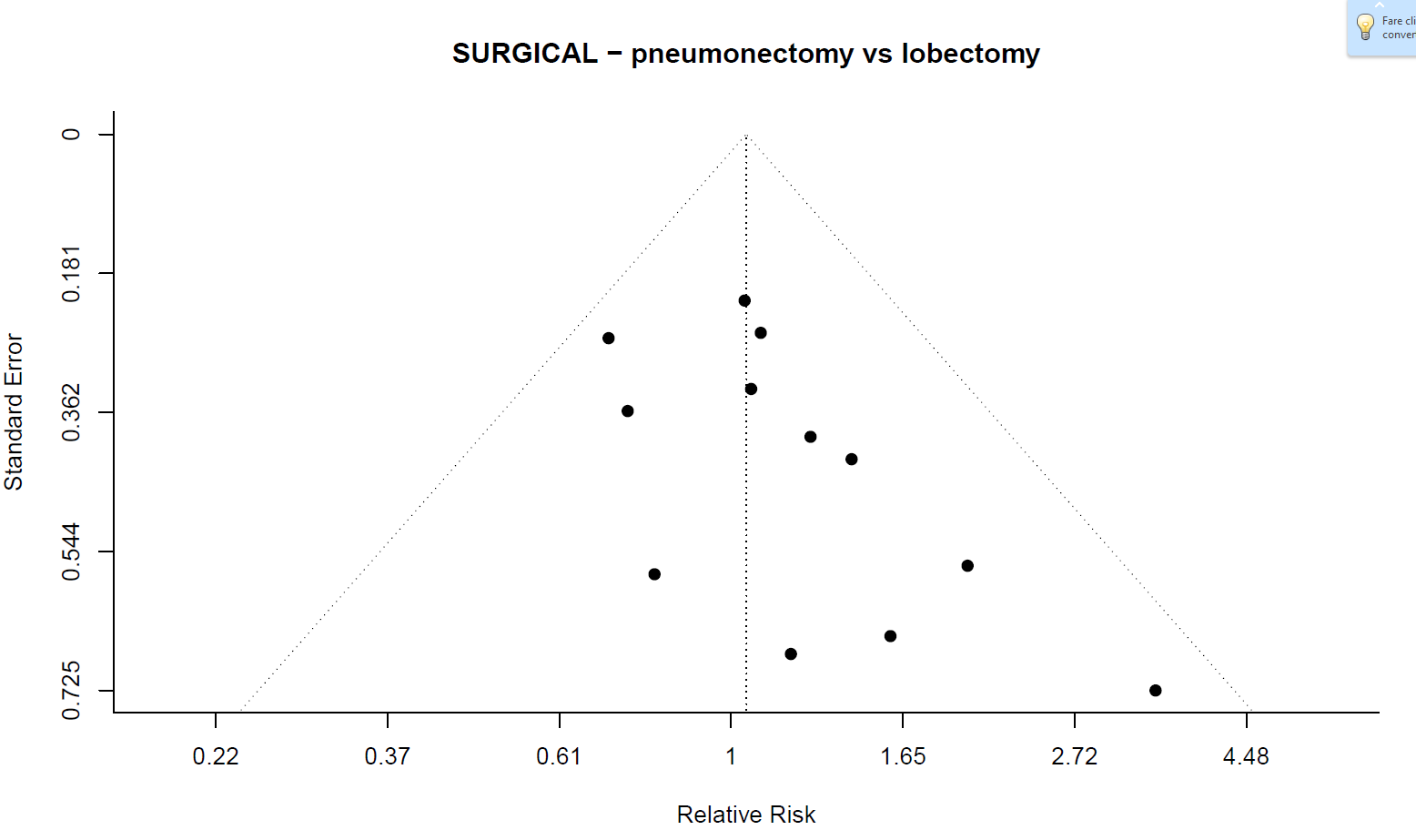


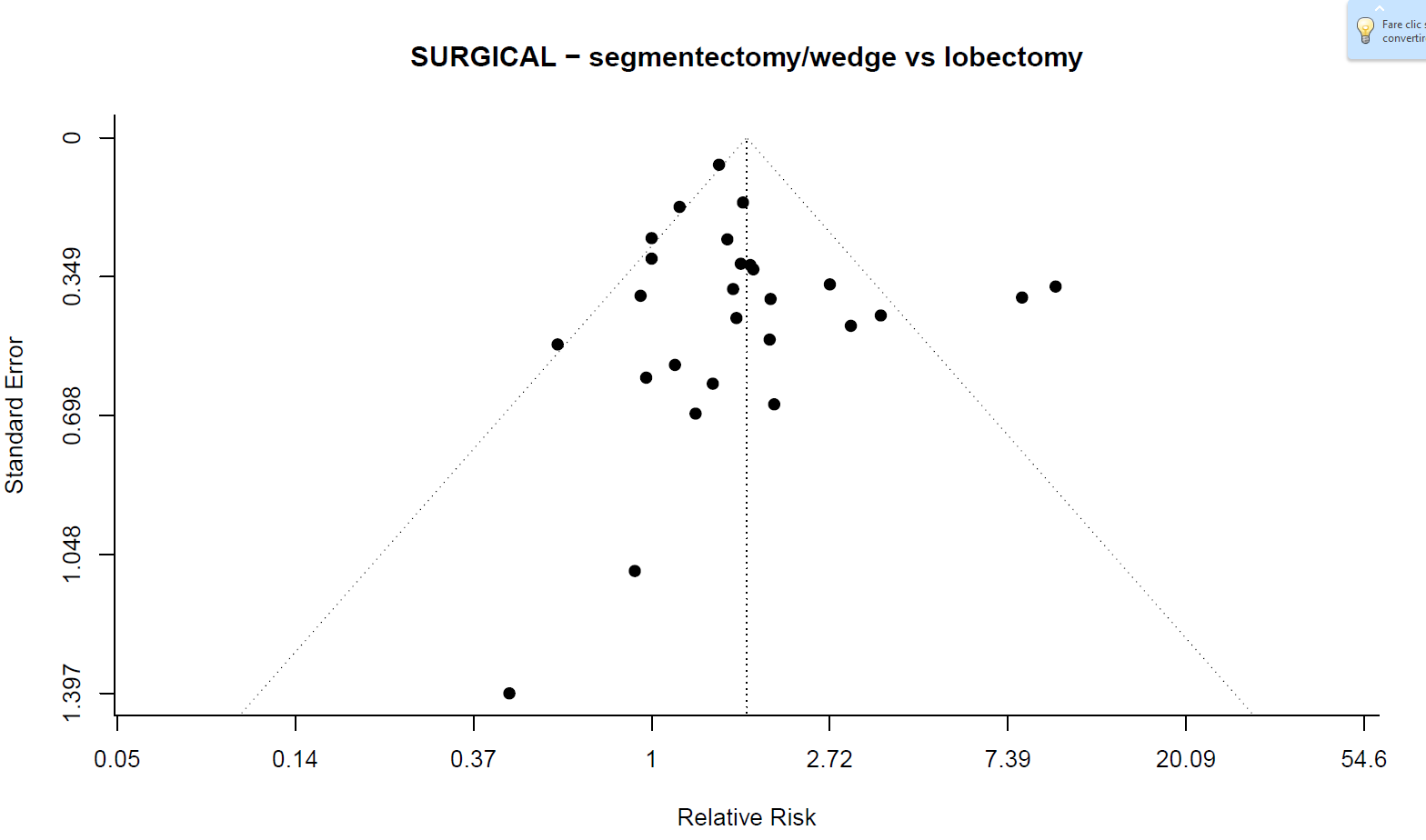


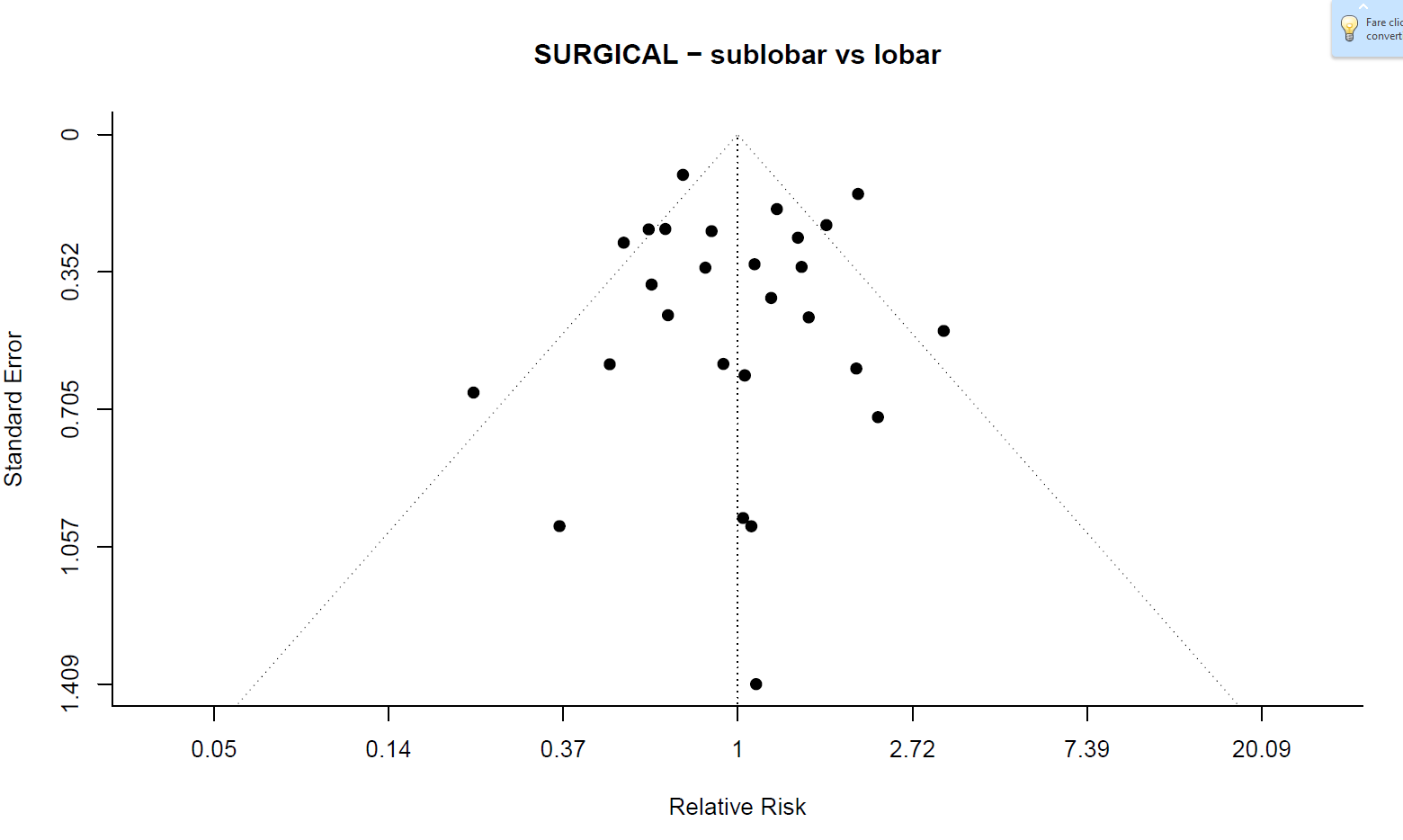


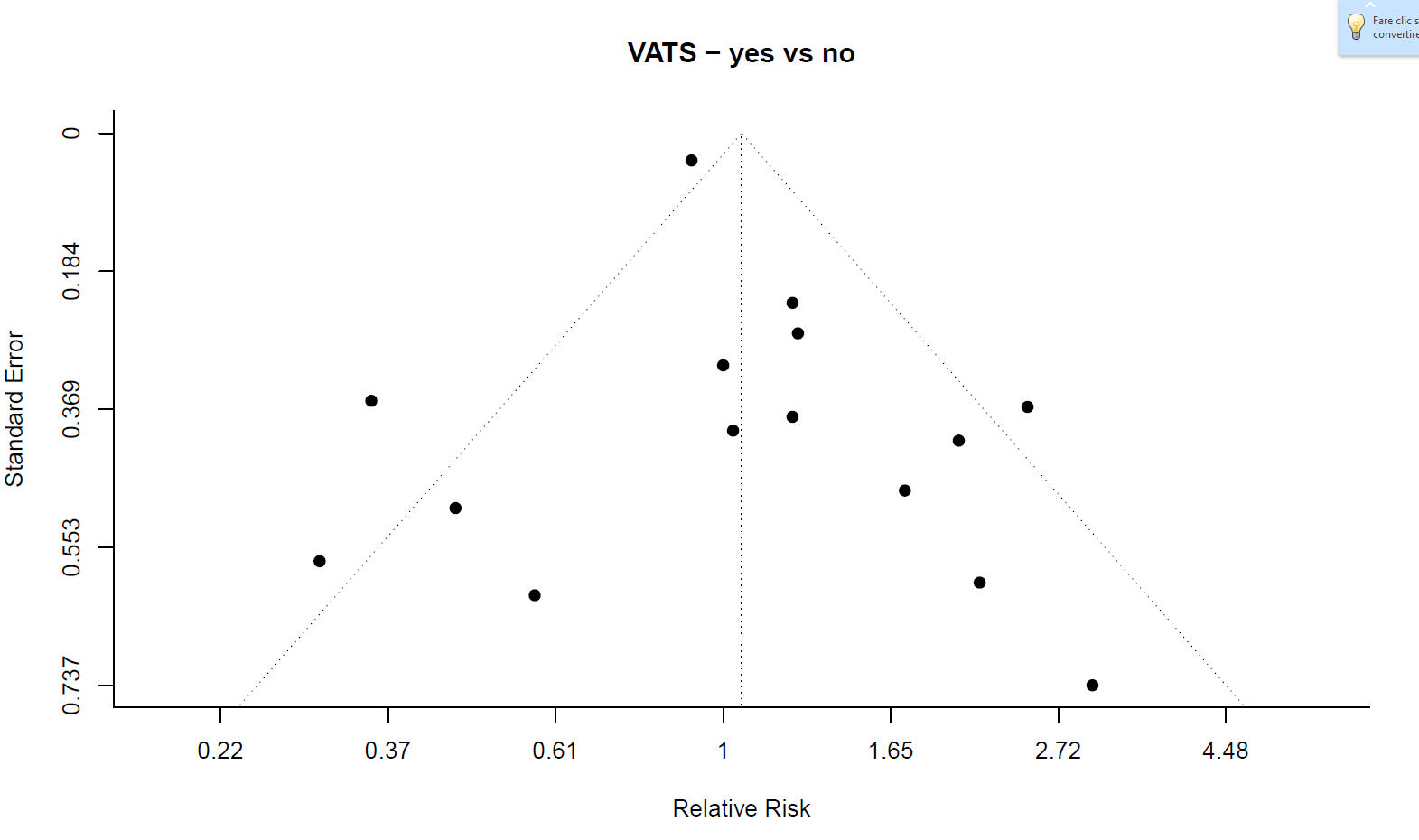


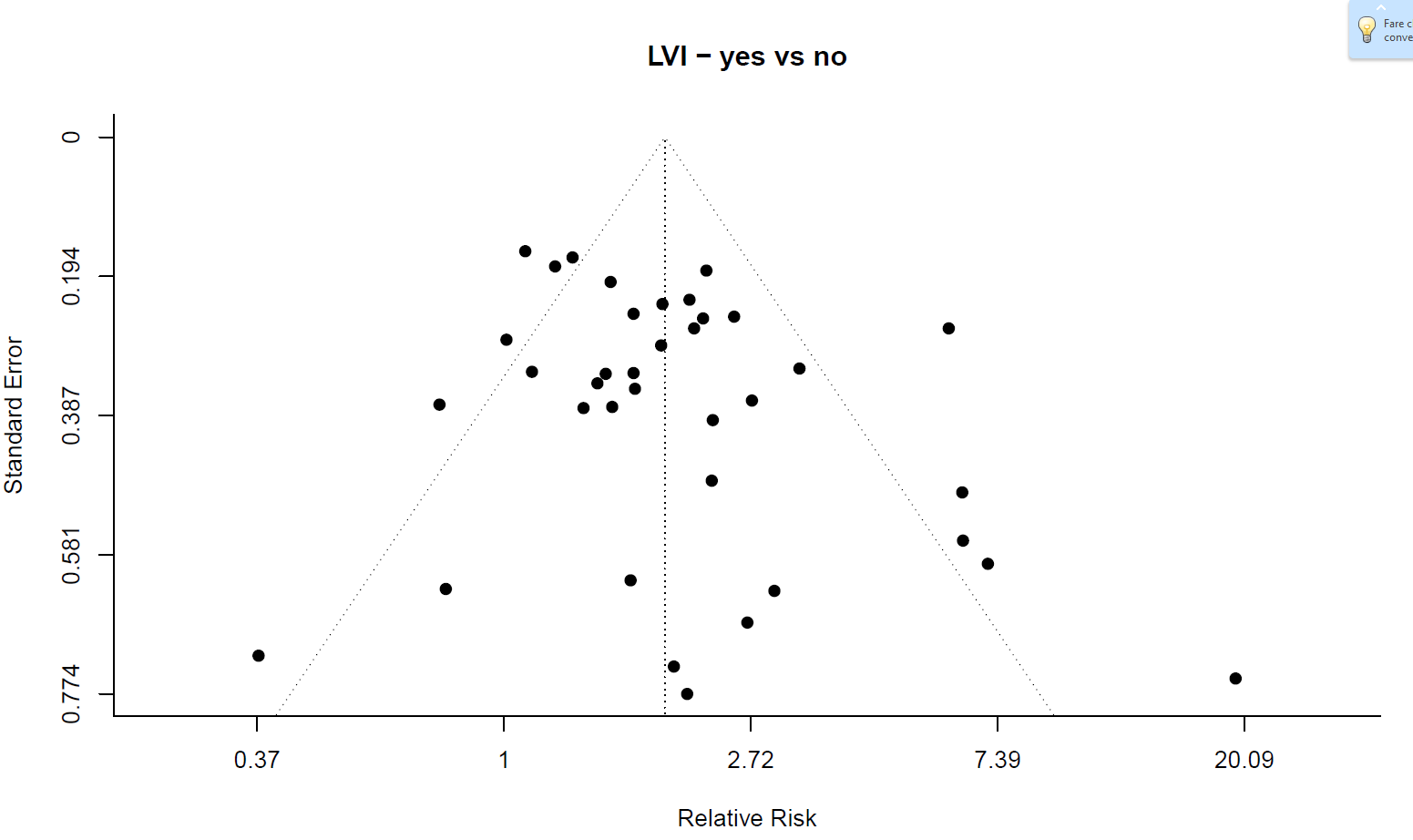


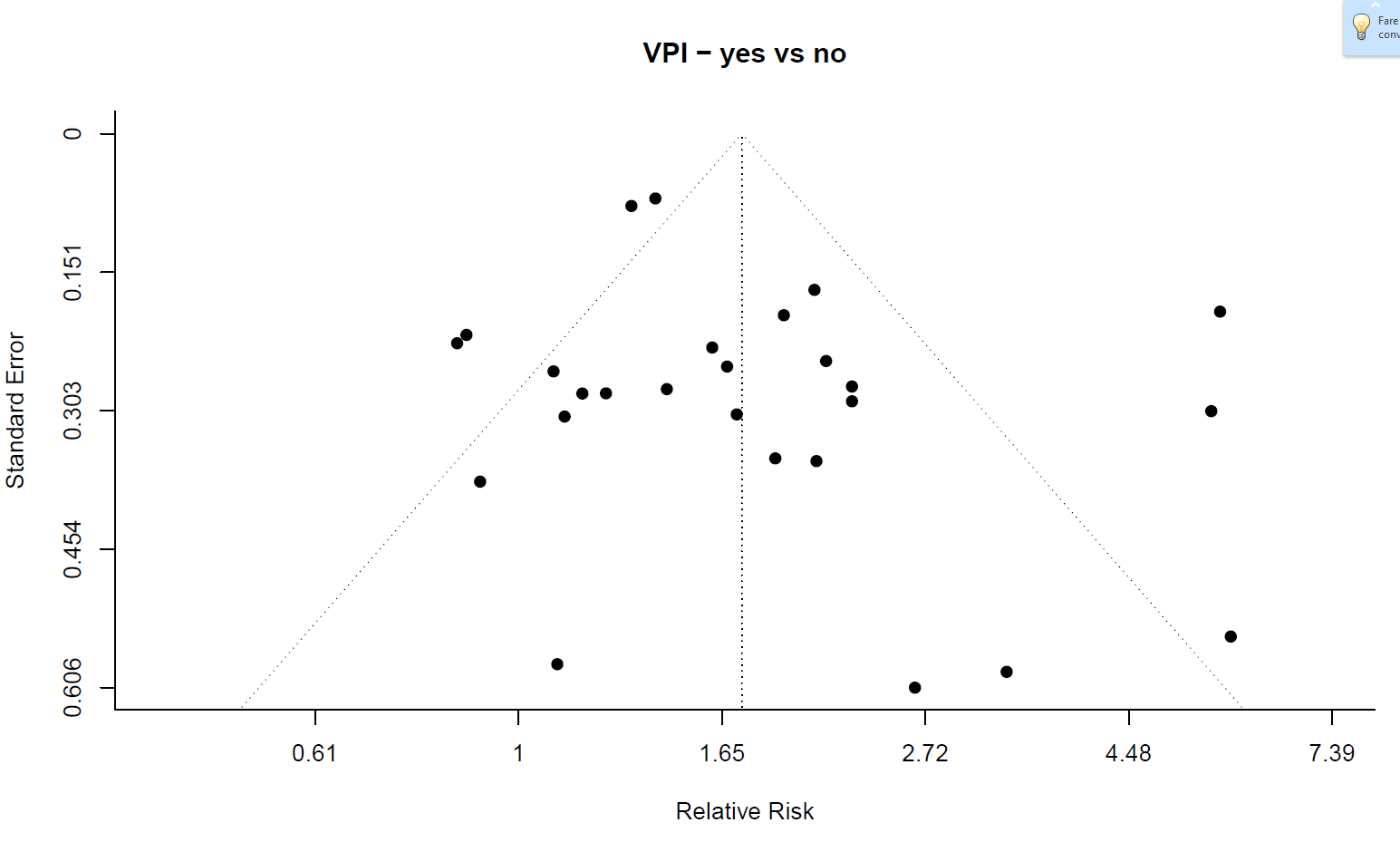


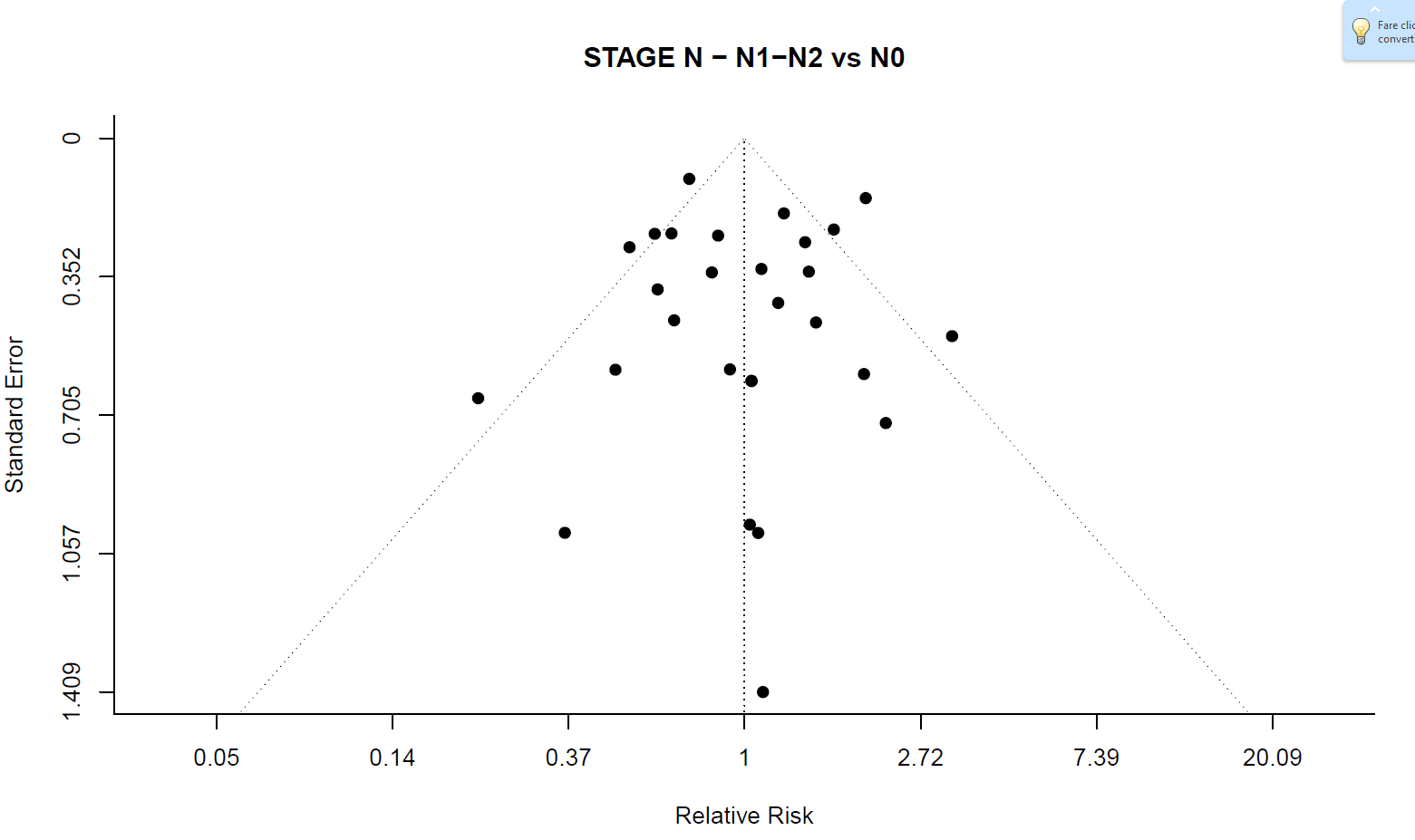


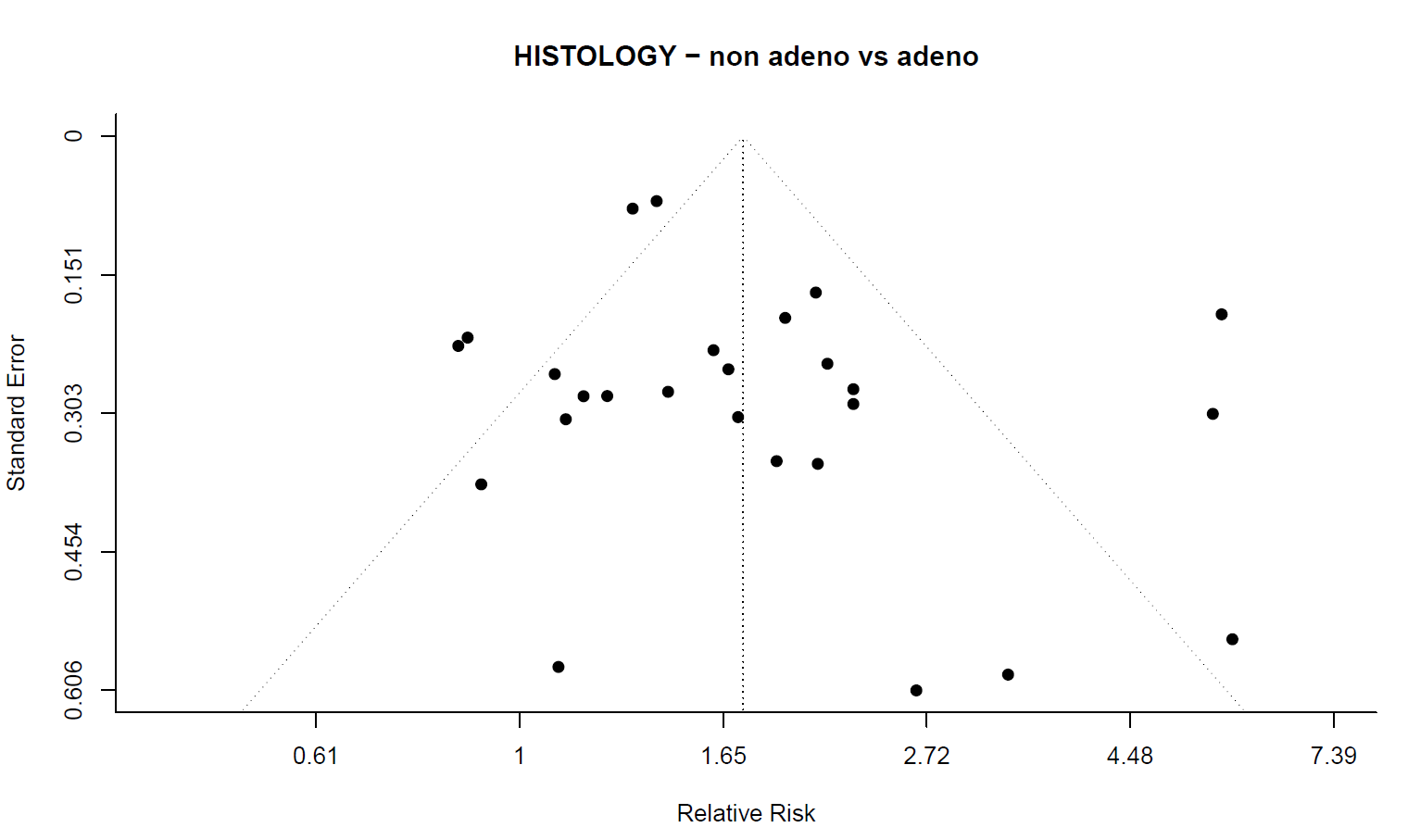


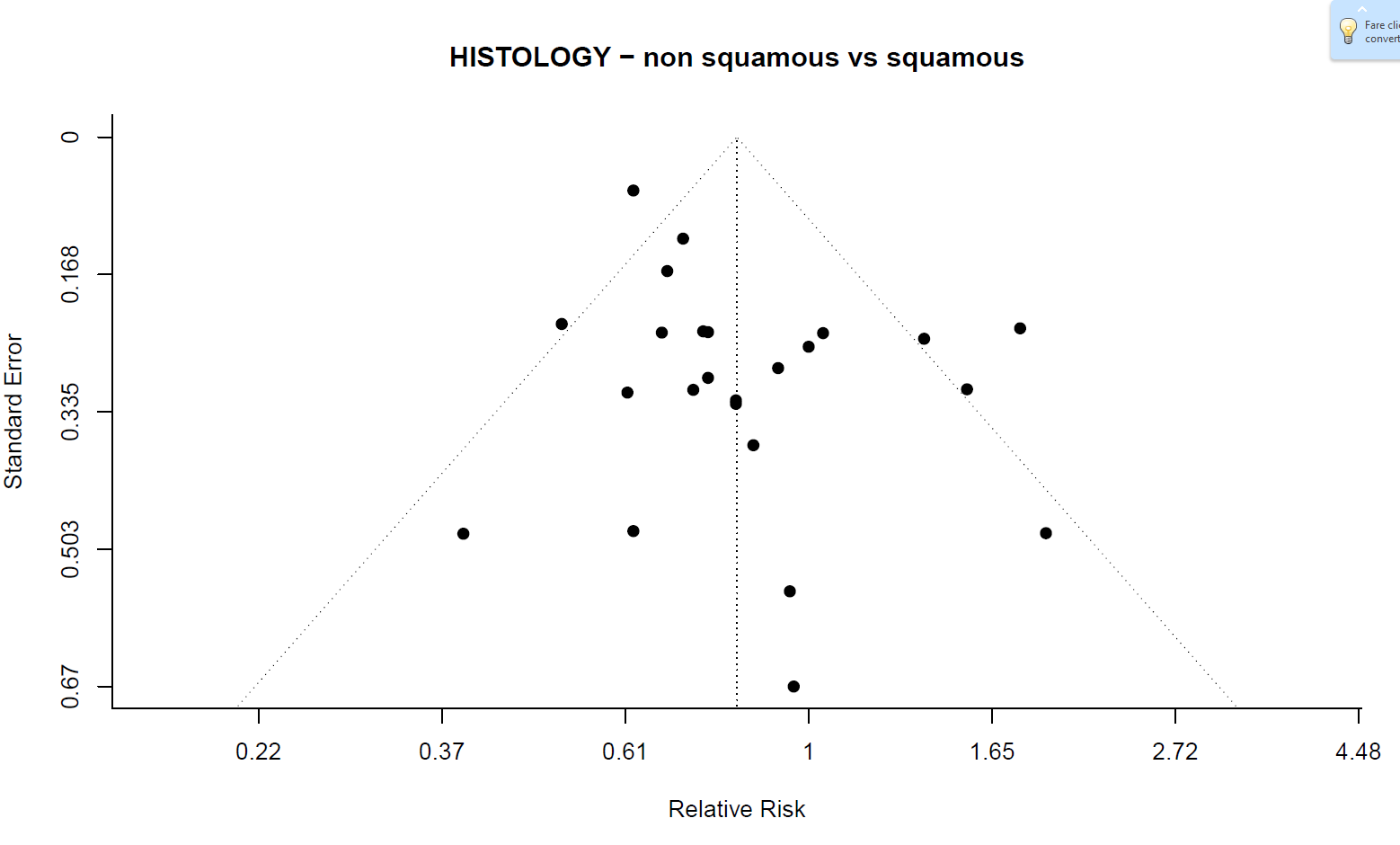


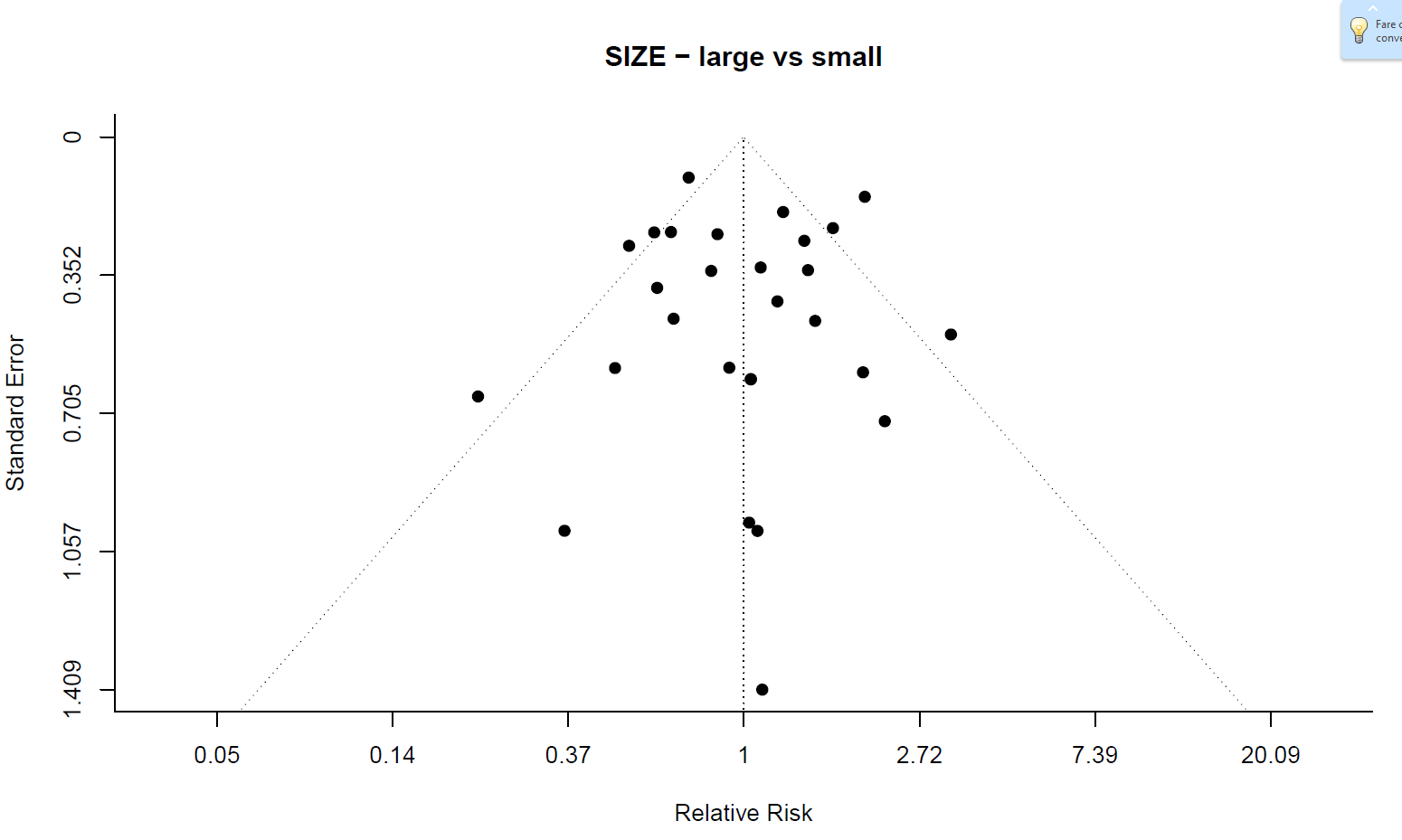


**Supplementary Figure 3.** Forest plots of studies on the association between selected risk factors and local/locoregional recurrence after surgical resection of non-small cell lung cancer.

LVI: lymphovascular invasion; Pstage: Pathological stage; VPI: Visceral pleural invasion.


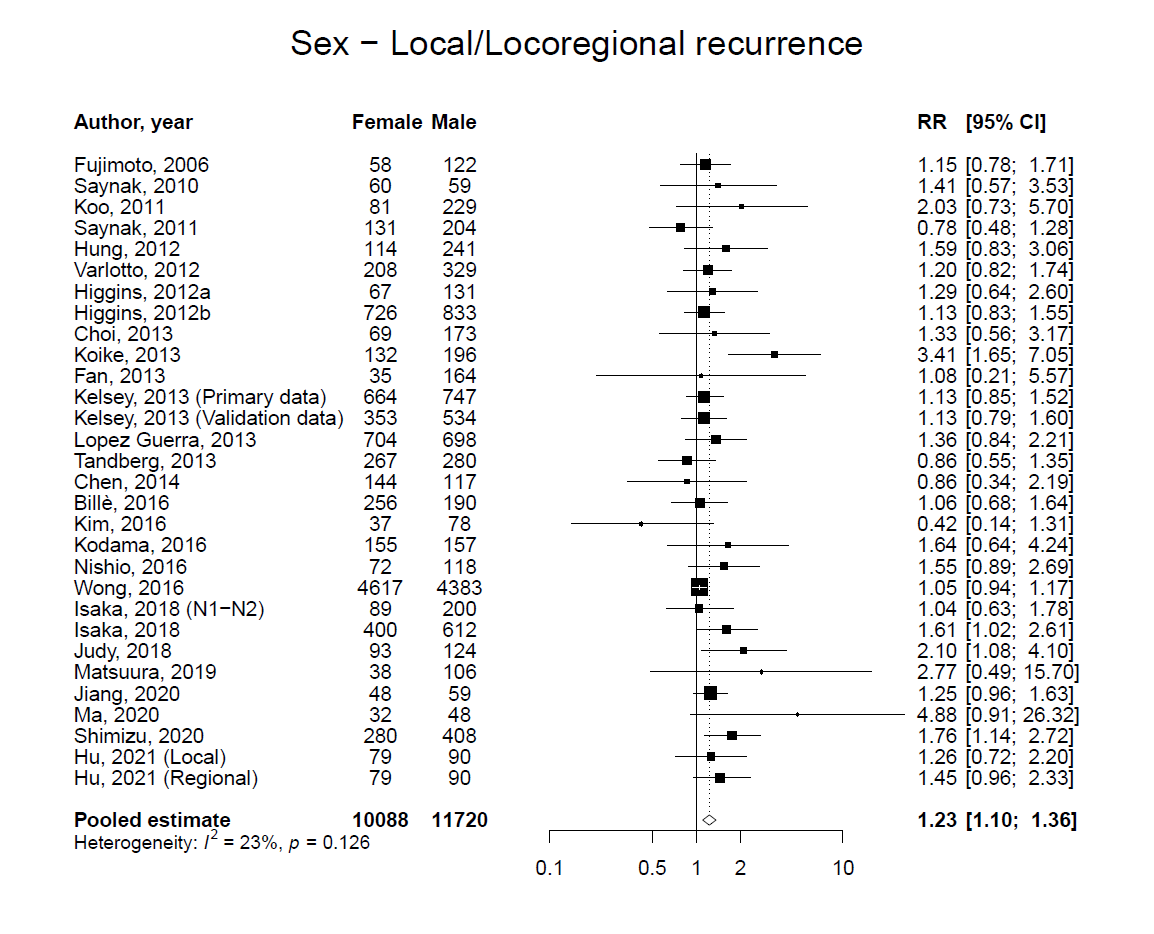


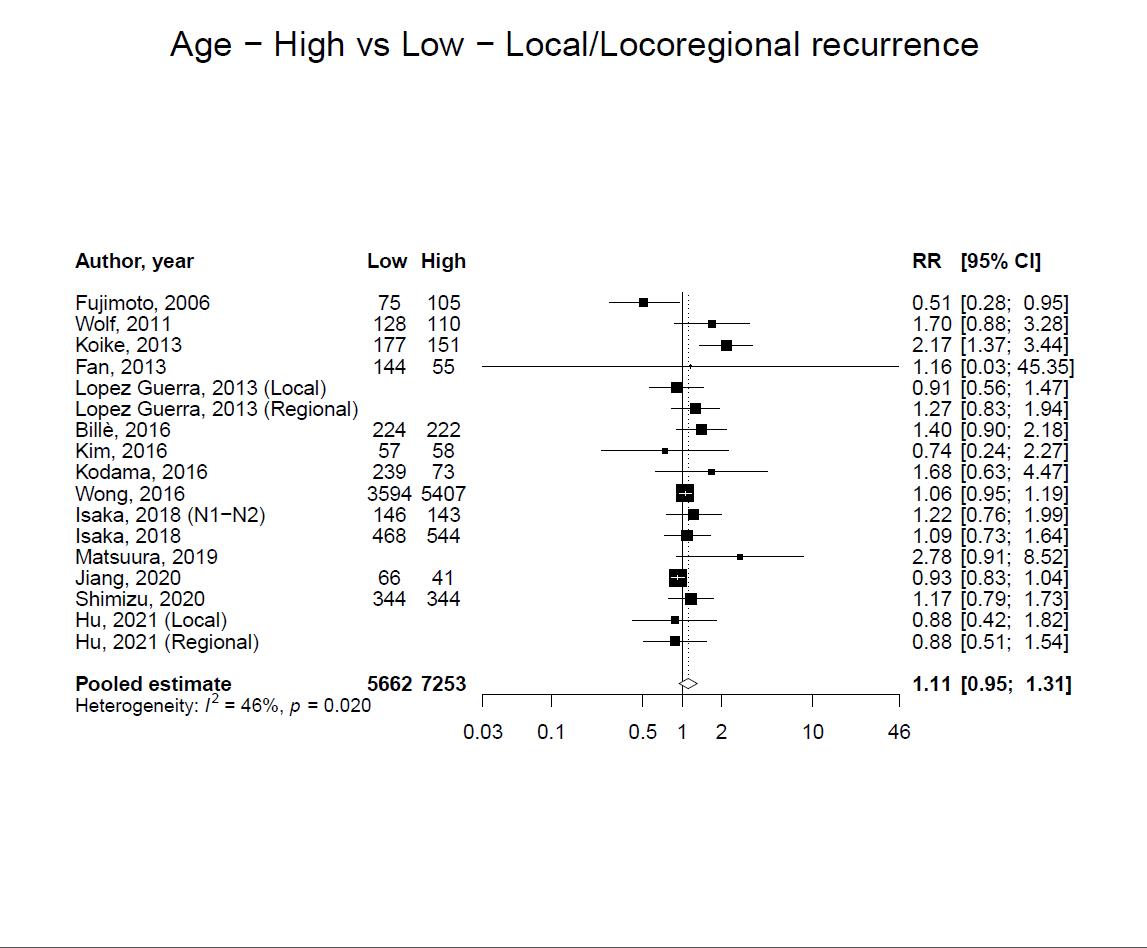


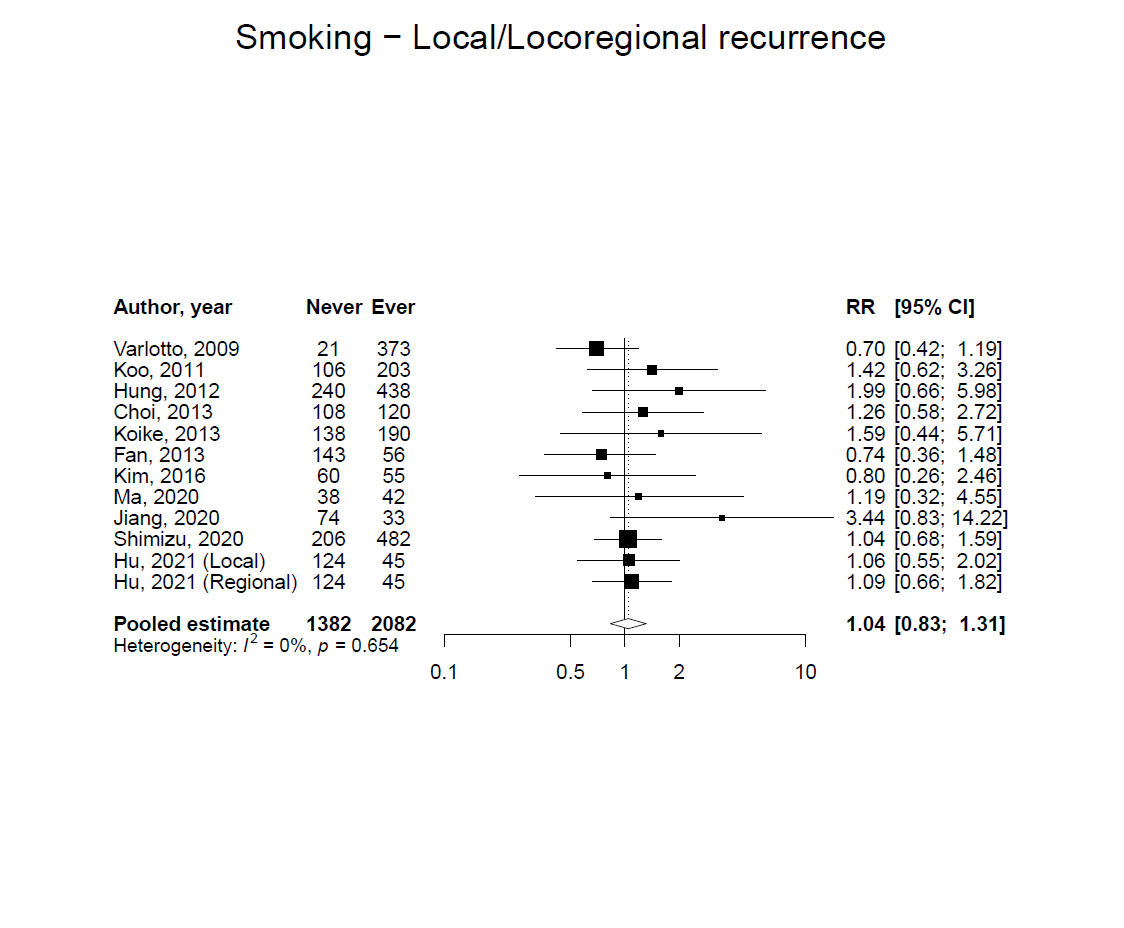


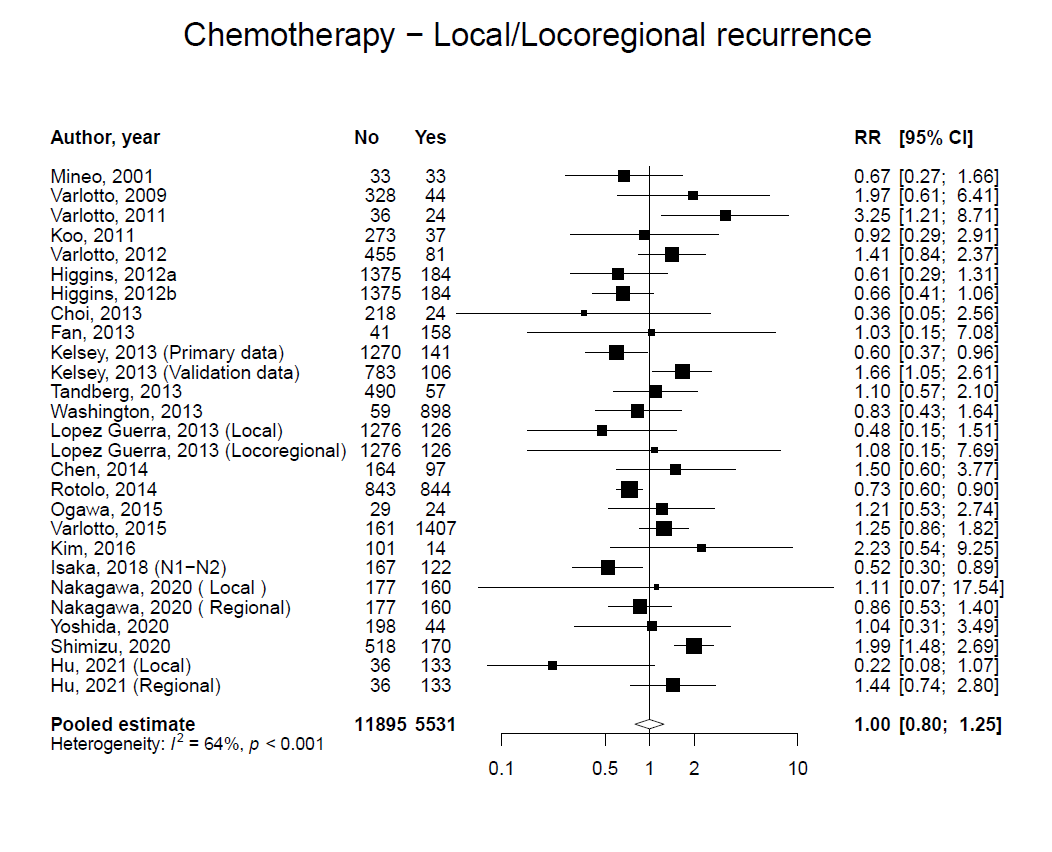


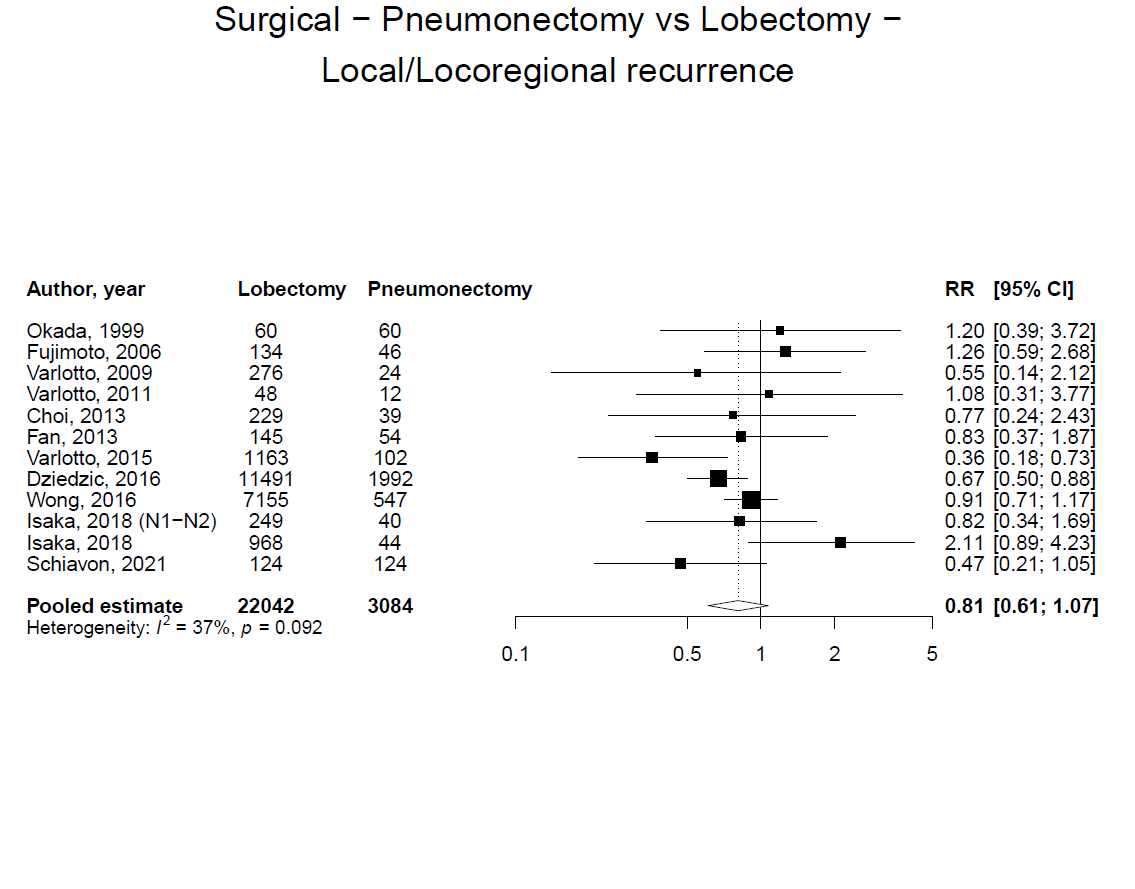

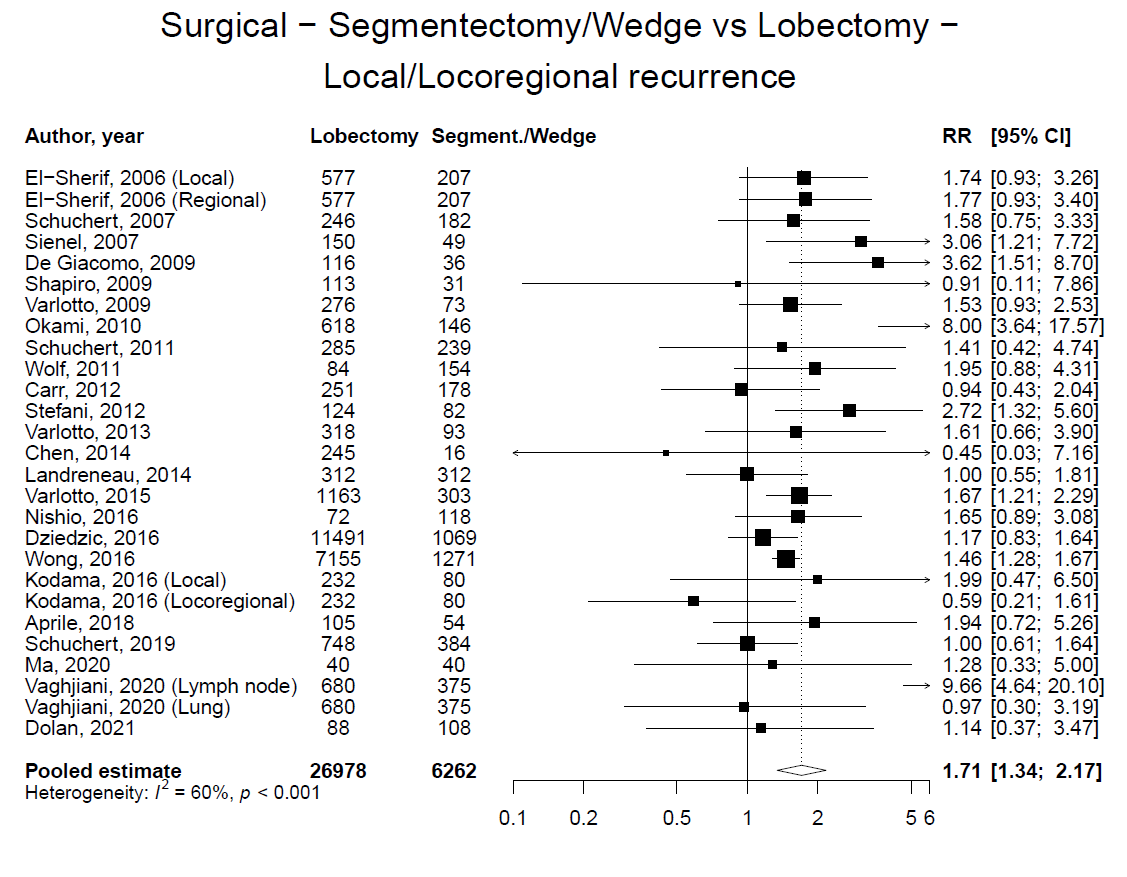


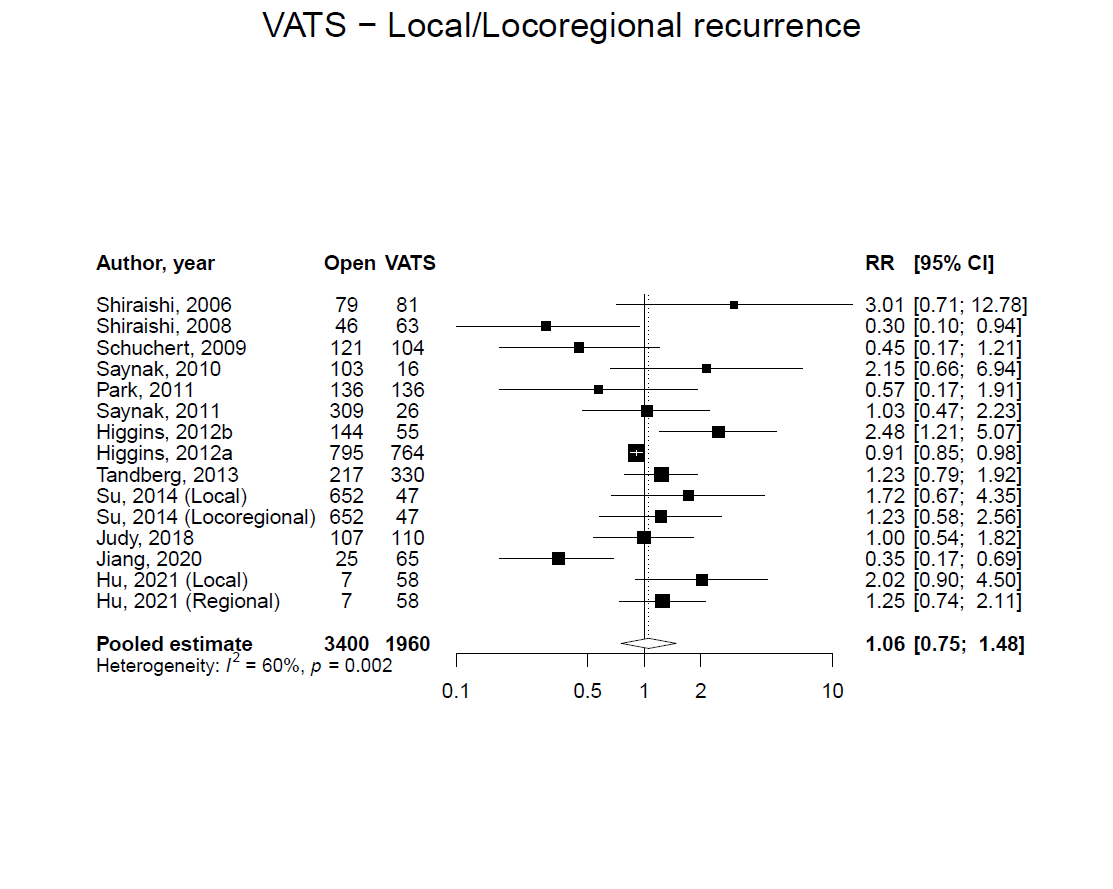


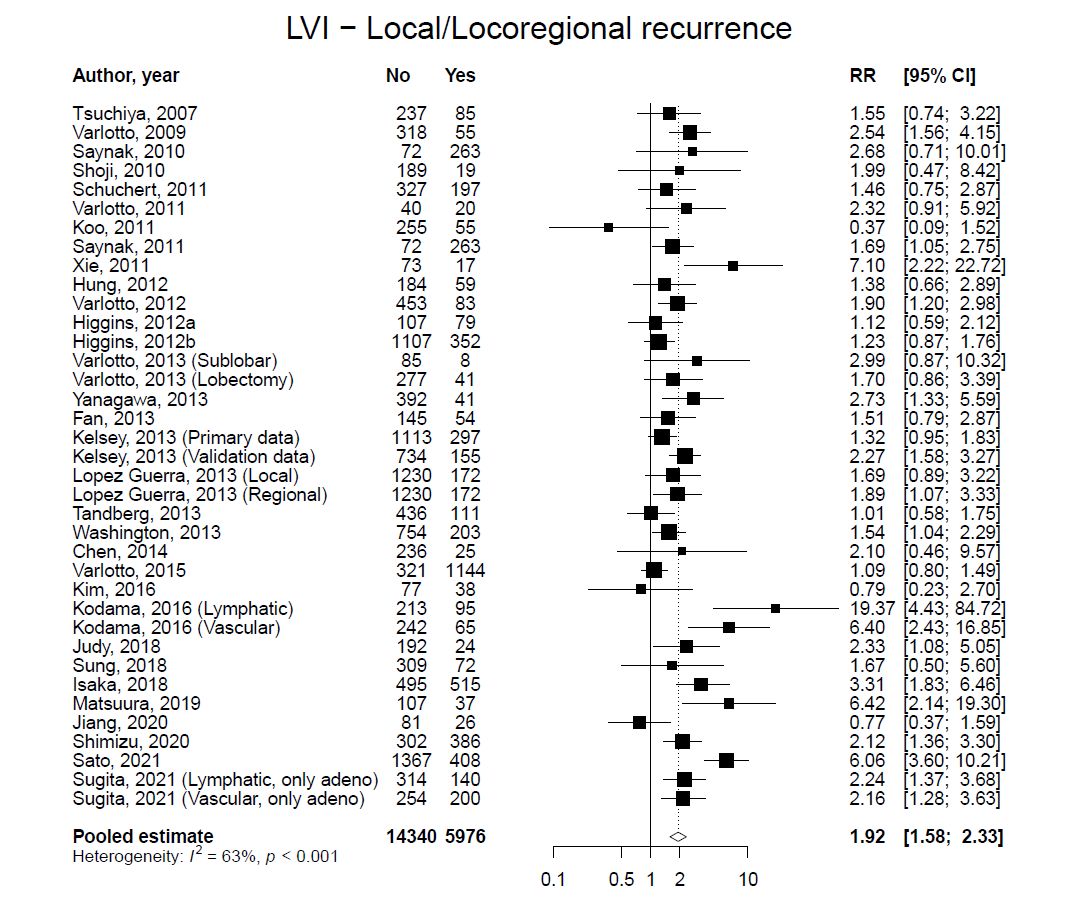

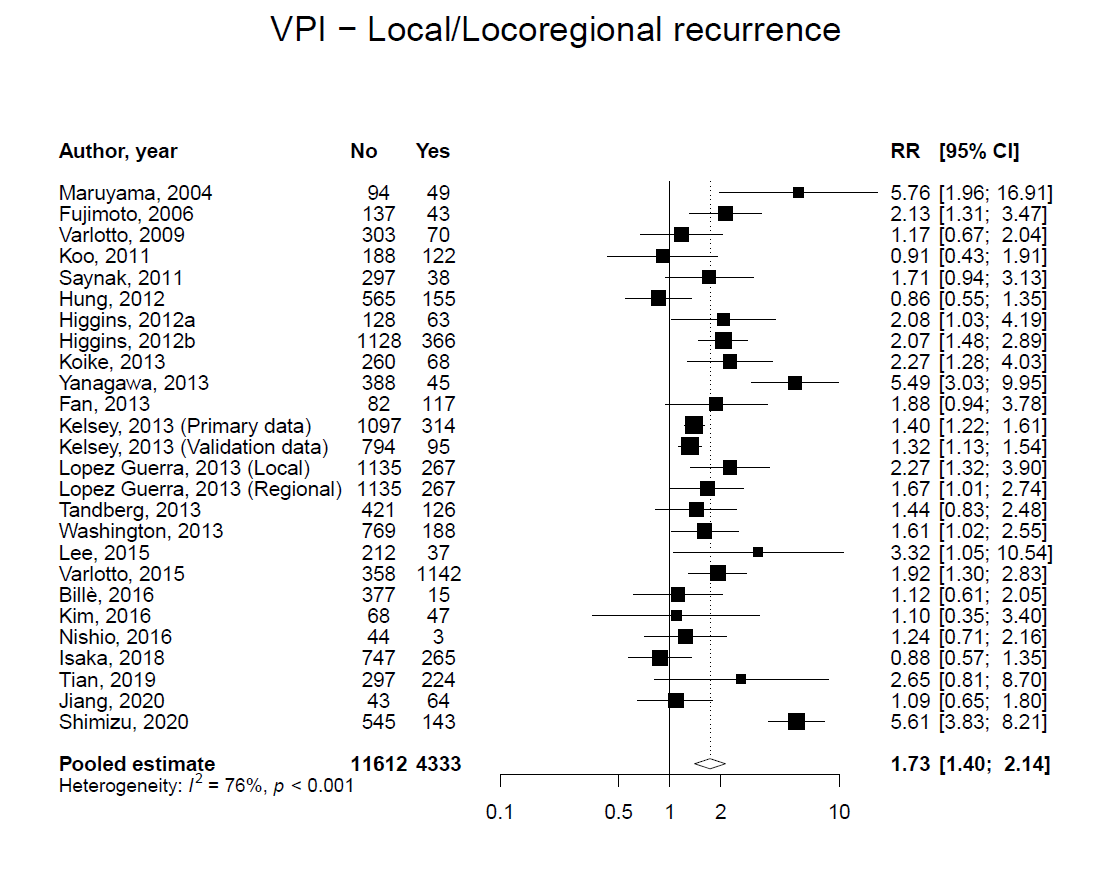

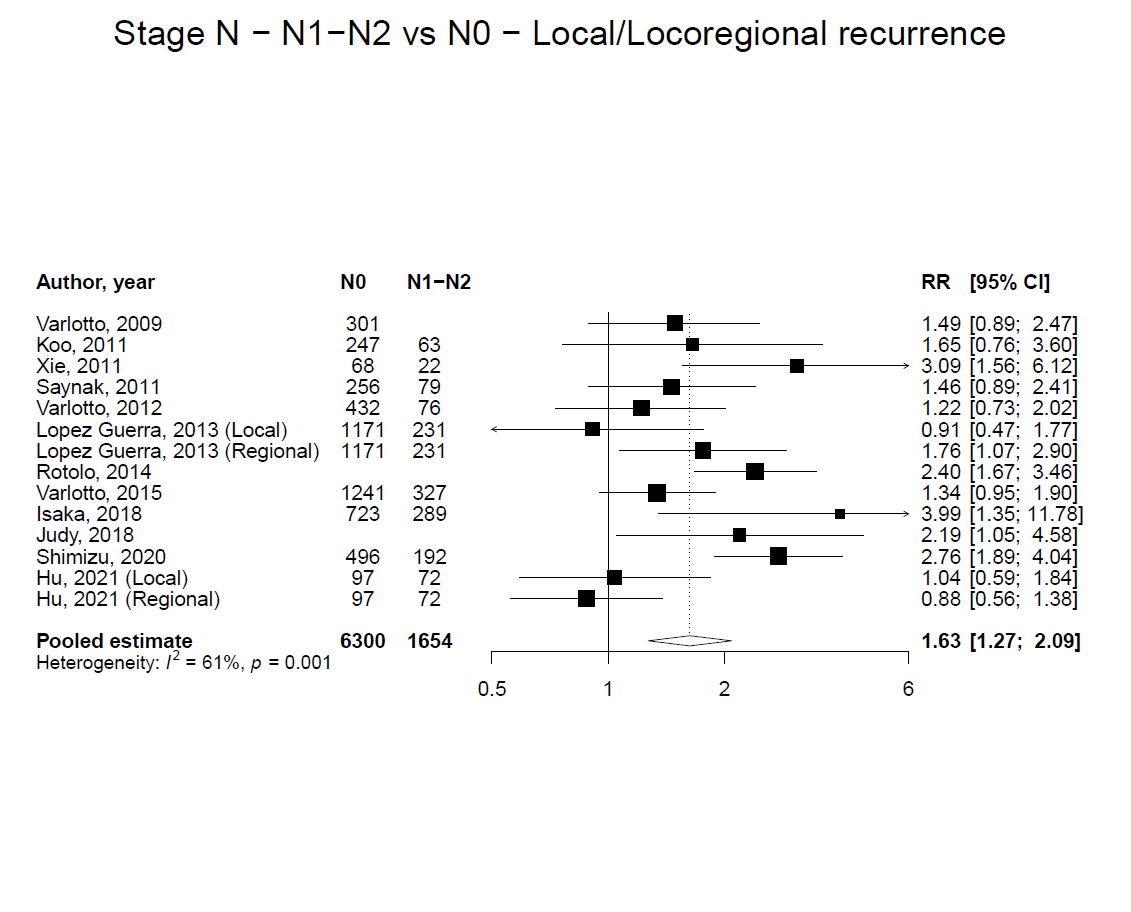

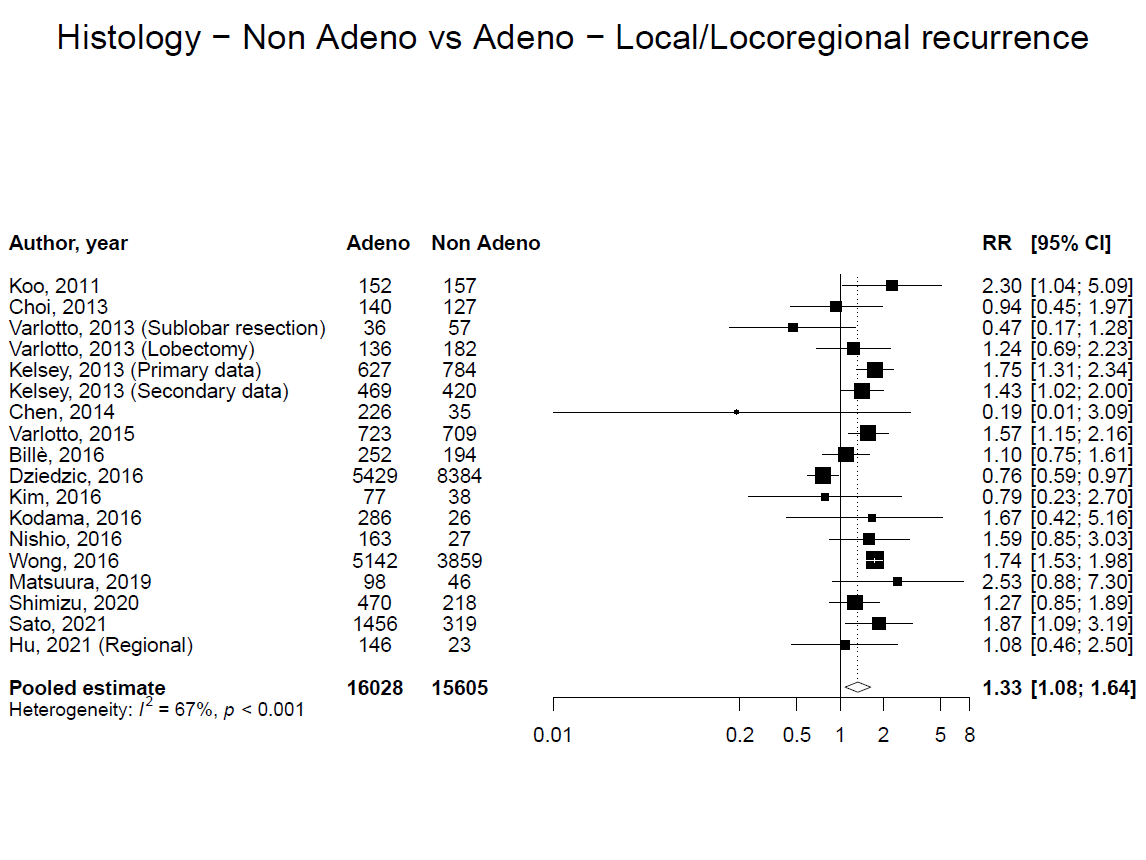

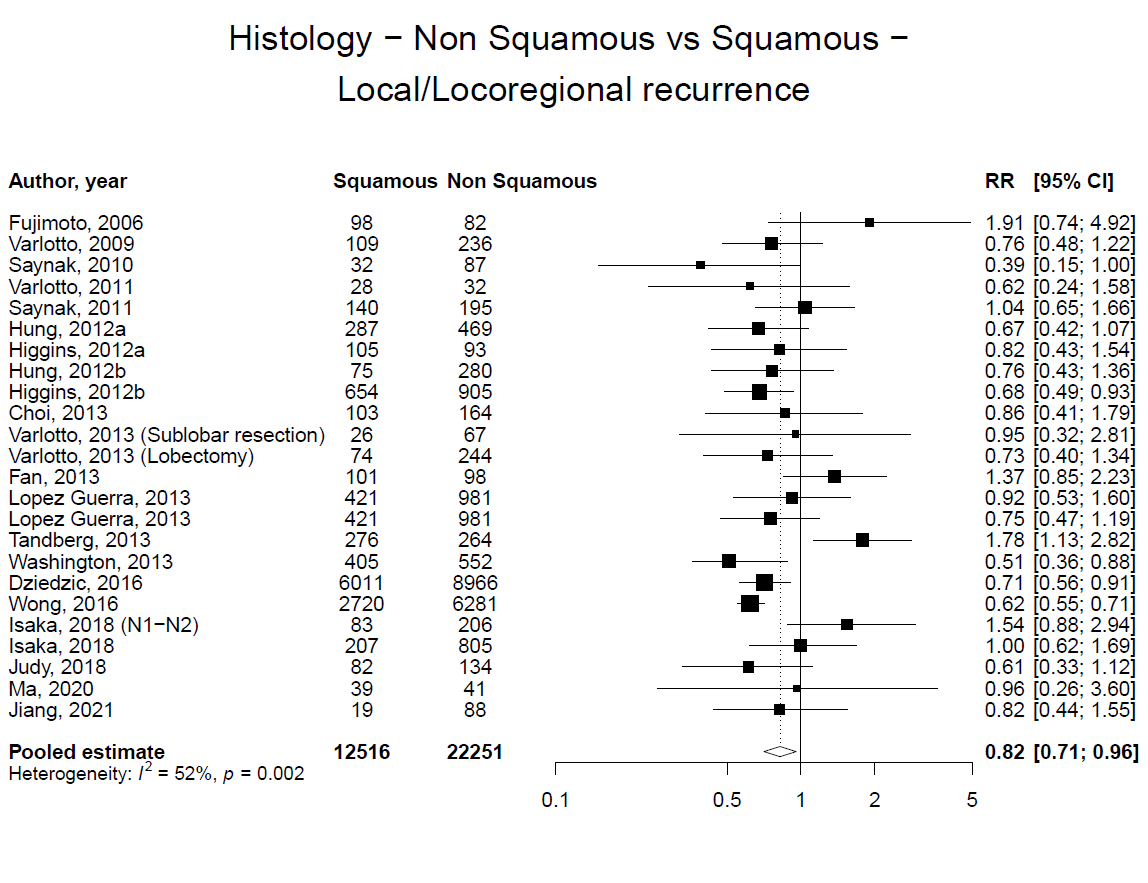

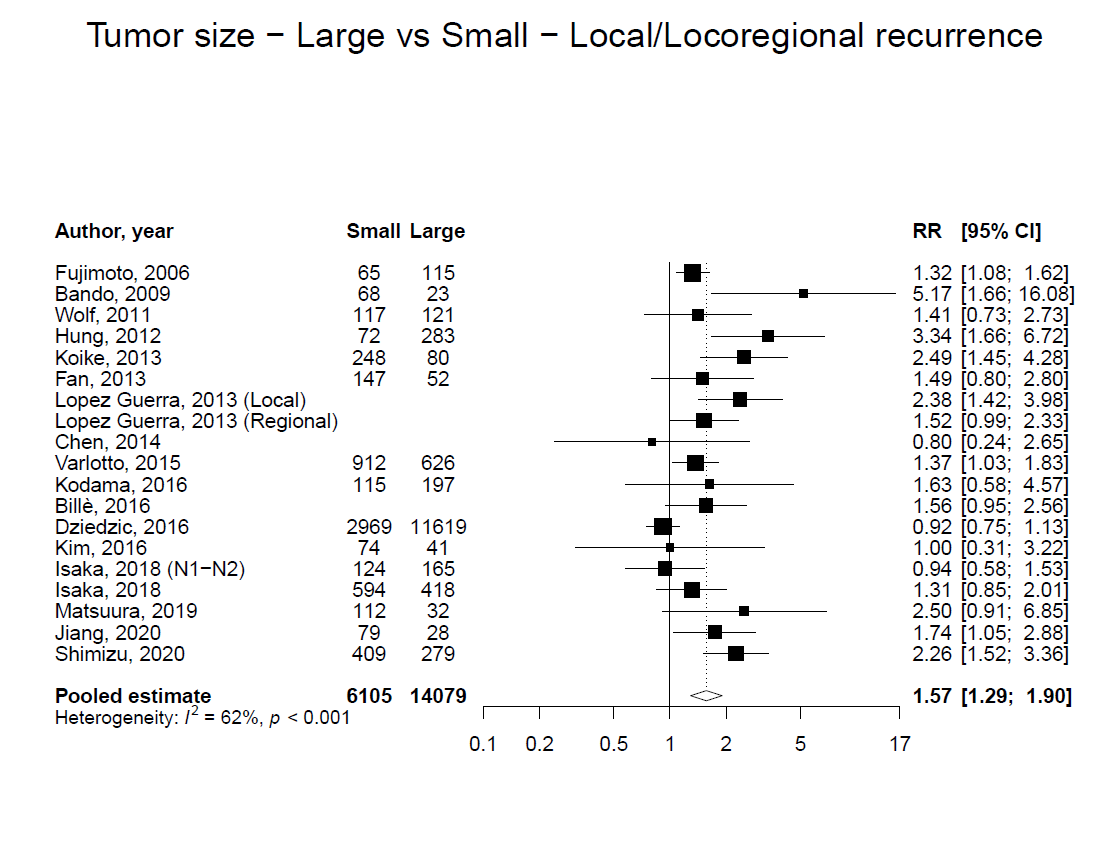
**Supplementary Figure 4.** Summary score of the study quality according to the Newcastle-Ottawa scale.

**Supplementary Figure 5.** Weighted box-plot locoregional recurrence percentage by staging subgroups


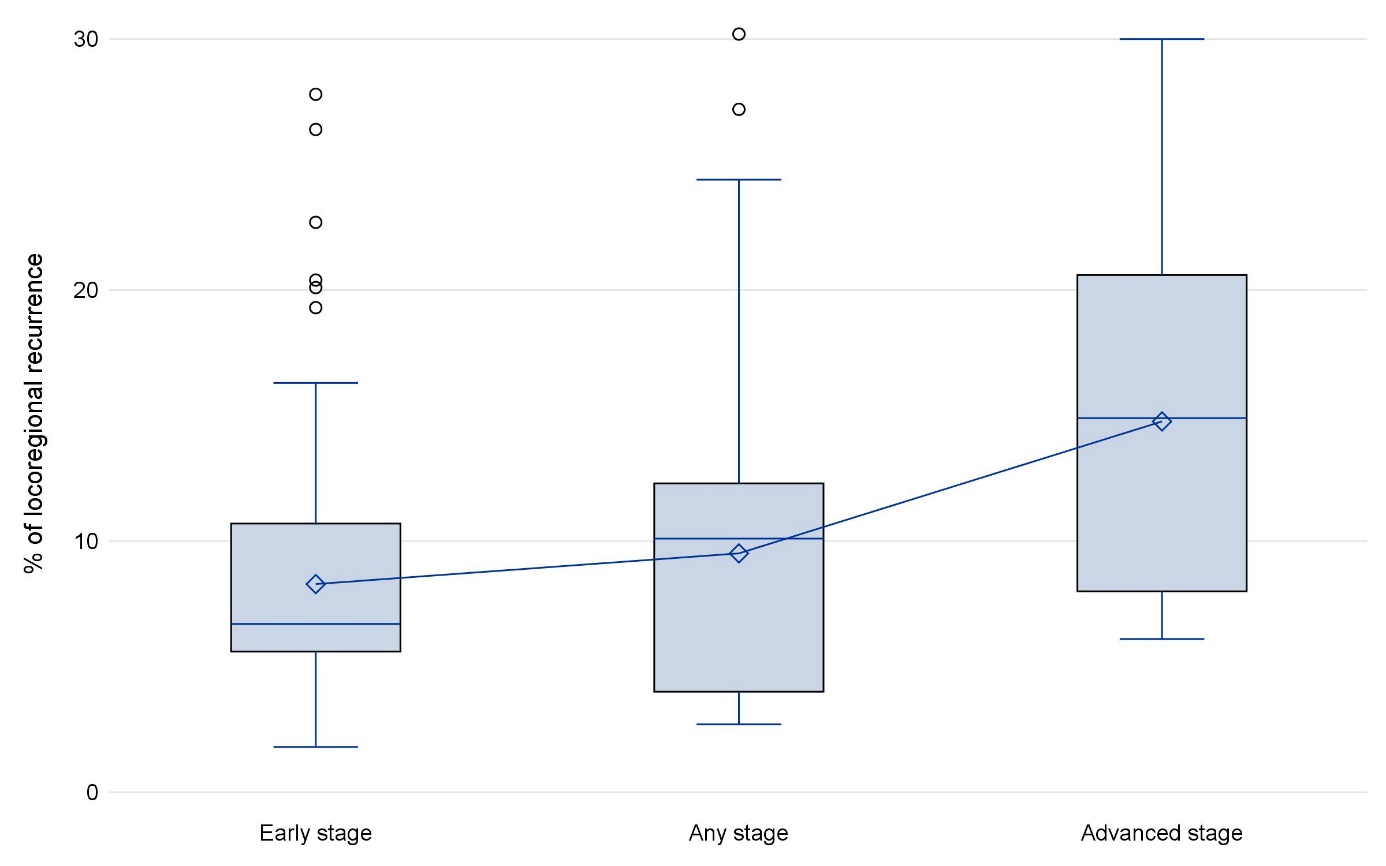


**Supplementary Figure 6.** Weighted box-plot locoregional recurrence percentage by recurrence definition


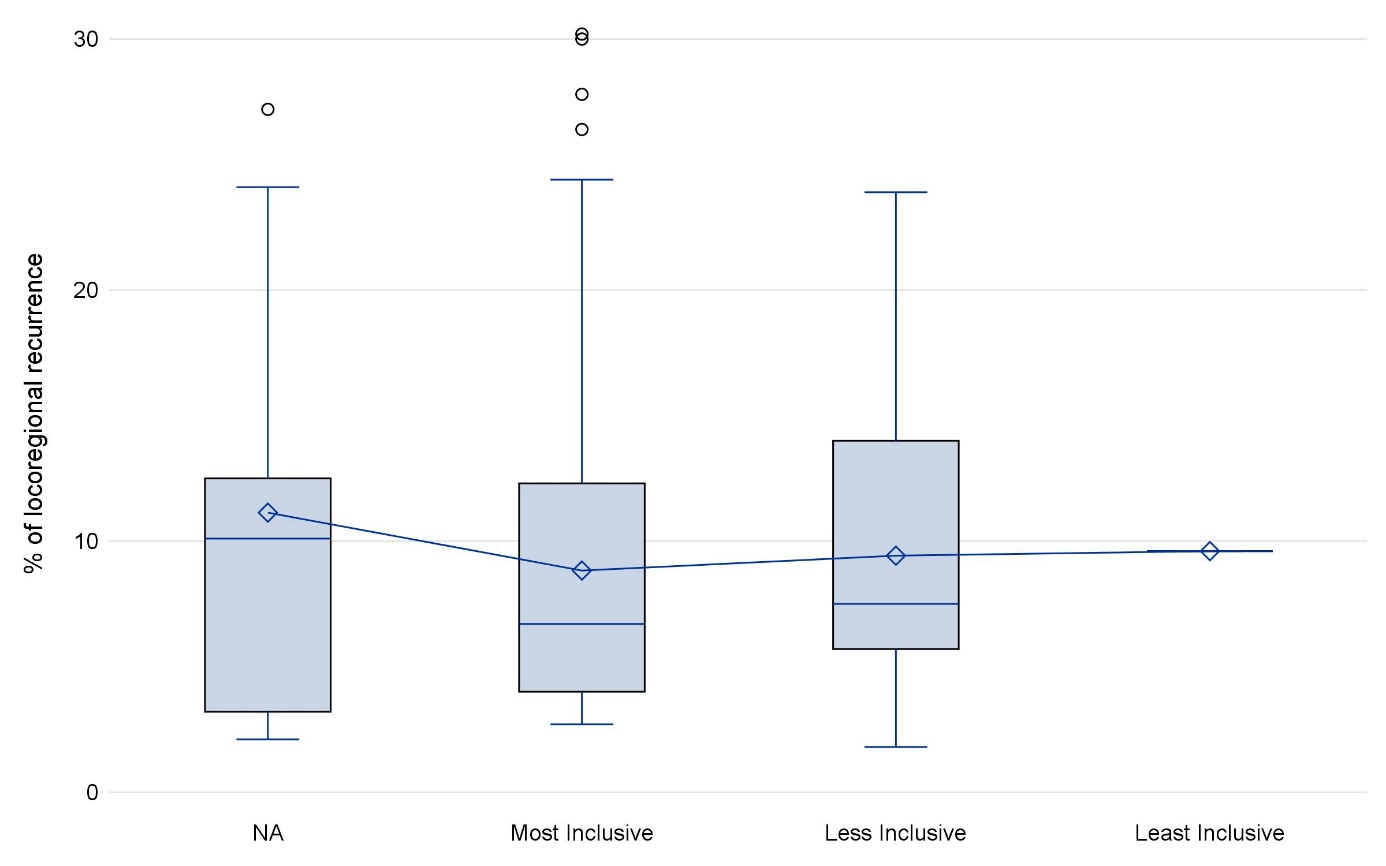


**Supplementary Figure 7.** Weighted box-plot distal recurrence percentage by staging subgroups


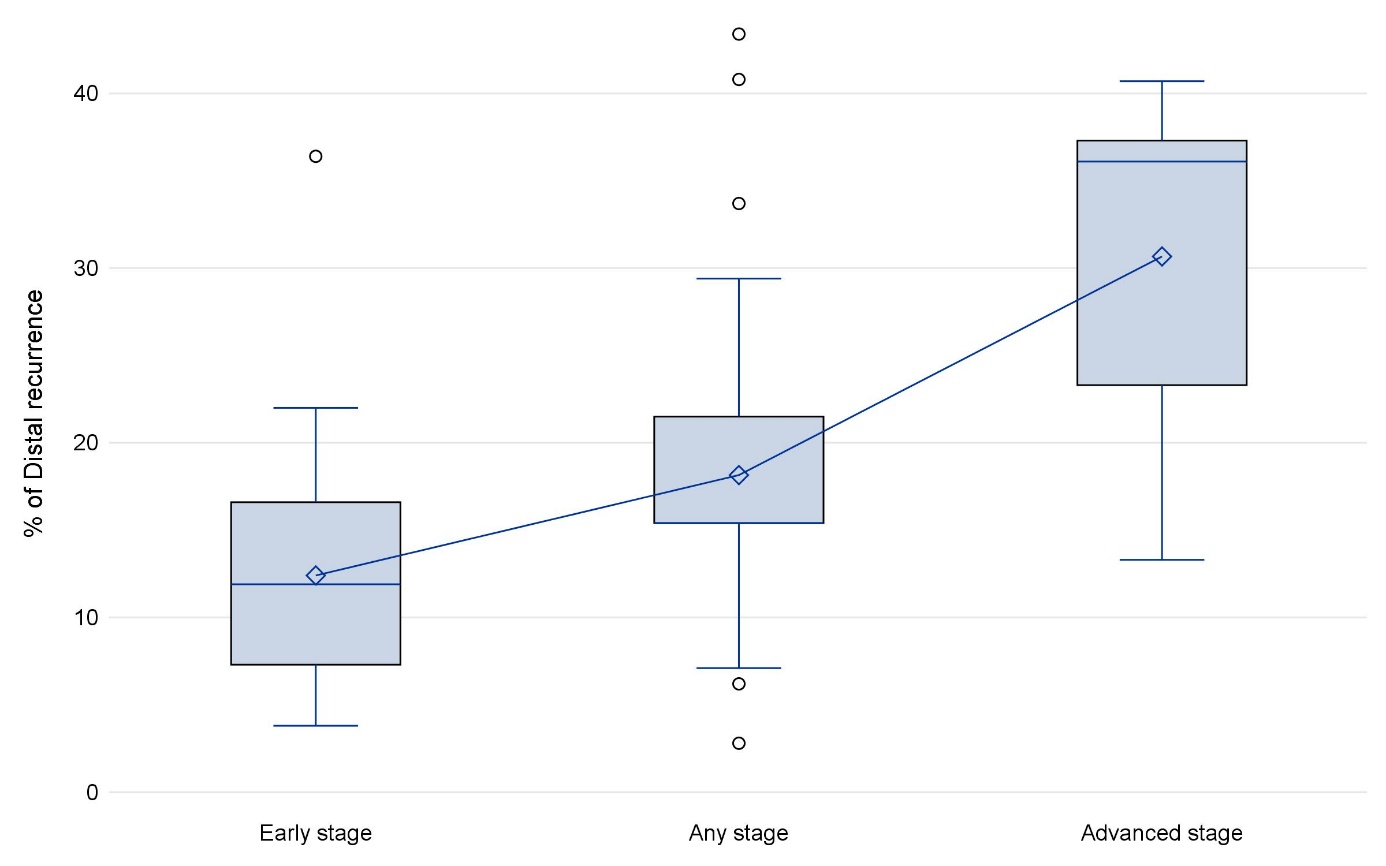


**Supplementary Figure 8.** Weighted box-plot distal recurrence percentage by recurrence definition


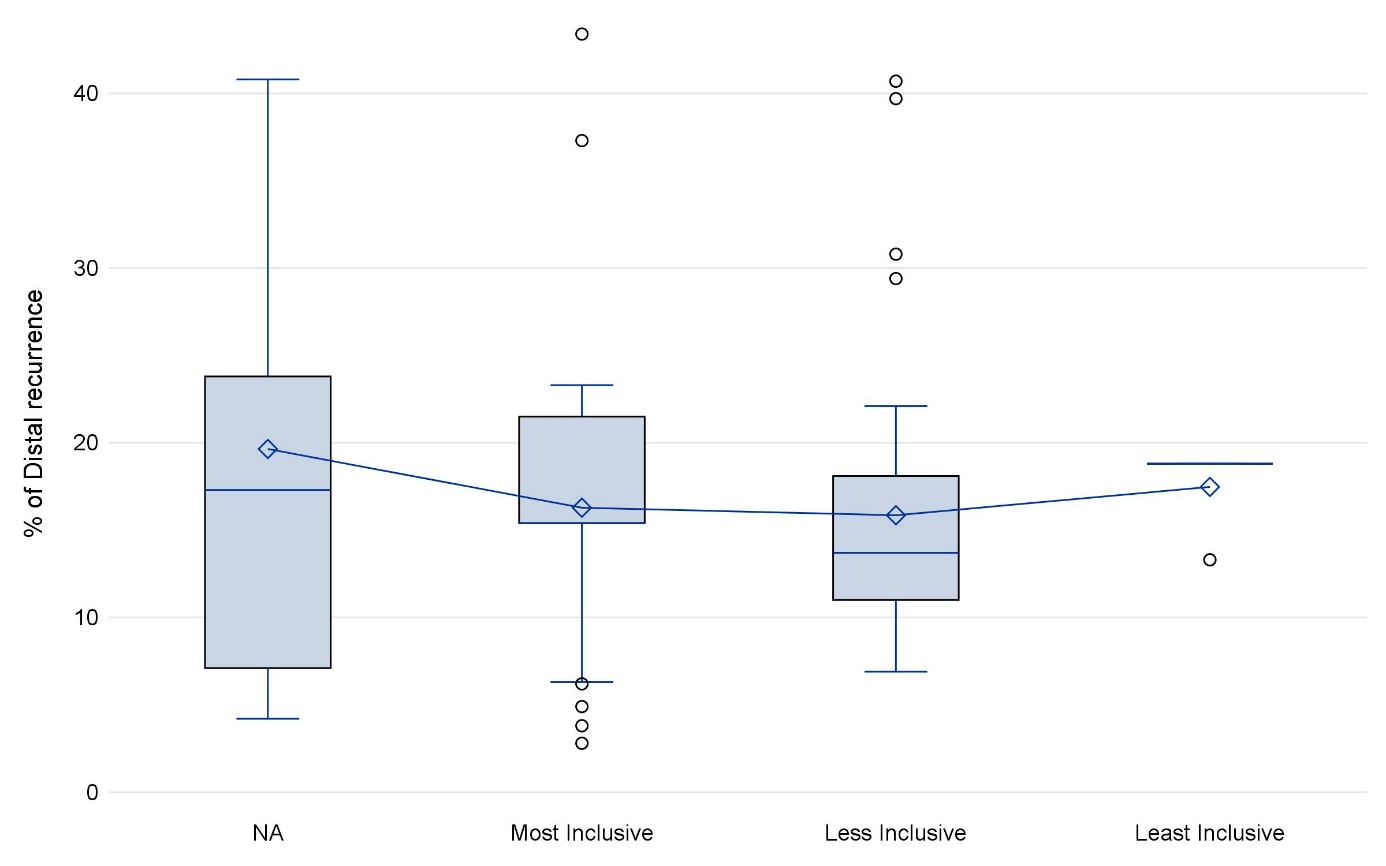

Supplement: Supplementary Tables and Figures [file mmc1.docx]
